# Accuracy of a 2-minute eye-tracking assessment to differentiate young children with and without autism

**DOI:** 10.1186/s13229-025-00670-4

**Published:** 2025-07-10

**Authors:** Kristelle Hudry, Lacey Chetcuti, Diana Weiting Tan, Alena Clark, Alexandra Aulich, Catherine A. Bent, Cherie C. Green, Jodie Smith, Kathryn Fordyce, Masaru Ninomiya, Atsushi Saito, Shuji Hakoshima, Andrew J. O. Whitehouse

**Affiliations:** 1 Department of Psychology, Counselling and Therapy, School of Psychology and Public Health, La Trobe University, Melbourne, Australia; 2 Speech Pathology Division, School of Allied Health, Human Services and Sport, La Trobe University, Melbourne, Australia; 3 Department of Psychiatry and Behavioral Sciences, Stanford University, Stanford, California USA; 4 CliniKids, The Kids Research Institute Australia, Perth, Australia; 5 School of Psychological Science, University of Western Australia, Perth, Australia; 6 Macquarie School of Education, Macquarie University, Sydney, Australia; 7 St Giles Society Limited, Burnie, Australia; 8 JVCKENWOOD Corporation, Yokohama-shi, Kanagawa Japan

## Abstract

**Background:**

Eye-tracking could expedite autism identification/diagnosis through standardisation and objectivity. We tested whether *Gazefinder* autism assessment, with Classification Algorithm derived from gaze fixation durations, would have good accuracy (area under the curve [AUC] ≥ 0.80) to differentiate 2-4-year-old autistic from non-autistic children.

**Methods:**

Community sampling (March 2019-March 2021) of 2:00–4:11 year-olds included children recruited into a diagnosed Autism Group (‘cases’) and Non-Autism ‘Control’ Group (with likely undiagnosed autism minimised). We recruited well beyond minimum necessary sample size to ensure within-group heterogeneity and allow exploratory subgroup analysis. Alongside *Gazefinder* eye-tracking attempted with all recruited participants, we collected parent-report measures for all children, and clinical/behavioural measures with autistic children.

**Results:**

102 autistic (81.4% male; *M*_*age*_= 44mths; *SD* = 8.8) and 101 non-autistic children (57.4% male; *M* = 40; *SD* = 10.5) were recruited and eligible; the former slightly older, proportionately more male, and reflecting greater socio-demographic diversity. *Gazefinder* autism assessment was completed with 101 non-autistic children (*n* = 1 returning minimal data), and attempted with 100- and completed with 96 autistic children (*n* = 2 not attempted following adverse responses to clinical testing; *n* = 4 attempted but unable to calibrate). The Non-Autism Group returned significantly more overall tracking data. The final Classification Algorithm (range 0-100; threshold score = 28.6)—derived from *n* = 196 children’s fixation durations to elements of social/non-social scenes, human face presentations, and referential attention trials—had AUC = 0.82 (sensitivity = 0.82, specificity = 0.70). Compared to those correctly classified, autistic children misclassified as ‘controls’ showed greater overall tracking, and less pronounced autism features and developmental disability. Compared to correctly classified non-autistic children, those misclassified as ‘cases’ were older with lower overall tracking.

**Limitations:**

Our groups differed on socio-demographic characteristics and overall tracking (included within the Classification Algorithm). We used the ‘Scene 10A’ stimulus set as provided, without update/modification. Industry employees who developed the final Algorithm were non-blinded to child group, and considered only gaze fixation durations. Community sampling and ‘case-control’ design—comparing diagnosed autistic vs. non-autistic children—could be improved via future referral-based recruitment.

**Conclusions:**

Most children tolerated *Gazefinder* autism assessment, and our Classification Algorithm properties approached those reported from other *Gazefinder* use and established clinical assessments. Independent replication is required, and research informing the most suitable clinical application of this technology.

**Trial registration:**

ACTRN12619000317190

**Supplementary Information:**

The online version contains supplementary material available at 10.1186/s13229-025-00670-4.

Autism Spectrum Disorder (hereafter, autism) is a neurodevelopmental condition characterized by core social-communication and behavioral flexibility difficulties [1] with highly variable clinical presentation and developmental course [2]. Early intervention can support later-life outcomes but is typically available only following diagnosis [3, 4]. Early signs often present before a child’s first birthday, becoming increasingly apparent across the second year of life [5] albeit with diagnosis not typically until age 3- to 4-years at the earliest [6, 7] despite research showing ascertainment is possible—and highly stable—from age 2-years [8]. Autism diagnosis involves the clinical appraisal of developmental history and contemporaneous behavioural presentation against seven broad criteria [1]. If feasible and accurate, technology-supported assessment could bring objectivity and efficiency to an often unnecessarily protracted process that exacerbates caregiver distress and delays child access to supports [9–12].

Eye-tracking technology shows strong potential to support autism diagnosis, leveraging visual attention behaviours that differentiate autistic/non-autistic people across the lifespan [13]. Reduced preferential attention toward social (vs. non-social) scenes [14, 15] and reduced fixation time toward more (vs. less) socially-salient/-informative regions of interest (ROIs) within scenes [16–18] have been robustly observed among autistic vs. non-autistic adolescents/adults [19, 20] and children [21, 22] and from very early life among later-diagnosed infants [23]. While even ‘gold-standard’ diagnostic tools—the Autism Diagnostic Observation Schedule (ADOS) [24] and Autism Diagnostic Interview-Revised (ADI-R) [25]—retain a degree of subjectivity in administration and scoring, eye-tracking offers greater objectivity. Further, the robust nature of visual attention differences means a standardised eye-tracking assessment may plausibly be far briefer than other assessments that seek comprehensive appraisal of behavioural autism presentation. Finally, requiring only the capacity to remain seated and sustain attention to on-screen stimuli, eye-tracking has clear potential for use with individuals whose young age and/or complex support needs may limit tolerance of longer, interactive assessments.

The *Gazefinder* autism assessment was developed by JVCKENWOOD Corporation (JKC) and Japanese colleagues [26, 27] to meet the perceived need for a brief, standardised ‘off-the-shelf’ assessment of visual attention to support identification and diagnosis. Recent studies show its feasible use with autistic and non-autistic children, adolescents and adults [26, 28, 29], and with clinically-indicated infants showing early signs of autism [30], and that *Gazefinder* autism assessment broadly replicates findings of differentiated preferential social attention by viewers with/without autism reported with the use of other eye-tracking technologies and stimulus sets [19, 26]. Two existing studies describe the performance properties of *Gazefinder* autism assessment algorithms developed to differentiate autistic/non-autistic viewers. Among an adolescent/adult sample, a best-fit algorithm differentiated 26 autistic from 35 non-autistic viewers with 81% sensitivity and 80% specificity (overall accuracy and Area under the Curve [AUC] not reported) [19], comparable to properties of relevant ADOS-2 modules (see Additional File [AF], Table AF10) [24]. Recently, a *Gazefinder* autism assessment and best-fit algorithm has also been reported to differentiate 39 autistic from 102 non-autistic children aged 5- to 17-years, with 78% overall accuracy (AUC; 74% sensitivity; 80% specificity) [27].

We trialled the feasible use of *Gazefinder* autism assessment, and the accuracy of a best-fit algorithm to differentiate individuals with/without autism, during the early childhood period when diagnostic assessment often occurs; between ages 2- to 4-years. With recent evidence suggesting good classification accuracy for older child-aged, and adolescent/adult samples—and feasible use of *Gazefinder* from infancy [26, 30]—we pre-specified the hypothesis that a single ‘best-fit’ algorithm derived from gaze fixation data captured during the brief, standardised *Gazefinder* autism assessment would show at least ‘good’ classification accuracy (operationalised as AUC ≥ 0.80) [31].

## Methods

### Design, setting and oversight

This case-control study—prospectively registered on 1st March 2019 with the Australian New Zealand Clinical Trials Registry (ACTRN12619000317190)—was conducted at three Australian sites, with ethical approval (HEC19027), trial sponsorship and Data Monitoring and Safety Committee review provided via La Trobe University (LTU). Funding for the trial was from developer/manufacturer, JKC, who also engaged a third-party contractor for regular independent audit of trial conduct. Recruitment and data collection occurred between March 2019 and March 2021 with most assessments conducted at Melbourne- and Perth-based clinical research settings. Following recruitment challenges experienced across 2020 with the COVID-19 global pandemic, data collection was extended to community clinical/education centres in North-Western Tasmania in 2021.

Parents/guardians provided signed informed consent for each child to participate in a single timepoint assessment. For all children, this included the *Gazefinder* eye-tracking assessment and collection of parent-report measures. For the Autism Group only, additional direct behavioural assessments were completed (at the same visit wherever possible, but with additional appointment/s as needed to minimise missing data) or accessed (if recently completed for other research/service access; specifically, autism measure within 6-months and developmental/cognitive or parent-report measures within 4-weeks) to reduce unnecessary repeat-assessment burden.

### Participant recruitment

Children were recruited as potential ‘cases’ (i.e., children with a community autism diagnosis; see note^1^ and citation [32]) or ‘controls’ (i.e., children without autism/other parent-reported developmental conditions), through broad and targeted advertising via relevant community networks. Eligibility criteria were chronological age 24–49 months (at *Gazefinder* autism assessment) and no significant uncorrected visual/hearing impairment. The single additional inclusion criterion for the Autism Group was a community diagnosis evidenced via assessment report/letter sighted by trial researchers. To avoid artificially constraining heterogeneity (e.g., regarding the presence of co-occurring conditions, medication use, service access, etc.) no other eligibility criteria were set. For the Non-Autism Group, the following exclusion criteria were stipulated to minimise the likelihood of undiagnosed autism: any parent-reported developmental concerns; autism diagnosis in a biological parent/sibling; and/or signs of possible undiagnosed/sub-threshold autism in the child (operationalised as Social Communication Questionnaire [SCQ] [33] score > 12).

### Study size

A-priori MedCalc [34] calculation indicated that 40 participants would provide 95% power for a test of ‘good’ vs. chance-level accuracy (i.e., AUC = 0.80 vs.50) [31] assuming equal group size and *α* = 0.05. However, rather than recruiting small, closely matched groups, we planned substantial over-recruitment to: (a) maximise likely population-representative phenotypic heterogeneity in the Autism Group and ensure a sizeable female subgroup; (b) permit adequately powered exploratory analysis of correctly- vs. mis-classified participants; and (c) mitigate against potential missing data. We set recruitment targets to 100 per group, offering > 95% power for test of ‘excellent’ vs. ‘good’ classification accuracy (i.e., AUC = 0.90 vs.80) [31].

### Exposure and primary outcome measurement

*Gazefinder* is a desktop eye-tracker that uses infrared light to determine corneal reflection and identify gaze fixations and eye movement saccades [19, 26]. Apparatus hardware and system details are provided in the online Additional File (Figures AF1-5) along with software details specific to the autism assessment sequence used here, referred to by its developers as ‘Scene 10A’ (Figures AF6 & AF7; Tables AF1 & AF2. Briefly, the *Gazefinder* device uses corneal reflection techniques to determine the eye gaze of participants seated at around 60 cm from a 19-inch monitor (1280 × 1024 pixels), to coordinates (X, Y) in pixel units, sampled at 50 Hz (i.e., 3,000 datapoints per minute) [27].

**Fig. 1:**
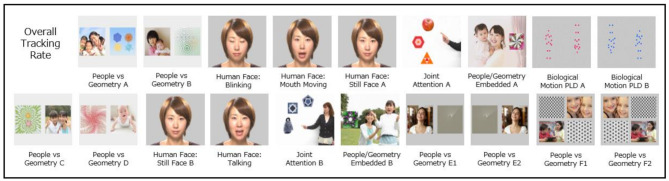
Key stimuli within *Gazefinder* ‘Scene 10A’ autism assessment, in order of presentation (*n.b.*, initial calibration sequence and between-trial attention grabbing animations not shown)

The *Gazefinder* ‘Scene 10A’ autism assessment included an initial on-screen visualisation used to confirm good child positioning relative to device (i.e., seated height; distance from screen), followed by a 5-point calibration sequence (~ 20–40 s, as needed), and brief assessment comprising 19 key animations interspersed with brief attention-grabbing stimuli (total time ~ 2 min 15 s) illustrated in Fig. 1. Key animations covered four main trial types:

1. *People* vs. *Geometry* (i.e., Social vs. Non-Social preferential attention) [14, 15]: Total of 10 trials—six with equally-sized paired stimuli, two with geometric patterns embedded within larger social scenes, two with four equally-sized stimuli (two each social and non-social) arranged within quadrants—with relative position of elements on screen counterbalanced;
2. *Eyes* vs. *mouth* [17, 23]: Total of five trials of the same human face, varying in terms of static/dynamic features, and with/without sound (i.e., blinking, mouth moving [silently]; still face [two trials, separated by other stimulus trials]; and talking);
3. *Biological motion* point-light display (PLD) type animations [16, 35]: Two trials of paired *upright* and *inverted* stimuli (counterbalanced for side of screen), noting that these were included in the animation for consistency with past published work [19] but ROIs for these stimuli were excluded from consideration for the classification algorithm given accumulating evidence that biological motion poorly differentiates autistic from non-autistic people [16, 19, 35–37]; and.
4. *Response to joint attention* (i.e., pointing) probes [18, 38]: Two trials each including one *target* and two *distractor* objects, and a *referential agent* (one trial showing pointing hand only; one trial showing agent’s full torso).

We confirm that images represented here were purchased or generated by JKC for use in the *Gazefinder* autism assessment, and that we have permission from JKC to include the images here (and in the online Additional File) for illustration, including in open-access publication.

Assessments were conducted in rooms selected for minimal distraction (e.g., no windows/toys) though assessors could relocate the device to locations where children might be better settled/more attentive. Children could view the *Gazefinder* autism assessment seated independently or on an adult’s lap. If initially unsuccessful, the calibration sequence was automatically re-attempted, and could thereafter be manually restarted/reattempted until successful calibration (i.e., detection of gaze to centre and 2 of 4 peripheral points) was achieved. *Gazefinder* then proceeded automatically to ‘Scene 10A’ presentation.

For each child, assessors recorded the number of calibration attempts, any adverse emotional/behavioural responses (to *Gazefinder-* and/or other assessments), and any other behaviours of note during *Gazefinder* autism assessment. Overall tracking rate and fixation durations to pre-determined ROIs within the 19 key stimulus scenes were automatically recorded by *Gazefinder* software. A single ‘best-fit’ *Gazefinder* Classification Algorithm was generated from raw data exported from the device following data collection (detailed below).

### Clinical characterisation

Socio-demographic information collected included child sex, age and gestational age, family composition, parental ethnicity, and household income, and also, for the Autism Group, other health/developmental conditions, medication use and intervention service access. Parents completed the Social Communication Questionnaire (SCQ) [33] for a measure of child behavioural autism presentation (Total Score range 0–40) with potential non-autistic controls excluded if SCQ Total Score > 12. Other parent-report measures collected for all children included: the Vineland Adaptive Behavior Scales (VABS) [39] offering a measure of functional independence skills (i.e., Adaptive Behavior Composite [ABC] Standard Score [SS; population *M* = 100, *SD* = 15]); the Child Behaviour Checklist (CBCL) [40] offering measures of behaviours aligned to DSM-5 domains of Depression, Anxiety, Autism Spectrum, ADHD, and Oppositional Defiance (t-scores; population *M* = 50, *SD* = 10); and the MacArthur-Bates Communicative Development Inventories (MCDI) [41] offering receptive and expressive vocabulary counts and total gesture score.

The autistic children completed two additional direct assessments. The Autism Diagnostic Observation Schedule-2nd Edition (ADOS-2) [24] quantifies Social Affect and Restricted Repetitive Behaviour difficulties. Calibrated Severity Scores (CSS; range 1–10) were derived for comparability across Toddler Module and Modules 1–3, and evidenced very good within-trial inter-rater agreement (intraclass correlation = 0.84 on *n* = 15 double-coded assessments). The Mullen Scales of Early Learning (MSEL) [42] summarises developmental/cognitive abilities across Visual Reception and Fine Motor (non-verbal domain) and Receptive and Expressive Language (verbal domain) scales, within an Early Learning Composite (ELC) SS. From these scale scores, we computed Non-Verbal-, and Verbal- Developmental Quotients (DQ) for analysis (i.e., relevant scale Age-Equivalence [AE] scores/child chronological age * 100).

### Data collection and analysis procedure

The prospectively registered clinical trial protocol (written by KH, CB, LC, AJOW) specified recruitment and data collection procedures carried out by clinical researchers (KH, LC, DT, AC, CB, CG, JS; see Additional File). Algorithm development analysis work was not pre-specified but, rather, was conducted after all trial data collection was complete by JKC staff (MN, AS, SH) working independently of the clinical team. Following finalisation of the single ‘best-fit’ *Gazefinder* autism Classification Algorithm, clinical researchers undertook post-hoc exploratory mis/classification accuracy analysis on demographic and clinical/behavioural data.

Algorithm development was conducted following a previously-reported method [27], using Stata v15.1 and R v3.6.2, with ROC-kit 0.91 for resampling to compute 95% confidence intervals (95%CIs) for AUC values. As detailed in the online Additional File (including Tables AF1, AF2 and Figure AF3), gaze fixation durations were determined, sampling corneal reflection to X, Y coordinates on the monitor at 50 Hz. Across the ‘Scene 10A’ sequence, gaze fixation durations were computed across 99 single- and 8 paired ROIs (as a proportion of fixation to the given ROI/s divided by the duration of stimulus presentation for each trial, for a score between 0.0 and 1.0). This was repeated for proportionate gaze fixation durations during (a) the first 1 s, and (b) the first 2 s only of each trial, resulting in a final set of 321 candidate datapoints derived for each participant, for potential inclusion in the Classification Algorithm (see Table AF8).

Candidate datapoints found to be correlated >|0.25| with case/control group membership (identified in Table AF8) were retained for further consideration. To avoid redundancy and potential overfitting, datapoints were iteratively excluded until the ROI combination with best classification performance was identified. Fit was tested using Receiver Operating Characteristic (ROC) AUC analysis, yielding sensitivity and specificity parameters for a threshold score set to maximise accuracy (following the Youden J index) [43]. The final *Gazefinder* autism Classification Algorithm was standardised for values between 0 and 100. Leave-one-out cross validation (LOOCV) was used to appraise potential overfit [44].

## Results

### Participant characterisation

**Fig. 2:**
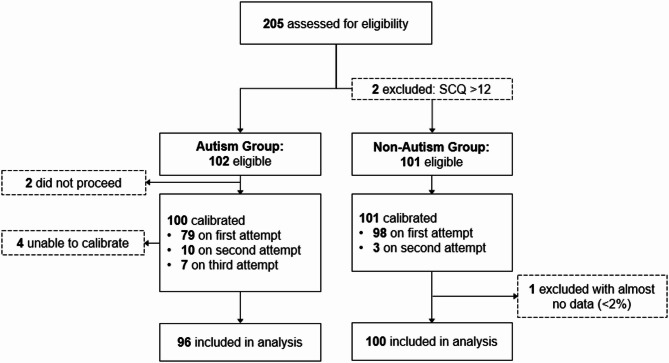
Participant flow through the trial, from recruitment and eligibility checks through attempted and successful *Gazefinder* autism eye-tracking assessment

Among 205 children recruited into the trial were 102 potential ‘cases’—children with community autism diagnoses—and 103 potential ‘controls’—children with no parent-reported developmental concerns or autism among first-degree relatives. Two potential participants in the latter group returned SCQ Total Scores > 12 and so were withdrawn from the cohort for a final eligible sample of 203; 102 autistic and 101 non-autistic children (see Fig. 2). The *Gazefinder* autism eye-tracking assessment was completed with 96 of 102 autistic children, with missing data for 6 in this group including 2 due to adverse responses during other assessments (resulting in decision to discontinue testing) and 4 due to repeated unsuccessful *Gazefinder* calibration attempts (also leading to discontinue decision). While all 101 eligible non-autistic children completed *Gazefinder* autism assessment, one subsequently returned almost no tracking data (< 2%) resulting in final usable data for 100 in this group.

Table 1 summarises the socio-demographic characteristics of 196 autistic and non-autistic participants with any analysable *Gazefinder* autism assessment data (see Additional File Tables AF4 & 5 showing similar characteristics for all 203 recruited eligible participants). Briefly, children in the Autism Group included greater proportion of males, were older on average, and reflected greater socio-demographic diversity than those in the Non-Autism Group. Clinical/behavioural phenotyping data (summarised in Table 2) confirmed the anticipated heterogeneity within the Autism Group, and the range of scores broadly within the typically range for children in the Non-Autism Group.

**Table 1:** Personal and familial socio-demographic characteristics of children with *Gazefinder* autism assessment data included in algorithm development

		Autism Group **(n = 96)**	Non-Autism Group (n = 100)	Between-Group Difference
Age in months: Mean (SD) range		44 (8.8) 24–60	40 (10.6) 24–60	*U* = 5825.50, *p* <.05, *r*_*rb*_=.21
Sex at Birth: n Male/Female		78/18	58/42	*χ*^*2*^(1) = 12.47, *p* <.001
Gestational Age at Birth	Full Term	82	92	*χ*^2^(1) = 0.22, *p* =.640^a^
	32–37 weeks	6	6	
	< 32 weeks	1	0	
	Missing/Not Disclosed	7	2	
Birth Order among Siblings	Only Child	24	32	*χ*^2^(2) = 1.09, *p* =.581
	First Born	29	26	
	Later Born	37	41	
	Missing/Not Disclosed	6	1	
Ethnicity	Caucasian Australian	55	84	*χ*^2^(1) = 12.48, *p* <.001^a^
	Indigenous Australian	3	0	
	North-East Asian	3	2	
	South-East Asian	3	0	
	Mixed Ethnicity	14	11	
	Other Ethnicity	9	1	
	Missing/Not Disclosed	9	2	
Family Composition	Dual Parent	75	97	*χ*^2^(1) = 12.36, *p <*.001^a^
	Single Parent	12	2	
	Other Adults	3	0	
	Missing/Not Disclosed	6	1	
Nominated Primary Carer	Mother	84	92	*χ*^2^(1) = 0.18, *p* =.670^a^
	Father	3	7	
	Grandparent	3	0	
	Missing/Not Disclosed	6	1	
Primary Carer Education	Primary School	1	0	*χ*^2^(2) = 20.64, *p* <.001^b^
	Secondary (15-16y)	11	2	
	Secondary (17-18y)	10	3	
	Degree	32	28	
	Post-Graduate	34	66	
	Missing/Not Disclosed	8	1	
Primary Home Language	English	83	97	*χ*^2^(1) = 3.45, *p* =.063
	Other	7	2	
	Missing/Not Disclosed	6	1	
Household Income	<AUD$25,000	6	0	*χ*^2^(4) = 24.58, *p <.*001
	AUD$25–50,000	11	3	
	AUD$50–85,000	11	5	
	AUD$85–115,000	19	24	
	>AUD$115,000	29	64	
	Missing/Not Disclosed	20	4	

Note. ^a^ Two-category comparisons (full term vs. any pre-term birth; Caucasian Australian vs. any other ethnicity group; dual parent vs. single parent/other adults; mother vs. father). ^b^ Three-category comparison (any primary/secondary vs. degree vs. post-graduate education)

**Table 2:** Clinical phenotyping scores for children with *Gazefinder* autism assessment data included in algorithm development

	Autism Group		Non-Autism Group		Between-Group Difference
	*n*	M (SD) range	*n*	M (SD) range	
SCQ Total Score	88	19.26 (6.11) 5–31	100	3.56 (2.51) 0–10	*U* = 8715.50, *p* <.001, *r*_*rb*_= 0.98
VABS ABC SS	87	76.0 (12.1) 50–108	93	111.2 (13.5) 80–139	*t*(178) = -18.4, *p* <.001, *d *= -2.47
CBCL T-Score	80		97		
Depression		64.74 (10.47) 50–95		52.97 (4.69) 50–70	*U* = 6813.0, *p* <.001, *r*_*rb*_= 0.76
Anxiety		60.94 (11.11) 50–90		52.07 (4.76) 50–85	*U* = 6000.0, *p* <.001, *r*_*rb*_= 0.55
Autism Spectrum		74.03 (8.76) 50–94		51.27 (2.70) 50–64	*U* = 7638.5, *p* <.001, *r*_*rb*_= 0.97
ADHD		61.51 (8.29) 50–76		52.00 (4.29) 50–76	*U* = 6691.0, *p* <.001, *r*_*rb*_= 0.72
Oppositionality		59.66 (9.19) 50–80		52.50 (4.42) 50–73	*U* = 5871.0, *p* <.001, *r*_*rb*_= 0.51
ADOS-2	94				
Overall CSS		7.53 (1.60) 3–10	-	-	-
Social Affect		12.15 (3.94) 3–20	-	-	-
Restricted/Repetitive		4.98 (1.83) 1–8	-	-	-
MSEL	96				
ELC SS		66.42 (21.25) 49–134	-	-	-
Non-Verbal DQ		70.10 (21.36) 27–121	-	-	-
Verbal DQ		61.31 (29.93) 13–137	-	-	-
MCDI	96				
Receptive Vocabulary		354.1 (209.0) 6–678	-	-	-
Expressive Vocabulary		284.1 (229.1) 0–678	-	-	-
Total Gestures		39.69 (14.81) 0–61	-	-	-

Note. SCQ = Social Communication Questionnaire [33]; VABS ABC SS = Vineland Adaptive Behavior Scales-2nd edition [39] Adaptive Behaviour Composite Standard Score; CBCL = Child Behaviour Checklist [40]; ADOS-2 CSS = Autism Diagnostic Observation Schedule– 2nd Edition [24] Calibrated Severity Score; MSEL = Mullen Scales of Early Learning [42]; ELC SS = Early Learning Composite Standard Score; DQ = Developmental Quotient; MCDI = McArthur-Bates Communicative Development Inventories [41]

### Outcome data

*Overall tracking and behaviour during assessment.* Among the 196 children who completed the *Gazefinder* autism assessment, there was a statistically significant between-group difference in overall tracking rate, with large effect size (*U* = 2535.50, *p* <.001, *r*_*rb*_= 0.08), with scores highly variable within each group but favouring the Non-Autism (*M* = 89.4, *SD* = 12.0; range 31.3–99.6) over the Autism Group (*M* = 76.0, *SD* = 19.8; range 14.0–99.0). Assessor notes regarding child behaviour during *Gazefinder* autism assessment suggested that low tracking rates likely reflected child difficulty maintaining attention to screen, frequent movement during stimulus presentation (such that gaze detection was obscured), emotion regulation difficulties, and/or noncompliance/negative response to testing. Recorded overall tracking rates for those children with assessor-noted behavioural difficulties were substantially lower than for those with no such notes made (see Additional File, including Figure AF7). As detailed in Additional Material Table AF9, within neither group was there any significant association of overall tracking rate with child age, sex, site, or other parent-report measures. Among the Autism Group only, overall tracking rate was weakly negatively correlated with primary caregiver education level, and positively so with overall developmental/cognitive ability, VDQ and NVD, but unrelated to other direct-assessment measures of clinical-behavioural phenotype (i.e., ADOS-2, MSEL).

*Calibration attempts and sustained tracking across the stimulus sequence.* As shown in Fig. 2 (above), most children in each group were successfully calibrated on the first attempt, with small numbers successfully calibrated on second or third attempts. Autistic children requiring three calibration attempts had lower language production and comprehension scores (per MCDI) than those requiring two attempts (with intermediate but non-significantly different scores for those calibrated at first attempt). Calibration attempt frequency was also associated with overall tracking rate (i.e., autistic children successfully calibrated on first/second attempt returning higher overall tracking rates than those requiring a third attempt; see Additional File), but not with any other clinical/behavioural score.

**Fig. 3:**
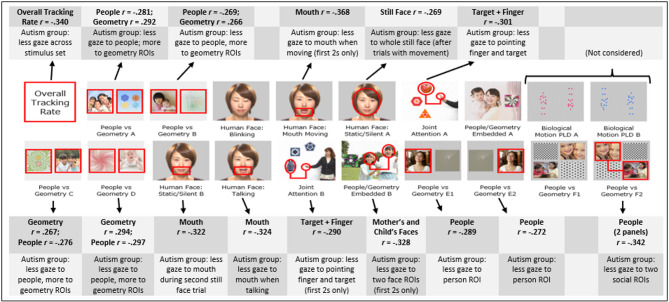
Summary of ROIs from *Gazefinder* autism assessment ‘Scene 10A’ trials retained within best-fit Classification Algorithm (red square- and circular- ROIs as relevant), including magnitude and direction of associations of fixation data to individual ROIs, with child group (coded Autism = 1, Non-Aautism = 0). Note. Other ROIs pre-specified for data capture but not retained in the final algorithm are omitted here (but shown in Additional File Figure AF6)

*Algorithm performance.* Figure 3 summarises key stimuli within the *Gazefinder* autism assessment ‘Scene 10A’, highlighting those ROIs retained for the final Classification Algorithm and, for each of these, the correlation coefficient reflecting association of gaze fixation data with child group (coded Autism = 1, Non-Autism = 0) and brief interpretative description of each effect.

**Fig. 4:**
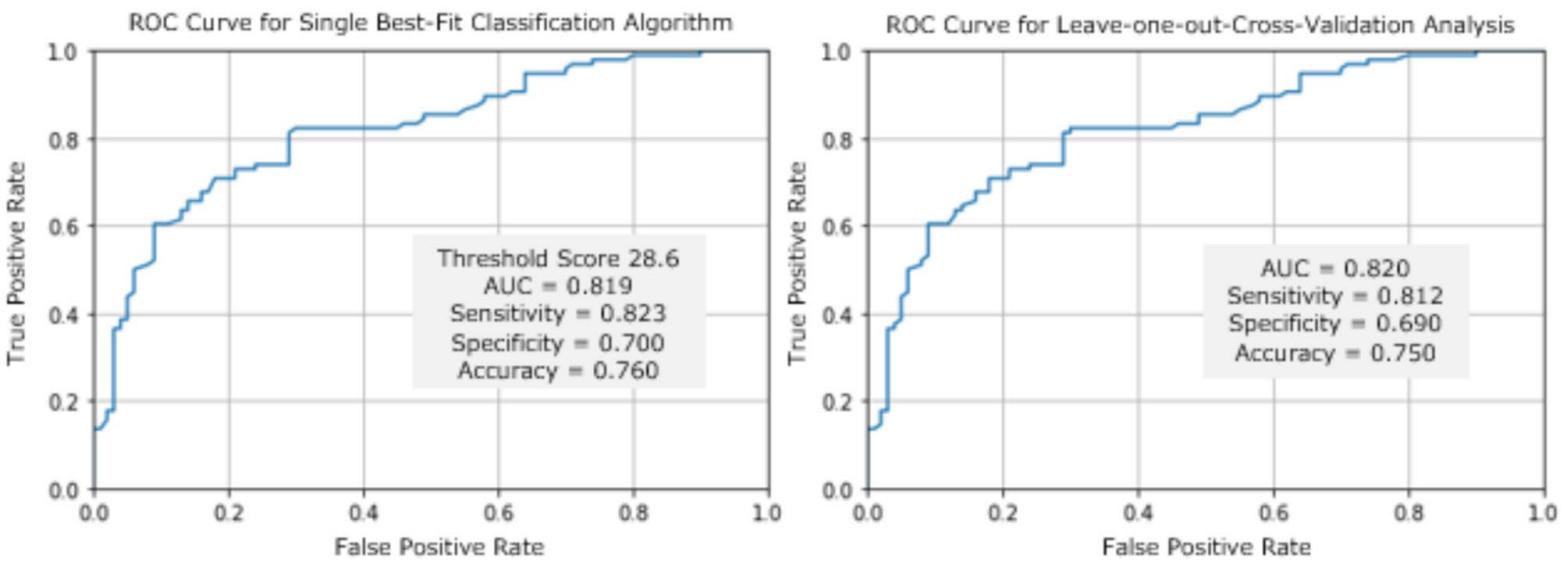
ROC AUC results for best-fit algorithm and following leave-one-out-cross-validation, for autism Classification Algorithm derived from gaze fixation data to ROIs in Fig. 3

Figure 4 shows that the best-fit autism Classification Algorithm (singular score computed to range plausibly between 0 and 100; with classification threshold = 28.6) had AUC = 0.82, with 82% sensitivity, 70% specificity, and 76% overall accuracy for case/control classification. LOOCV analysis, also shown in Fig. 4, returned similar results—AUC = 0.82 (threshold score range 26.6–30.6 [IQR 28.6–28.8]; for 81% sensitivity, 69% specificity, 75% overall accuracy—suggesting good model fit to the data.

The Classification Algorithm correctly classified 79 ‘cases’ of autistic children and 70 non-autistic ‘controls’, misclassifying 30 non-autistic children as ‘cases’ (false positives) and 17 autistic children as ‘controls’ (false negatives). Post-hoc analysis (Table 3) showed that false positives differed from true negatives on overall tracking rate and age, while false negatives also differed from true positives on overall tracking rate, as well as on developmental/cognitive abilities and parent-reported oppositionality (moderate-to-large effects), with a non-significant trend toward differences on two behavioural autism measures (moderate effects). There was little suggestion that classification accuracy varied by socio-demographic characteristics (Additional File Table AF11), although algorithm performance properties were reduced for some socio-demographic subgroups (but similar, or even improved, for others; detailed in Table AF12).

**Table 3:** Comparison of correctly and mis-classified autistic and non-autistic children on range of available characterisation measures

	Autism ‘Cases’		Between-Group Difference	Non-Autistic ‘Controls’		Between-Group Difference
	False Negative (*n* = 17)	True Positive (*n* = 79)		False Positive (*n* = 30)	True Negative (*n* = 70)	
Site: n Melbourne/Perth/Tasmania	7/6/4	32/28/19	*χ*^*2*^(2) = 0.00, *p* =.998	8/21/1	29/39/2	*χ*^*2*^(2) = 1.97, *p* =.374
Sex at Birth: n Male/Female	12/5	66/13	*χ*^*2*^(1) = 1.54, *p* =.214	19/11	39/31	*χ*^*2*^(1) = 0.50, *p* =.479
Age M(SD) in months	42.59 (8.47)	44.17 (8.90)	*t*(94) = 0.67, *p* =.506, *d* = 0.18	43.30 (11.51)	38.63 (9.93)	*t*(98) = -2.05, *p* =.043 *d *= -0.45
Prematurity: n Term/<37wks	17/0	65/7	*χ*^*2*^(1) = 1.79, *p* =.180	27/3	65/3	*χ*^*2*^(1) = 1.13, *p* =.288
Calibration Attempts: n 1/2+	15/2	64/15	*χ*^*2*^(1) = 0.50, *p* =.479	30/0	67/3	*χ*^*2*^(1) = 1.33, *p* =.250
Gaze Rate (overall % tracking)	92 (5.1)	73 (20.1)	*U* = 220.00, *p <*.001, *r*_*rb*_= -0.67	81 (16.0)	93 (7.4)	*U* = 1628.50, *p <*.001, *r*_*rb*_= 0.55
SCQ Total Score	16.77 (5.44)	19.86 (6.15)	*t*(86) = 1.90, *p =*.060, *d* = 0.51	3.83 (2.45)	3.44 (2.54)	*t*(98) = -0.71, *p =*.478, *d *= -0.16
VABS ABC SS	79.35 (10.93)	75.19 (12.29)	*t*(85) = -1.28, *p =*.204, *d *=-0.35	111.75 (14.13)	111.02 (13.38)	*t*(91) = -0.24, *p =*.812, *d *= -0.05
CBCL Depression	64.06 (10.28)	64.91 (10.59)	*t*(78) = 0.29, *p =*.775, *d* = 0.08	52.17 (3.48)	53.31 (5.11)	*t*(95) = 1.09, *p =*.277, *d* = 0.24
CBCL Anxiety	64.38 (12.25)	60.08 (10.73)	*t*(78) = -1.39, *p =*.168, *d *= -0.39	51.90 (3.51)	52.15 (5.23)	*t*(95) = 0.24, *p =*.814, *d* = 0.05
CBCL Autism Spectrum	72.63 (9.14)	74.38 (8.70)	*t*(78) = 0.71, *p =*.478, *d *= -0.20	51.07 (2.19)	51.35 (2.91)	*t*(95) = 0.47, *p =*.638, *d* = 0.11
CBCL ADHD	62.81 (9.03)	61.19 (8.14)	*t*(78) = 0.70, *p =*.487, *d* = 0.20	50.93 (2.02)	52.46 (4.89)	*t*(95) = 1.62, *p =*.109, *d* = 0.36
CBCL Oppositionality	64.50 (10.39)	58.45 (8.54)	*t*(78) = -2.43, *p* =.018, *d *= -0.68	51.86 (3.90)	52.77 (4.62)	*t*(95) = 0.92, *p =*.359, *d* = 0.20
MCDI Receptive Vocabulary	429.21 (198.1)	338.13 (209.2)	*t*(74) = -1.49, *p =*.142, *d *= -0.44	-	-	-
MCDI Expressive Vocabulary	352.3 (212.5)	268.71 (231.5)	*t*(74) = -1.24, *p =*.220, *d *= -0.37	-	-	-
MCDI Total Gestures Score	42.50 (10.18)	39.06 (15.65)	*t*(75) = -0.78, *p =*.436, *d *= -0.23	-	-	-
MSEL ELC SS	81.93 (22.49)	63.44 (19.79)	*t*(91) = -3.24, *p* =.002, *d *= -0.91	-	-	-
MSEL Non-Verbal DQ	84.89 (18.85)	66.91 (20.61)	*t*(94) = -3.31, *p* = 001, *d *= -0.89	-	-	-
MSEL Verbal DQ	82.58 (21.73)	57.27 (29.66)	*U* = 291.00, *p* =.002, *r*_*rb*_= -0.51	-	-	-
ADOS Overall CSS	7.06 (2.16)	7.63 (1.45)	*U* = 757.50, *p =*.353, *r*_*rb*_= 0.14	-	-	-
ADOS Social Affect Algorithm	10.47 (4.22)	12.51 (3.81)	*t*(93) = 1.97, *p =*.052, *d* = 0.53	-	-	-
ADOS RRB Algorithm	4.35 (1.54)	5.12 (1.87)	*t*(93) = 1.57, *p =*.120, *d* = 0.42	-	-	-

Note. Data are Mean (StDev) unless otherwise specified. SCQ = Social Communication Questionnaire [33]; VABS ABC SS = Vineland Adaptive Behavior Scales-2nd edition [39] Adaptive Behaviour Composite Standard Score; CBCL = Child Behaviour Checklist [40]; MCDI = McArthur-Bates Communicative Development Inventories [41]; MSEL = Mullen Scales of Early Learning [42]; ELC SS = Early Learning Composite Standard Score; DQ = Developmental Quotient; ADOS-2 CSS = Autism Diagnostic Observation Schedule– 2nd Edition [24] Calibrated Severity Score; RRB = Restricted Repetitive Behaviours

## Discussion

We tested potential for the brief, standardised *Gazefinder* autism eye-tracking assessment to support classification during the developmental period of early childhood when diagnostic assessment often occurs. The large majority of a sample of 203 recruited, eligible autistic and non-autistic children—aged 2 years 0 months to 4 years 11 months—were successfully calibrated and thereafter remained reasonably attentive and engaged during the ~ 2.5-minute stimulus presentation. Very few children showed any adverse emotional or behavioural responses (whether specifically during *Gazefinder* eye-tracking, or other assessment activities). A single best-fit *Gazefinder* autism Classification Algorithm, derived from gaze fixation duration data, showed ‘good’ overall accuracy for differentiating children in the Autism and Non-Autism Groups. Specifically, with a plausible range between 0 and 100, and threshold score of 28.6, the algorithm showed 82% sensitivity, 70% specificity, 76% overall accuracy, and AUC = 0.82, correctly classifying 79/96 autistic and 70/100 non-autistic children.

### Scene elements supporting group differentiation

Pre-specified ROIs within the ‘Scene 10A’ autism assessment reflected preferential attention to paired social vs. non-social scenes (i.e., people vs. geometry), to human face and features (e.g., eyes vs. mouth) within static and dynamic trials, and to biological motion PLD-type animations, and to referent and target item (vs. distractors) within referential attention trials. Those ROIs retained within the final Classification Algorithm—developed for ‘best-fit’ with the data, and checked via LOOCV procedure—reflected gaze fixation patterns broadly consistent with findings from other autism eye-tracking studies and clinical behavioural observation. Specifically, consistent with previous preferential attention findings [13, 21, 45], the Autism Group showed *increased* gaze to non-social- and *reduced* gaze to social elements within several paired presentations of people vs. geometry, and reduced gaze to the human face during first static presentation. Within a second static human face trial and two dynamic trials with mouth moving (silently and while talking), the Autism Group had reduced gaze to the mouth ROI. Reduced gaze by the Autism Group toward key referential attention trial elements (i.e., pointing hand/figure and target object) was also seen, consistent with clinical accounts of joint-attention differences [1, 46].

Interestingly, there was no particular association of Autism Group membership with reduced gaze toward *eye* ROIs of the human face, as plausibly anticipated from other eye-tracking work [13] and clinical accounts of reduced eye-contact [1]. While retained in the ‘Scene 10A’ presentation, ROIs for the two biological motion PLD-type trials were not considered during algorithm development given mixed evidence of autism-related viewing differences [16, 35–37]. An additional parameter included in the ‘best-fit’ Classification Algorithm was the overall tracking rate—the duration of all gaze fixations captured across any part of the ‘Scene 10A’ presentation—itself moderately associated with child group. Overall tracking rate plausibly reflects domain-general sustained attention capacity [47, 48], consistent with behavioural notes made by examiners during *Gazefinder* autism assessment (for children in either groups), and the observed association between overall tracking rate and standardised developmental/cognitive assessment scores for the Autism Group.

### Insights from exploratory mis/classification analysis

While feasibly administered, generally well tolerated, and evidencing ‘good’ overall classification accuracy according to our pre-specified interpretative criterion, sizeable subsets of children in each group were ultimately misclassified—17 autistic and 30 non-autistic children. Post-hoc testing suggested that, compared to true negative ‘controls’, non-autistic children misclassified as autistic were older and returned lower overall tracking (although not as low as for *true positive* ‘cases’). Compared to true positive ‘cases’, misclassified autistic children returned higher overall tracking (similar to rates for *true-negative* ‘controls’) and had clinical/behavioural test scores suggesting less pronounced behavioural autism features and lesser developmental/cognitive disability.

*Gazefinder* technology was developed to aide autism identification and diagnosis—not to replace thorough developmental, behavioural assessment, and clinical judgement, but to support the process through standardisation, and informative objective data. The performance profile reported here, favouring false positive- over false negative misclassification, suggests the best contribution, however, may be interim to screening at scale and selective diagnostic assessment. For an evidenced positioning of appropriate utility, future research regarding *Gazefinder* autism assessment could examine its value-add alongside or subsequent to existing screening tools (e.g., the M-CHAT [49] or SACS [50]), with participant recruitment mirroring a clinical referral pathway *prior to* diagnostic assessment (i.e., requiring more specialist clinical skills, including ADOS [51] administration). The profile of currently misclassified autistic children—with similar overall tracking rate to non-autistic ‘controls’, and subtler autism presentation than their correctly classified counterparts—suggests this technology may usefully efficiently corroborate a proposed autism diagnosis among young children whose presentation is fairly clear, expediting their access to targeted supports whilst also conserving other limited resources for referrals where establishing emergent autism/other neurodivergence requires closer clinical attention. Variation in classification algorithm performance properties when applied to particular socio-demographic subgroups, suggests there would be benefit in further examining children from minority subgroups (but noting performance properties were similar to the average, or even stronger, for some subgroups, and that small subsample sizes prevent strong conclusions here).

### Limitations and future directions

Potential limitations of this work include our handling of the overall tracking rate, which differed significantly between the Autism and Non-Autism Groups, and between correctly vs. mis-classified Autism subgroups (but with no indication of bias in association with participant socio-demographic characteristics). Unlike others who often set minimum overall data capture thresholds for participant retention in analysis—rates ≥ 20% [52], or even ≥ 70% [26] or ≥ 80% [19]—we set no such limit on data sufficiency, seeking a conservative and robust test of *Gazefinder* utility within an ecologically valid clinical assessment type context (i.e., excluding only one non-autistic child who returned virtually no tracking data). We also included overall tracking rate in the ‘best-fit’ autism Classification Algorithm, contributing informatively as a potential proxy for sustained attention [47, 48] to group classification. Plausibly, however, setting some minimum data capture threshold might support more accurate performance of a classification algorithm based on ROI fixation duration data only, including reduced false negative rate. Future research with *Gazefinder* technology could directly consider how to balance rigour with ecological validity, and the construct validity of overall tracking rate as a relevant metric in addition to ROI-specific fixation durations.

Other potential limitations arise from our ‘off the shelf’ use of *Gazefinder* ‘Scene 10A’ autism assessment, developed independently from our team and used here without modification. The brief, engaging and low-demand stimulus sequence was designed to maximise likely successful data capture from viewers with varied clinical presentations and capacities, including infants and young children. However, fluctuating mood, motivation, alertness/fatigue etc. may influence engagement with even low-demand testing, and potentially particularly so given the brevity of this ‘Scene 10A’ autism eye-tracking assessment. There has been, to our knowledge, no direct testing of within-session or test-retest *Gazefinder* autism assessment reliability. Moreover, we note that some of our participants—mostly in the Autism Group—required more than one attempt at calibration. However, the large majority were ultimately able to be calibrated—including children with significant developmental and language challenges—and returned analysable gaze data thereafter. Hence, this study offers some reassurance on the feasibility of *Gazefinder* use for autism assessment with children with varied clinical/behavioural presentations. Future research should nevertheless consider refining protocols for achieving calibration, formalising within-assessment and test-retest reliability, and ensuring successful use across the full range of clinical autism presentation.

We acknowledge that the current algorithm development work was conducted by employees of the product manufacturer, and restricted to gaze fixation durations (i.e., vs. other possibly informative metrics such as gaze latencies, saccades, etc.). Whilst not blinded to participant grouping, algorithm development technicians were otherwise completely independent of study conduct, including trial design and preregistration, and participant recruitment and data collection, and the focus on gaze fixation durations was consistent with other independent work on this technology [19, 26, 27]. Future research—drawing on and extending beyond the current dataset—could consider more diverse and dynamic gaze parameters, and the potential for stimulus features and sequencing to impact participants’ gaze behaviour and the data thereby returned. Such efforts could increase understanding of both the nature of visual attention differences in early childhood autism and, if greater accuracy can be achieved, the clinical potential of this brief, standardised, objective tool.

Study design may also have had limiting impacts here, including that our recruitment approach yielded groups that were not matched in terms of child sex ratio, age, and several other socio-demographic characteristics, nor necessarily representative of the local population. Given known phenotypic heterogeneity in autism—which we sought to capture, whereas others’ approach is often to seek to constrain [27]—and anticipating the challenges inherent to achieving representative and closely matched groups, our pre-specified plan was to intentionally recruit well beyond the required sample size for a well-powered primary analysis, and also ensuring sufficiency for exploratory mis/classification analysis. Again, it seems unlikely that lack of group matching has critically impacted our study, given no substantive association of socio-demographic characteristics with participants’ *Gazefinder* autism Classification Algorithm scores, nor with correct/mis-classification status, and (to the extent our subsample sizes allow for inference) no robust pattern of classification underperformance among particular socio-demographic subgroups. Future research could nevertheless consider how the current operationalisation of constructs (i.e., properties of stimuli, ROIs, and visual attention metrics), and ultimate algorithm performance may generalise beyond features of the current participant sample, including to viewers from other geographic regions and socio-cultural/ethnic backgrounds. More comprehensive characterisation of ‘case’ and ‘control’ groups alike would allow for more robust verification of technology fitness-for-purpose, and offer potential insights into factors driving misclassification where this occurs.

Finally, while our use of statistical LOOVC procedure with data generated from this *development* sample gives preliminary confidence that the *Gazefinder* autism Classification Algorithm is not overfitted to the current dataset, a key future need is for replication with data from an *independent validation* sample. We note similar performance metrics for the current autism Classification Algorithm for 2-to 4-year-olds as reported independently for *Gazefinder* assessment and algorithm development work with participants at other ages/developmental stages—in later childhood beyond age 5 years [27] and among adolescents/adults [19]—and for classification performance of the established ADOS-2 behavioural autism assessment [24]. Moreover, and as described above, future investigation of the utility of *Gazefinder* autism assessment should move beyond ‘case-control’ study design and the comparison of a-priori determined Autism and Non-Autism Groups, to further improve ecological validity; increasing within-sample heterogeneity and the potential for multiple clinical outcome situations. This could be achieved through the adoption of ‘infant sibling’ study methodology, with many examples of teams doing so with other eye-tracking technologies (e.g., 23, 38, 45). Better still, if perhaps more resource intensive, positioning *Gazefinder* autism assessment research within a clinical or community ascertainment pathway—as in our own recently-completed proof-of-concept work with a referred infant sample [30]—could offer a highly ecologically-valid test of the utility for early screening and/or diagnosis of this technology, and further insights into the mechanistic underpinnings of visual attention differences in autism and other neurodivergence, as they emerge across the early years.

## Electronic supplementary material

Below is the link to the electronic supplementary material.

Supplementary Material 1

## Data Availability

The dataset supporting the conclusions of this article is available on reasonable request by email directed to the corresponding author and subject to agreement of the funder/product developer.

## Footnotes

1 Noting the implied pathology carried by terms such as ‘cases’ and ‘controls’, we have sought to reserve use of these for reference to specific clinical trial design features, and otherwise refer to people (whether individuals or as groups) using a combination of person-first language and the identity-first language advocated by many autistic people (32).

## Abbreviations

ABC: Adaptive Behaviour Composite
ACTRN: Australian New Zealand Clinical Trials Registry Number
ADHD: Attention Deficit Hyperactivity Disorder
ADI-R: Autism Diagnostic Interview – Revised
ADOS: Autism Diagnostic Observation Schedule
AE: Age Equivalent
AF: Additional File
AUC: Area Under the Curve
CBCL: Child Behaviour Checklist
CSS: Calibrated Severity Score
DSM-5: Diagnostic and Statistical Manual of Mental Disorders– 5th Edition
DQ: Developmental Quotient
ELC: Early Learning Composite
HEC: Human Research Ethics Committee
IQR: Inter Quartile Range
JKC: JVCKENWOOD Corporation
LOOCV: Leave-one-out Cross Validation
LTU: La Trobe University
MCDI: MacArthur Bates Communicative Development Inventories
MSEL: Mullen Scales of Early Learning
ROC: Receiver Operating Characteristics
ROI: Region of Interest
SCQ: Social Communication Questionnaire
SS: Standard Score
VABS: Vineland Adaptive Behaviour Scales
