# Supplementary material for: Accuracy of a 2-minute eye-tracking assessment to differentiate young children with and without autism
Source: Mol Autism. 2025 Jul 10;16:36. doi: 10.1186/s13229-025-00670-4 (PMC12247230; doi:10.1186/s13229-025-00670-4)
Supplement: Supplementary file 1 — Supplementary Material 1 [file 13229_2025_670_MOESM1_ESM.docx]

**ADDITIONAL FILE TO SUPPLEMENT**

Accuracy of 2-minute eye-tracking assessment to differentiate young children with and without autism

Kristelle Hudry, PhD, Lacey Chetcuti, PhD, Diana Weiting Tan, PhD, Alena Clark, BHSc, Alexandra Aulich, BAa (Hons), Catherine A. Bent, PhD, Cherie C. Green, PhD, Jodie Smith, PhD, Kathryn Fordyce, BSpPath (Hons), Masaru Ninomiya, BEng, Atsushi Saito, MEng, Shuji Hakoshima, BEng, Andrew J.O. Whitehouse, PhD

Drs. Hudry, Bent, Green and Smith and Ms Aulich are with the Department of Psychology, Counselling and Therapy, School of Psychology and Public Health, La Trobe University, Melbourne, Australia. Dr Smith is also with the Speech Pathology Division, School of Allied Health, Human Services and Sport, La Trobe University, Melbourne Australia. Dr Chetcuti was with the Department of Psychology, Counselling and Therapy, School of Psychology and Public Health, La Trobe University, Australia at the time this work was conducted, and is now with the Department of Psychiatry and Behavioral Sciences, Stanford University, California USA. Drs Tan and Whitehouse and Ms Clark are with The Kids Institute, University of Western Australia, Perth, Australia. Dr Tan is also with the School of Psychological Science, University of Western Australia, Perth, Australia and the Macquarie School of Education, Macquarie University, Sydney, Australia. Ms Fordyce is with St Giles Society Limited, Burnie, Australia. Mr Ninomiya, Saito and Hakoshima are with JVCKENWOOD Corporation, Yokohama-shi, Kanagawa, Japan.

**ADDITIONAL MATERIAL CONTENTS**

| **Section** | **Page** |
| --- | --- |
| Original Trial Protocol | 3 |
| Final Trial Protocol (including list of revision chronology) | 23 |
| Additional Detail on Methods: | 51 |
| Details of *Gazefinder* Apparatus and Assessment | 51 |
| *Gazefinder* apparatus description and specifications (incl. Figures AF1, AF2) | 51 |
| Determination of gaze fixations and movement saccades (incl. Figure AF3) | 52 |
| Set-up, positioning check, and five-point calibration procedure (incl. Figures AF4, AF5) | 53 |
| Summary of ‘Scene 10A’ trials and pre-specified ROIs (incl. Figure AF6) | 54 |
| Table AF1. Detailed specifications of key stimuli within ‘Scene 10A’ | 55 |
| Detailed ROI parameters (incl. Figure AF7) | 57 |
| Table AF2. Detailed specifications of ‘Scene 10A’ ROIs retained for classific. algorithm | 58 |
| Details of Clinical Characterisation Measures | 59 |
| Additional Detail on Statistical Method: | 61 |
| Algorithm Development Analysis | 61 |
| Table AF3. Description of parameters within equation for *Gazefinder* classification algorithm | 61 |
| STROBE Reporting Checklist for Case-Control Studies | 62 |
| Additional Results | 64 |
| Detailed Sample Characterisation | 64 |
| Table AF4. Personal and familial socio-demographic characteristics of eligible children | 64 |
| Table AF5. Available data on clinical phenotyping measures for all eligible children | 65 |
| Within-group heterogeneity and between-group comparability | 66 |
| Table AF6. Clinical phenotyping data for final participant sample of children, by site | 69 |
| Details of *Gazefinder* Assessment Feasibility | 70 |
| Attempts at *Gazefinder* assessment and calibration | 70 |
| Adverse events and other behaviours of note during *Gazefinder* assessment | 71 |
| Table AF7. Detailed record of adverse events during and/or sustained post assessment | 72 |
| Variation in overall tracking rates and association with other characteristics (Fig. AF8) | 73 |
| Details of *Gazefinder* Classification Algorithm | 74 |
| Delineation of candidate Regions of Interest | 74 |
| Table AF8. Association of gaze fixations to all possible candidate ROIs with child group | 75 |
| Summary of final set of ROIs retained for Gazefinder classification algorithm (Fig. AF9) | 77 |
| Table AF9. Associations of tracking rate and algorithm score with child characteristics | 78 |
| Performance Properties of Gazefinder Algorithm vs. Other Diagnostic Tools (Table AF10) | 79 |
| Table AF11. Associations of range of socio-demographic characteristics with correct and mis-classification status for autistic ‘cases’ and non-autistic ‘controls’ | 80 |
| Table AF12. Summary of Gazefinder autism Classification Algorithm performance, overall and for participant subgroups | 81 |
| References | 82 |

# **ORIGINAL TRIAL PROTOCOL**


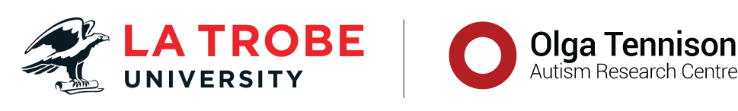


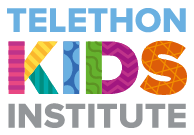


**Autism Research Team**

| protocol |
| --- |
| Clinical Trial of Gazefinder with Young Children with Autism and Typical Development |
| Protocol Number: TBC  Version: Pre-Registration DRAFT  Date: 22/01/2019 |
|  |
|  |
| **CONFIDENTIAL**  This document is confidential and the property of La Trobe University. No part of it may be transmitted, reproduced, published, or used without prior written authorisation from the institution.  **Statement of Compliance**  This document is a protocol for a research project. This study will be conducted in compliance with all stipulation of this protocol, the conditions of the ethics committee approval, the NHMRC National Statement on ethical Conduct in Human Research (2007) and the Note for Guidance on Good Clinical Practice (CPMP/ICH-135/95). |

Table of Contents

[**Table of Contents 2**](#_Toc535589210)

[**Administrative Information 4**](#_Toc535589211)

[1. Title 4](#_Toc535589212)

[2. Trial Registration 4](#_Toc535589213)

[2a Registry 4](#_Toc535589214)

[2b data set 4](#_Toc535589215)

[3. Protocol Version 6](#_Toc535589216)

[4. Funding 6](#_Toc535589217)

[5. Roles and Responsibilities 6](#_Toc535589218)

[5a Contributorship 6](#_Toc535589219)

[5b Sponsor Contact Information 6](#_Toc535589220)

[5c Sponsor and Funder 7](#_Toc535589221)

[5d Committees 7](#_Toc535589222)

[**Introduction 7**](#_Toc535589223)

[6. Background and Rationale 7](#_Toc535589224)

[6a Choice of Comparators 8](#_Toc535589225)

[6b Explanation for Choice of Comparators 8](#_Toc535589226)

[7. Objectives 8](#_Toc535589227)

[8. Study Design 9](#_Toc535589228)

[**Methods: Participants, Interventions, Outcomes 9**](#_Toc535589229)

[9. Study Setting 9](#_Toc535589230)

[10. Eligibility Criteria 9](#_Toc535589231)

[11. Participant Timeline 10](#_Toc535589232)

[12. Sample Size 11](#_Toc535589233)

[13. Recruitment 12](#_Toc535589234)

[**Methods: Data Collection, Management, Analysis 12**](#_Toc535589235)

[14. Data Collection Methods 12](#_Toc535589236)

[15. Data Management 12](#_Toc535589237)

[16. Statistical Methods 14](#_Toc535589238)

[16a Outcome 14](#_Toc535589239)

[16b Additional Analyses 14](#_Toc535589240)

[16c Missing Data 14](#_Toc535589241)

[**Methods: Monitoring 15**](#_Toc535589242)

[17. Data Monitoring 15](#_Toc535589243)

[17a Data Monitoring Committee 15](#_Toc535589244)

[17b Interim Analysis 15](#_Toc535589245)

[18. Harms 15](#_Toc535589246)

[18a Risk of Psychological, Emotional or Social Harms 15](#_Toc535589247)

[18b Physical Risks and Safety 16](#_Toc535589248)

[19. Auditing 17](#_Toc535589249)

[**ethics and Dissemination 17**](#_Toc535589250)

[20. Research Ethics Approval 17](#_Toc535589251)

[21. Protocol Amendments 17](#_Toc535589252)

[22. Consent 17](#_Toc535589253)

[23. Confidentiality 17](#_Toc535589254)

[24. Declaration of Interests 18](#_Toc535589255)

[25. Access to Data 18](#_Toc535589256)

[26. Dissemination Policy 19](#_Toc535589257)

[26a Trial Results 19](#_Toc535589258)

[26b Authorship 19](#_Toc535589259)

[26c Reproducible Research 19](#_Toc535589260)

[**Appendices 20**](#_Toc535589261)

[27. Informed consent Materials 20](#_Toc535589262)

[28. References 20](#_Toc535589263)

Administrative Information

1. Title

Clinical Trial of Gazefinder with Young Children with Autism and Typical Development

1. Trial Registration

*2a Registry*

This trial will be registered with the Australian and New Zealand Clinical Trials Registry (ANZCTR) following approval from the La Trobe University Human Ethics Committee.

Trial Identifier: To be confirmed

*2b data set*

| **Data Category** | **Information** |
| --- | --- |
| Primary registry and study identifying number | Australian and New Zealand Clinical Trials Registry Identifier: To be confirmed |
| Date of registration in primary registry | Following ethics approval |
| Secondary identifying numbers | N/A |
| Source(s) of monetary/material support | JVCKENWOOD Corporation (JKC, Japan) |
| Primary sponsor | La Trobe University |
| Secondary sponsor(s) | N/A |
| Contact for public queries | Dr Kristelle Hudry |
| Contact for scientific queries | Dr Kristelle Hudry |
| Public title | Could "Gazefinder" Eye-Tracking Technology Support the Earlier Identification and Diagnosis of Autism in Young Children? |
| Scientific title | Clinical Trial examining the sensitivity and specificity of Gazefinder for identifying young children with autism |
| Countries of recruitment | Australia |
| Health condition(s) or problem(s) studied | The accurate identification of autism in young children |
| Intervention(s) | N/A |
| Key inclusion and exclusion criteria | **Children with autism:**  Inclusion: Child age between 2 years 0 months and 4 years 11 months; diagnosis of autism from a community professional.  Exclusion: Uncorrected visual/hearing impairment  **Typically developing (TD) children:**  Inclusion: Child age between 2 years 0 months and 4 years 11 months  Exclusion: Uncorrected visual/hearing; parent-reported family history of autism and/or presence of another developmental condition and/or moderate-level autism behaviours (TD group). |
| Study type | Observational  Primary purpose: Natural History / Screening Duration: Cross-sectional Selection: Defined Population Timing: Both prospective and retrospective Patient Registry: No |
| Recruitment Status | Not yet recruiting |
| Date of first enrolment | February 2019 |
| Date of last data collection | December 2020 |
| Target sample size | 200 |
| Primary outcome(s) | Accuracy of Gazefinder to differentiate autism from TD |
| Exploratory secondary outcomes | Influence of characteristics of individual children with autism – such as age, core and associated symptom presentation, cognitive ability, etc. – on the accuracy of Gazefinder to differentiate autism from TD |

1. Protocol Version

| **Issue Date:** | 22 Jan 2019 |
| --- | --- |
| **Protocol amendment number:** | Pre-Registration DRAFT |
| **Author(s):** | Hudry, K., Bent, C., Chetcuti, L., Whitehouse, A. |

Revision Chronology:

| **Date of change** | **Summary of changes** |
| --- | --- |
| 22 Jan 2019 | Original |
|  |  |

1. Funding

| JVCKENWOOD Corporation (JKC)  3-12 Moriya-cho,  Kanagawa-ku  Kokohama-shi  Kanagawa  221-0022 Japan | Gazefinder is manufactured by JKC, Japan, and supplied to two trial sites – La Trobe University (LTU) and Telethon Kids Institute (TKI) – for the purpose of conducting this trial. JKC is funding the running costs of the trial, including providing equipment and research staff salary costs. The management, analysis and reporting of the study will be conducted independently of JKC. |
| --- | --- |

1. Roles and Responsibilities

*5a Contributorship*

| **Author Name** | **Summary of contribution** |
| --- | --- |
| **Dr Kristelle Hudry**  La Trobe University | KH conceived of the study and co-led a feasibility pilot with AW which has informed the current trial, and will coordinate the project |
| **Dr Catherine Bent**  La Trobe University | CB contributed to study design and will be involved in data collection and analysis |
| **Ms Lacey Chetcuti,** La Trobe University | LC contributed data collection as part of the feasibility pilot, contributed to study design and will be involved in data collection and analysis |
| **Prof. Andrew Whitehouse** Telethon Kids Institute | AW conceived of the study, co-led a feasibility pilot with KH which has informed the current trial and will lead activities at the partner site (Telethon Kids Institute, TKI) |

*5b Sponsor Contact Information*

| **Study Sponsor** | La Trobe University |
| --- | --- |
| **Sponsor’s Reference number (if applicable)** | N/A |
| **Contact name** | Kristelle Hudry |
| **Address** | C/O La Trobe University, School of Psychology and Public Health, Bundoora, VIC 3083 |
| **Telephone** | 9479 5649 |
| **Email** | **xxxxxxxxxxxxxxxxxxxx** |

*5c Sponsor and Funder*

LTU is the study sponsor and will oversee the conduct of the trial, including study design, data collection, management, analysis and interpretation, writing of the report and publication of results in peer-reviewed publications. Telethon Kids Institute (TKI) is a partner site for data collection under a forthcoming clinical research agreement with LTU. JKC as the funder of the trial will receive a report and de-identified copy of the raw data following the completion of the trial, as well as interim reports on study progress. Both LTU/TKI and JKC may publish and/or present the results of the trial, providing that the publication does not disclose confidential or commercially sensitive information and is not misleading to the public.

*5d Committees*

**Principal Investigator and Research Staff**

The joint research team (LTU and TKI sites) will meet at least quarterly to review project activities (e.g., recruitment and data collection), to ensure the conduct of the project is on track, protocols are consistent across sites, and that any arising issues are promptly addressed. The research teams are responsible for participant recruitment and data collection at each site, and site leads are jointly responsible for the preparation of study reports.

**Steering Committee**

A Steering/Data and Safety Management Committee will be convened to review the progress and conduct of the study. The composition of this committee will be determined in the early stages of the project and members will meet twice per year to approve the trial protocol, review the progress and conduct of the study, and agree on any necessary protocol changes. This committee will also be responsible for coordinating data audits at each site (see below).

Introduction

1. Background and Rationale

Autism Spectrum Disorder (ASD; hereafter, *autism*) is a lifelong developmental condition affecting at least 1% of people (e.g., Baio et al., 2018), characterized by impairments in social interaction, verbal and nonverbal communication skills, alongside the presence of restricted/repetitive patterns of behaviour (APA, 2013). Core symptom presentation and comorbid conditions vary substantially among diagnosed individuals as do life outcomes, including response to intervention and the development of secondary or associated clinical features (e.g., Jeste & Geshwinde, 2014). Implementing interventions during early childhood may promote favourable outcomes (e.g., Kasari et al., 2015; Oono et al., 2013; Smith & Iadarola, 2015) and there is a strong push, worldwide, to reduce the average age of diagnosis so children with autism may be more quickly streamed toward targeted support, to mitigate the potentially disabling aspects of this condition.

Diagnosis is currently made on the basis of behavioural presentation, with the local average age of diagnosis in young children estimated as shortly after the 4^th^ birthday (i.e., in Australia; Bent et al., 2015). Nevertheless, early behavioural signs of autism may manifest in the first year of life, and become increasingly evident across the second year, such that there is high diagnostic stability from a child’s second birthday (e.g., Barbaro & Dissanayake, 2016).

Studies tracking the development of infants known to be at increased risk for ASD – most often, the infant siblings of children with an autism diagnosis – have identified potential markers of very early atypical development through the use of specialist technologies; for example, electroencephalography (EEG; e.g., Elsabbagh et al., 2012) and eye tracking (e.g., Jones & Klin, 2013).

Gazefinder is a relatively newly-developed eye-tracker thus far used in just a small number of published studies, all conducted in Japan (i.e., Fujioka et al., 2016; Fujisawa et al., 2014; Nishizato et al., 2017). These studies have included samples of typically developing (TD) children, children with autism, and adolescents/adults with and without autism, and have found significantly reduced fixation time toward socially-salient regions of interest within video scenes by participants with autism compared to non-autistic controls (e.g., eyes versus mouth region of a human face; people versus geometric shapes; attention toward point-at vs. distractor objects); preliminary data from Gazefinder which replicate work from other international groups using other eye-tracking technologies (e.g., Bertenthal et al., 2014; Gillespie-Smith et al., 2016; Rohlfing et al., 2012; Tenenbaum et al., 2013; Wagner et al., 2016).

Fujioka et al. (2016) estimated the sensitivity and specificity for Gazefinder for differentiating adolescents/adults with and without autism to be good – at 81% and 80%, respectively – and comparable to the properties of other non-eye-tracking measures seeking to differentiate adults with and without autism. These preliminary findings support proof-of-concept and the feasible use of Gazefinder, and highlight the potential utility of this technology to support diagnostic decision-making by differentiating participants with and without autism on the basis of their gaze patterns when viewing a brief (2-2½ minute) stimulus sequence. While the feasible use of Gazefinder has also been shown with child participants (Fujisawa et al., 2014; Nishizato et al., 2017), the efficacy of this technology to differentiate children with and without autism has yet to be demonstrated during the early childhood years – the developmental period during which parental concerns typically prompt a clinical diagnostic evaluation.

*6a Choice of Comparators*

The comparator will be a group of TD children – of the same mean and range of ages as the group of children with autism – for whom we can be confident there are no developmental concerns or behavioural signs of autism (i.e., ascertained via exclusion criteria and information provided on parent-report questionnaires).

*6b Explanation for Choice of Comparators*

As the primary objective of this trial is to evaluate the sensitivity and specificity of Gazefinder to differentiate young children with autism from those who are typically developing (TD), the comparator group is an age-matched sample of children with: (i) no known developmental conditions, (ii) moderate sub-threshold behavioural signs of autism, (iii) history of autism among first degree relatives (i.e., parent/s and sibling/s).

1. Objectives

The primary objective of this trial is to evaluate the accuracy – including sensitivity and specificity – of Gazefinder to differentiate young children with and without autism to evaluate its potential utility as an objective tool to supplement more subjective clinical measures currently in use (i.e., Autism Diagnostic Observation Schedule [ADOS]; Lord et al., 2012).

We hypothesise that Gazefinder will have good accuracy – including indicators of sensitivity and specificity both ≥80% – for differentiating children with autism from TD children.

A secondary, exploratory objective is to determine whether there may be characteristics of individuals/subgroups among the sample of children with autism – on factors such as age, number/type of autism or co-occurring symptoms, or amount of intervention received at the time of testing – for whom the accuracy of Gazefinder to differentiate autism vs. TD may be better/poorer. This is an exploratory objective; no a-priori hypotheses are specified.

1. Study Design

This is an observational trial conducted at a single time-point with planned comparison of participants – children aged 2 years 0 months to 4 years 11 months – recruited into two pre-defined groups: (i) those with a community diagnosis of autism, and (ii) those who are typically developing (TD). All children will complete a face-to-face assessment session – including Gazefinder eye-tracking assessment – and parents will complete standardised questionnaire measures. Further, children with autism will complete standard clinical assessments to characterise their symptoms/skills, in order to confirm appropriateness of the community diagnosis and to appraise heterogeneity in the sample.

Methods: Participants, Interventions, Outcomes

1. Study Setting

The trial will be conducted at LTU (Melbourne, Victoria) and TKI (Perth, Western Australia).

1. Eligibility Criteria

- 1. Inclusion Criteria

Children will be eligible to participant in the trial if they:

1. Are aged between 2 years 0 months and 4 years 11 months at Gazefinder assessment, and
2. (Autism group only) have a diagnosis of Autism Spectrum Disorder with supporting documentation (e.g., letter or report) from a community professional (e.g., paediatrician).
   1. Exclusion Criteria

Children will be excluded from participation in the trial if:

1. They have significant, uncorrected visual/hearing impairment;

Further, children will be excluded from participation *in the TD group* if:

1. Parents report concerns regarding their child’s development, related to autism or another developmental or genetic/neurological condition,
2. The child has an immediate family member (i.e., parent or sibling) with autism, and/or
3. There is evidence of moderate-level autism behaviours (i.e., operationalised as a score >12 on the parent-report Social Communication Questionnaire [SCQ; Rutter et al., 2003]).

Ideally, prior to participating in this study, the young children with autism will not have had significant intervention, and will not have clear signs of co-occurring neuro-developmental difficulties (e.g., attention deficit hyperactivity disorder [ADHD]) or be taking regular prescribed medication. However, it will not be possible to ensure children are enrolled in the trial *before* having had access to any intervention, nor to confidently screen out co-occurring conditions that may yet to have been identified/diagnosed at this young age. Hence, no exclusion criteria are stipulated regarding co-occurring conditions, prescribed medication, or the receipt of intervention prior to enrolment in the trial for children in the Autism group. Information on each of these factors will be collected via parent-report and will be included in characterisation of the sample and potentially in analysis of secondary objectives of the trial.

**Primary outcome**

The primary outcome concerns preferential attention to more (vs. less) socially salient ‘regions of interest’ (ROIs) that are pre-determined (i.e., pre-programmed) for each scene within the short 2½ minute Gazefinder stimulus sequence (referred to by JKC as ‘Scene 10’). We will evaluate sensitivity and specificity of Gazefinder to differentiate the group of children with autism from the group of TD children on the basis of their gaze patterns signalling (reduced) preferential attention toward more vs. less socially-salient ROIs.

**Secondary outcomes**

The secondary, exploratory outcome also concerns preferential attention (as described above). However, here, the context is to evaluate whether there might be characteristics of individuals/subgroups of children with autism – concerning factors such as age, number/type of autism or co-occurring symptoms, or amount of intervention received at the time of testing – for whom the accuracy of Gazefinder to differentiate autism vs. TD may be better/poorer. This is an exploratory objective; no a-priori hypotheses are specified.

1. Participant Timeline

Figure 1 shows the schedule of enrolment and assessments for participants in the trial and Figure 2 represents each participants’ journey through the trial. Parents/guardians who express interest in having their child participate in the study will be provided with a Participant Information and Consent Form (PICF), outlining study objectives, eligibility and participation requirements. A member of the research team will be available to answer any questions prior to parents providing signed informed consent for their child’s participation.

A face-to-face assessment will be scheduled for children with autism and their parents/guardians, at which children will complete standard play-based developmental assessments (i.e., Autism Diagnostic Observation Schedule [ADOS; Lord et al., 2012]; Mullen Scales of Early Learning [MSEL; Mullen, 1995]) and Gazefinder eye-tracking assessment. Parents will complete questionnaires (SCQ [Rutter et al., 2003]; Vineland Adaptive Behaviour Scales [VABS; Sparrow et al., 2005]; Child Behaviour Checklist [CBCL; Achenbach & Edelbrock, 1983]; MacArthur Bates Communicative Development Inventories [MCDI; Fenson et al., 2007]; and form concerning family background information). The assessments may be completed over multiple sessions if needed, and parents may elect to complete questionnaires at home and return these at the assessment session.

Parents of TD children will be provided with questionnaires (as above with exception of the MCDI) and asked to return these by post (along with the signed consent form). These will be checked to ensure the TD child has not met an exclusion criterion (e.g., SCQ score >12 suggesting moderate, sub-threshold autism-like behaviours). For those who remain eligible to participate, a brief face-to-face assessment will be scheduled, during which the Gazefinder eye-tracking assessment will be completed. Where a child is determined to be ineligible, this will be explained to parents who will be thanked for their time, with no face-to-face assessment will be scheduled.

1. Sample Size

A total of 200 participants will be enrolled in this trial: 100 children with autism and 100 TD children.

‘Accuracy’ is operationalised as the ‘Area Under the Curve’ (AUC) in the analysis of Receiver Operating Characteristics (ROC), which also provides sensitivity and specificity thresholds for correct classification. An AUC result ≥.80 may be considered to represent ‘good’ accuracy, and ≥.90, ‘excellent’ accuracy.

A sample of 40 children gives 95% power for a test of AUC ≥.80, assuming the adoption of *α* = .05, equal number of participants in each group, and a null hypothesis that accuracy is at chance (i.e., AUC = .50). Hence, with a target sample of 200 children – 100 per group – this trial is well-powered to meet its primary objective. Moreover, a sample of 200 gives >95% power for a test that accuracy is ‘excellent’ (i.e., AUC = .90) – again assuming the adoption of *α* = .05 and equal number of participants in each group – against the null hypothesis that accuracy is ‘good’ (i.e., AUC = .80).

Furthermore, enrolling a large sample of 100 children with autism will ensure variability in key characteristics (e.g., range of ages, symptom severity and cognitive/developmental ability, etc.) is likely to be captured in the sample, to permit secondary exploratory evaluation of whether there may be individuals/subgroups of participants for whom the accuracy of Gazefinder to differentiate Autism vs. TD may be better/poorer than the observe average estimate of accuracy.

| **Figure 1. Schedule of enrolment and assessments**   \|  \| **Participant Group** \| \| \| --- \| --- \| --- \| \|  \| **Autism** \| **TD** \| \| ENROLMENT: \|  \|  \| \| Eligibility check \| X \| X \| \| Informed consent \| X \| X \| \| QUESTIONNAIRES: \|  \|  \| \| Family information \| X \| X \| \| SCQ \| X \| X \| \| Exclusion check \|  \| X \| \| *VABS* \| X \| X \| \| *CBCL* \| X \| X \| \| *MCDI* \| X \|  \| \| CHILD ASSESSMENTS: \|  \|  \| \| *ADOS* \| X \|  \| \| *MSEL* \| X \|  \| \| *Gazefinder* \| X \| X \| | **Figure 2. Participant flow through trial**  Parent of potential participant receives information about trial  Parent contacts research team to have questions answered and for check on group membership and trial eligibility  Autism Group  Face-to-face session: signed consent, child assessments and parent-report questionnaires  Excluded: Child does not meet eligibility criteria  Excluded: Evidence of parent concerns about child and/or SCQ score >12. Parent contacted; thanked for their time and provided explanation of non-eligibility  Face-to-face session with child for Gazefinder  TD Group  Questionnaires and consent form sent to parent to complete and return |
| --- | --- | --- | --- | --- | --- | --- | --- | --- | --- | --- | --- | --- | --- | --- | --- | --- | --- | --- | --- | --- | --- | --- | --- | --- | --- | --- | --- | --- | --- | --- | --- | --- | --- | --- | --- | --- | --- | --- | --- | --- | --- | --- | --- | --- | --- | --- | --- | --- | --- |

1. Recruitment

At each site (LTU, TKI), parents of potentially eligible children with autism and typically developing children will be recruited via established channels, including participant registries, community engagement events, broad and targeted advertising through social media. Direct approach with be made to families with children who are likely to be eligible, such as those who are participants in other studies being conducted at the two sites, and those accessing on-site services (e.g., TKI CliniKids service; the LTU Community Children’s Centre including Autism Intervention Centre).

Methods: Data Collection, Management, Analysis

1. Data Collection Methods

Table 1 summarises the assessment battery for this trial, including parent-report questionnaires and direct behavioural assessments. Participants will typically complete these at one visit. However, where children with autism have recently completed a standardised cognitive/developmental assessment (i.e., through participation in another research study; e.g., within one month), we will seek parent permission to access these prior data rather than unnecessarily repeating this assessment with the child. Similarly, where children with autism have completed a standardised autism assessment (e.g., within four months), this will not be repeated if video footage exists to permit verification of research reliable coding. Further, if necessary it will be acceptable for a child with autism to complete the assessments across more than one session, with the aim that all assessments be completed within one month.

Gazefinder is an easy to use, portable eye-tracking apparatus around the size of a standard desktop computer and screen. The assessment protocol comprises presentation of a very brief (2½ minute) video sequence that is engaging and of high production quality. Like other commercially available eye-trackers, Gazefinder uses infrared light to determine corneal reflection and identify fixation and eye movement (tracking) patterns of the participant when observing the video sequence. No specific commands/instructions are issued, making this ideal for use with young children (including those with autism) who often enjoy watching on-screen animations and may have limited capacity and/or motivation to follow verbal instructions. Essentially, the participant experience of Gazefinder assessment is equivalent to watching a short animation on a computer/television screen. Children can be seated comfortably; independently at an appropriately-sized table, or on a carer’s lap.

The assessments with children will be undertaken by and under the supervision of researchers who have experience working with children with autism and TD children, and are trained in the reliable administration and scoring of these assessments. Cross-site checks will be conducted at least quarterly to ensure consistent administration and scoring (e.g., of the ADOS).

1. Data Management

Upon recruitment, participants will be allocated a code and source data – including paper protocols, digital video files and records created within Gazefinder – as well as electronic copies of source data, will be labelled by code only. The sole electronic file linking personal details with these code numbers will be stored on a password-protected database hosted at LTU, and accessible only to the research team.

During the trial, source data will be stored at the respective data collection site; LTU or TKI. Hard copies of assessment protocols will be stored in locked filing cabinets. Electronic source data (i.e., video footage, data files downloaded from Gazefinder) will be stored on a secure network drive accessible only to members of the trial team. Electronic entry of de-identified data will be onto REDCap, a secure, password-protected cloud-based database which is an approved data management platform of the sponsor organisation, accessible also to the partner data collection site and only by individuals who are part of the trial team.

A data audit process will be conducted at least bi-annually – including remote desktop and in-person auditing – to verify the accuracy and completeness of all data, including a cross-check between electronic records and hard copy source data. Details of the auditing procedure will be determined by the Steering Committee in the early stages of the project.

**Table 1. Summary of assessments**

| **Parent-Report Questionnaires** | | | |  | |  | |
| --- | --- | --- | --- | --- | --- | --- | --- |
| **Measure** | **Description** | **Variable** | **Group** | | **Role** | |  |
| **Social Communication Questionnaire (SCQ; Rutter et al., 2003)** | Parent-report measure of autism behaviours | Total score | Autism TD | | Eligibility (TD);  Sample characterisation;  Potential subgrouping (Autism) | |  |
| **Vineland Adaptive Behaviour Scales (VABS; Sparrow et al., 2005)** | Parent-report measure of child adaptive behaviour | Domain Age-Equivalence and Standard Scores (SS); Total SS | Autism TD | | Sample characterisation;  Potential subgrouping (Autism) | |  |
| **Child Behaviour Checklist (CBCL; Achenbach & Edelbrock, 1983)** | Parent-report measure of child challenging behaviours | Total Problems and Domain Standard Scores (SS) | Autism TD | | Sample characterisation;  Potential subgrouping (Autism) | |  |
| **MacArthur-Bates Communicative Development Inventories (MCDI; Fenson et al., 1993)** | Parent-report measure of child communication and language skills | Receptive and Expressive vocabulary counts; Total Gestures score | Autism | | Sample characterisation;  Potential subgrouping (Autism) | |  |
| **Family Background Questionnaire** | Parent-report on child and family background information | Various demographic characteristics for child and family | Autism TD | | Sample characterisation;  Potential subgrouping (Autism) | |  |
| **Child Assessment Measures** | | | |  | |  | |
| **Assessment Tool** | **Description** |  |  | |  | |  |
| **Mullen Scales of Early Learning (MSEL; Mullen, 1995)** | Standardised direct assessment of child developmental level | Domain Age-Equivalence Standard Scores | Autism | | Sample characterisation;  Potential subgrouping | |  |
| **Autism Diagnostic Observation Schedule – 2nd Edition (ADOS-2; Lord et al., 2012)** | Semi-structured direct assessment of child manifestation of autism symptoms | Domain and Total Algorithm scores and Calibrated Severity Score | Autism | | Corroboration of community diagnosis;  Sample characterisation;  Potential subgrouping | |  |
| **Gazefinder Eye Tracking Assessment: “Scene 10” 2½ minute sequence (JKC)** | Stimulus sequence presented via eye-tracker with gaze to pre-specified ROIs recorded automatically | Proportionate attention to less (vs. more) socially salient ROIs for all of ‘Scene 10’, and individual trials | Autism TD | | Outcome | |  |

1. Statistical Methods

*16a Outcome*

A preliminary analysis plan has been prepared prior to the commencement of participant recruitment/data collection, with plans to prepare a final, more detailed analysis plan – for approval by the Steering Committee/Data Safety and Management Committee – prior to the completion of participant recruitment and the commencement of data analysis. This final plan will be submitted to LTU HEC and included as an update to ANZCTR trial registration

Eye-tracking data to various pre-specified 'Regions of Interest' (ROIs; e.g., eyes vs. mouth of a human face) within trials of the stimulus sequence are automatically computed by Gazefinder software. Independent-samples t-tests will be used to compare average fixation times to key ROIs for participants in the Autism and TD groups. Receiver operating characteristic (ROC) area under the curve (AUC) analyses will be used to establish thresholds that maximise sensitivity and specificity for differentiating the Autism and TD groups across: (i) the overall 2.5-minute animation and (ii) the various trials/stimulus types (e.g., human face, social vs. non-social scenes). Interpretation of AUC will be as follows: Failed discrimination accuracy = .5-.6; Poor accuracy = .6-.7; Fair accuracy = .7-.8; Good accuracy = .8-.9; Excellent accuracy = .9-1.0. Sensitivity and specificity parameters above .8 will be considered good.

*16b Additional Analyses*

Descriptive statistics will be computed on demographic characteristics, key questionnaire data, and behavioural assessment scores, to characterise the samples of children with autism and TD children. Independent-samples t-tests (or non-parametric equivalent, if the data are skewed) will be computed to ascertain group matching/differentiation on key factors of interest (e.g., age, adaptive behaviour composite, etc.). Such characterisation of the children with Autism will inform the potential presence of meaningful subgroups (e.g., with greater vs. fewer core symptoms or co-occurring difficulties; older vs. younger children; those who have/have not yet received any autism-specific intervention, etc.) for whom Gazefinder may perform better/more poorly in differentiating Autism from TD. Again, eye-tracking data to the ROIs will be compared across subgroups via independent-samples t-test/ANOVA and ROC AUC analyses will be used to establish thresholds that maximise sensitivity and specificity for differentiating Autism subgroups from one another and the TD groups. Interpretation of AUC and sensitivity/specificity parameters will be as outlined above.

*16c Missing Data*

Missing data will be minimised by having parents complete questionnaire measures concurrently whilst researchers are completing direct assessments with the child, or by offering parents the opportunity to receive these in advance for completion at home. At the assessment, before the parent and child leave the centre, researchers will scan questionnaires for missing items and draw parents’ attention to these in the event that items were inadvertently skipped.

Where there is likelihood of missing data on standardised measures of child skill (e.g., due to behavioural signs of fatigue), researchers will offer breaks/multiple assessments in attempt to obtain complete and valid data on all scales of all measures.

Gazefinder assessment will typically occur as the *final* assessment of the session, after all other data have been collected. Children can view this passively and are likely to find this enjoyable/relaxing, and this will minimise the chances of missing data on other more demanding tasks which can be completed earlier in the session while children are likely to be most attentive. Further, this will ensure Gazefinder data are more likely to be captured for children who have *already completed* all other tasks, thereby minimising systematic reasons for missing other data that will be important for rigorously characterising the sample and may be pertinent to secondary ‘subgroup’ analysis.

Nevertheless, we will not exclude participants from the final trial sample and analysis if they have completed Gazefinder but have missing data on other scales/measures. At the point of analysis, we will conduct missing values analysis to determine if data are missing at random and, if appropriate, use Full Information Maximum Likelihood (FIML) or Multiple Imputation (MI) methods to impute values so as to maintain maximum sample size for analysis.

Methods: Monitoring

1. Data Monitoring

*17a Data Monitoring Committee*

A Steering /Data and Safety Management Committee will be convened to review the progress and conduct of the study. The composition of this committee will be determined in the early stages of the project. The role of this committee will be to approve the trial protocol, and members will meet twice per year to review the progress and conduct of the study, and agree on any necessary protocol changes. The steering committee will also be responsible for oversight of data monitoring and management, and coordinating data audits at each site.

*17b Interim Analysis*

Interim analysis will be conducted at least quarterly during the active data collection phase of the trial. The purpose of interim analysis will be to monitor characteristics of participants enrolled in the trial to (i) ensure basic inclusion/exclusion criteria are being met and (ii) appraise the extent to which participants in the Autism and TD groups are representative of the target populations and similar on key demographic characteristics (e.g., age, sex ratio, family socio-economic status, etc.). This will also allow appraisal of whether there may be subgroups within the Autism group, which might indicate a need to adjust our planned analysis of primary trial outcome, or further specify our planned analysis of secondary outcomes.

1. Harms

*18a Risk of Psychological, Emotional or Social Harms*

Children with autism may find some of the behavioural assessments challenging due to the nature of their core social-communication difficulties (i.e., difficulty communicating, difficulties with emotion regulation). The risk and potential stress is no greater than would arise in regular clinical practice or day-to-day home-based activities for these children. Members of the research team conducting the assessments, and supervisors, have substantial experience working with young children with autism, to support their participation in play-based activities, including by engaging parents expertise in how best to support and manage their young children.

Some parents of children with autism may find it challenging if their children are experiencing difficulties during the assessment (e.g., agitation at sitting to watch an animation via Gazefinder, or during parts of the standard clinical assessment). Members of the research team have substantial experience supporting parents during research assessments with their young children with autism, and again, the risk and potential stress to parents is no greater than would arise in regular clinical practice with families or in day-to-day home-based activities. Further, most parents will be accustomed to their child's behavioural difficulties, and in our experience, many parents find they are well supported by, and gain new insights from, observing skilled researchers engage their young children with autism in play-based assessment activities such as those in the current study.

We do not anticipate any risk of psychological, emotional or social harm to participants from the novel aspect of this trial: the Gazefinder eye-tracking protocol, which is completely non-invasive, and includes a very brief (~2.5 minutes) video sequence that is engaging and of high production quality.

It is also possible that parents of TD children may reflect on their child’s development while completing the questionnaire measures and subsequently report concerns they had not yet raised at eligibility screen prior to enrolment in the trial and/or discussed with a community health professional. It is also possible that some TD children will exceed the threshold of 12 on the parent-report SCQ, due to showing some (sub-threshold level of) behaviours also commonly displayed by children with Autism. Researchers will review the questionnaire responses of parents with enrolled TD children and discuss any issues noted and/or the elevated SCQ score to determine whether the parent does indeed have concerns about their child's development, and provide information regarding how to seek support in the local community if this is the case (e.g., speaking to the child's GP or Maternal and Child Health Nurse in the first instance).

We do not anticipate any risk of psychological, emotional or social harm to TD children or the research team. Nevertheless, any adverse event occurring during a child's participation in this trial will be reported to LTU HEC in a detailed written report, identifing the participant by assigned code. A report would include an assessment of the severity and causality of the adverse event in relation to the treatment protocol, as well as detailed information regarding steps taken by the Research Team and Steering Committee with regard to both the trial protocol and the participant involved.

*18b Physical Risks and Safety*

The novel aspect of this trial is the use of a bespoke eye-tracker - Gazefinder - manufactured by JVCKENWOOD Corporation (JKC), Japan, and provided to each of LTU and TKI sites for use in this research. A product user manual is provided (attached to this application) and outlines safety specifications regarding positioning and handling of the product by research staff, etc. The single Gazefinder that is already on site at LTU (OTARC labs) was tagged and tested on 19th July 2018 (Barcode ref: LT30-00020) and will be tested annually, following standard LTU practice for testing electrical equipment.

While Gazefinder is portable, JKC will provide 2 Gazefinder machines to each of the LTU and TKI sites, so that these can be situated in different locations associated with each centre (e.g., different clinic rooms/spaces on each campus) to minimise the need for researchers transport these for data collection whilst allowing flexibility for children/families to participate in the assessment at the most convenient on-site locations.

1. Auditing

A Steering/Data and Safety Management Committee will be convened to review the progress and conduct of the study. The composition of this committee will be determined in the early stages of the project and members will meet twice per year to approve the trial protocol, review the progress and conduct of the study, and agree on any necessary protocol changes. This committee will also be responsible for oversight of data monitoring and management, and coordinating data audits at each site. Data audit will be conducted bi-annually, by persons independent of the research team, and will include remote desktop auditing and in-person auditing to verify the accuracy and completeness of all data, including cross-check between electronic records and hard copy source data. Further details of this procedure will be determined by the Steering Committee in the early stages of the project.

ethics and Dissemination

1. Research Ethics Approval

This protocol (in Pre-registered Draft form) has been submitted for review by the sponsor to the institutional human ethics committee (LTU HEC). A final, registered version of the protocol and HEC submission will be forwarded for reciprocal approval by the HEC overseeing research conducted at the partner site (i.e., the Child and Adolescent Health Service Human Research Ethics Committee [CAHS HREC] for TKI). Any future amendments to the protocol will be reviewed by these ethics committees, and annual and adverse incident reports will be submitted as necessary.

1. Protocol Amendments

Any potential amendments to the protocol will be presented to the Steering Committee for approval. The Principal investigator will be responsible for submitting modification requests to LTU HREC and ANZCTR and providing evidence of approval for submission for reciprocal approval to CAHS HREC by the local TKI site lead. The Principal investigator will also be responsible keeping other stakeholders (e.g., JKC as the trial funder) informed of changes, as relevant.

1. Consent

Researchers will take signed informed consent from a parent/guardian on behalf of their child. Once a parent has expressed interest in having their child with autism participate in the study, an assessment session will be scheduled. At the start of this session (and throughout), parents will have the opportunity to ask any questions of the researchers. Parents will provide signed informed consent on behalf of their child before the assessment activities commence.

Once a parent with a TD child has expressed interest in having their child participate in the study, and have had the opportunity to ask questions of a researcher, questionnaires will be made available (e.g., by post; or given to the parent in person) along with a consent form to be signed and returned. Upon receipt of questionnaires and a second check that the TD child has not met an exclusion criterion for the study (e.g., SCQ score >12), a brief face-to-face session will be scheduled.

1. Confidentiality

Children will be assigned a unique identification code on enrolment in the trial with all information collected labelled with this code to ensure privacy. Completed assessment protocols/questionnaires will be kept in a locked filing cabinet, electronic copies of protocols/questionnaires and video footage of children completing assessments will be stored on a secure University network drive (accessible only to members of the project team), and summary data from assessments/questionnaires will be entered onto a secure online database accessible only by the research team. No forms/files will include children’s names, nor will the online database.

Child gaze data is automatically captured by Gazefinder, and subsequently .csv files with gaze to pre-specified ROIs will be downloaded by researchers onto a University computer and uploaded to the online database. Again, children’s names will not be entered into Gazefinder.

Personal data – names and contact details for children and parents/families – will only be recorded on consent forms, and stored separately from the de-identified research data; in a locked filing cabinets, and on a secure database at each site.

After the study is finished, a fully de-identified dataset will be shared with the funder (JKC) and a third party organisation, Pricewaterhouse Coopers (PwC), who will work together to conduct independent analyses of the data and prepare a report for submission to the Therapeutic Goods Administration (TGA). JKC wish to seek TGA approval for Gazefinder in order to be able to market this as a therapeutic device in Australia in the future. No identifying information will be shared with JKC/PwC.

The sponsor will keep all data for at least 15 years after completion of the study, and archive the anonymous data file with a University Research Repository. Members of the research team may seek to use this data again in the future related projects, after seeking permission from the LTU HREC. The research team plans to present results of this trial widely – including in peer-reviewed scientific journals, at local and international conferences, and shared with the parents/families of participating children and the general community (e.g., via traditional and social media). Again, no participant child’s name would be shared in such dissemination.

1. Declaration of Interests

The Sponsor, LTU, has received an industry research contract and funding from JKC, the manufacturer of Gazefinder, to independently conduct this clinical trial and will engage TKI via a Clinical Research Agreement to operate as a partner site for the conduct of this project. TKI will receive a portion of the funding provided from JKC to LTU for the conduct of the trial. JKC intends to use the results of this clinical trial to register Gazefinder with the Therapeutic Goods Administration (TGA) as a therapeutic device to aid the diagnosis of young children with autism.

The research contract and funding from JKC is not contingent on the outcome of the trial.

Funding will be provided in three instalments:

- Initial payment in January 2019 to support the first year of data collection
- Second payment in January 2020 to support the second (final) year of data collection
- Third (final) payment in April 2021 upon completion of analysis and provision of the final report by researchers to JKC.

A Steering/Data and Safety Management Committee will be convened – independent of the funding body – to review the progress and conduct of the study.

1. Access to Data

The sponsor, LTU, will retain ownership of the data collected. No limits will be imposed on the researchers’ access to and use of the data, subject to HREC approval. Source and electronic data will be made available by the researchers at each site for audit following procedures to be established by the Steering/Data and Safety Management Committee.

A fully de-identified dataset will be shared by the research team with JKC and PwC. This will allow JKC to conduct further, independent analysis of the dataset and preparation a Dossier for submission to the TGA to seek approval for promotion of Gazefinder as medical device.

1. Dissemination Policy

*26a Trial Results*

The research team will seek to disseminate the results of this study widely, in scientific peer-reviewed publications, but also to other stakeholders (e.g., the public, the autistic and autism communities, government bodies, etc.) through social and traditional media outlets.

By contractual agreement, both the research teams (at LTU and TKI) and the funder (JKC) may publish and/or present the results of the trial, providing that the publication does not disclose confidential or commercially sensitive information and is not misleading to the public.

The funder, JKC, with third-party organisation PwC will prepare the results of this study in a Dossier for submission to the Therapeutic Goods Administration (TGA).

*26b Authorship*

Authorship on publications and outputs arising from this trial will be determined based on significant contributions to the design, conduct, and interpretation of the trial. All authors will be acknowledged as appropriate.

There are three main categories of publication that may result from the study, and the authorship team will vary accordingly:

- Reports of the main outcomes of the study – publications that report on the sensitivity/specificity of Gazefinder to different children with autism from TD children. Members of the research teams at LTU and TKI and developers of the Gazefinder technology will be acknowledged as appropriate.
- Reports addressing one aspect of the study in detail, using data from the entire study;
- Reports of data derived from a single site or independent of the trial, but using trial data.

Any authorship disputes will be addressed by the Steering/Data and Safety Management Committee. The research team has no intention to employ professional medical writers.

*26c Reproducible Research*

The trial protocol will be made publicly available via registration with the ANZCTR. Following completion, a de-identified copy of the data set will be provided with a written report on the trial to JKC and PwC who will conduct independent analyses to confirm the trial results in preparing a Dossier for submission to the TGA. Appendices

1. Informed consent Materials

Participant Information and Consent Form attached.

1. References

Baio, J., Wiggins, L., Christensen, D.L., et al. (2018). Prevalence of Autism Spectrum Disorder among children aged 8 years – Autism and Developmental Disabilities Monitoring Network, 11 Sites, United States, 2014. *MMWR Surveillance Summaries, 67,* 1-23. DOI: <http://dx.doi.org/10.15585/mmwr.ss6706a1>.

Barbaro, J., & Dissanayake, C. (2016). Diagnostic stability of Autism Spectrum Disorder in toddlers prospectively identified in a community-based setting: Behavioural characteristics and predictors of change over time. *Autism, 21,*830-840*.*

Bertenthal, B.I., Boyer, T.W. & Harding, S. (2014). When do infants begin to follow a point? *Developmental Psychology, 50,* 2036-2048.

Elsabbagh, M., Mercure, E., Hudry, K., et al. (2012). Infant neural sensitivity to dynamic eye gaze is associated with later emerging autism. *Current Biology, 22,* 338-342.

Fenson, L., Marchman, V.A., Thal, D.J., et al. (2007). MacArthur-Bates Communicative Development Iventories: User’s guide and technical manual (2nd ed.). Baltimore: Paul H. Brookes.

Fujioka, T., Inohara, K., Okamoto, Y., et al. (2016). Gazefinder as a clinical supplementary tool for discriminating between autism spectrum disorder and typical development in male adolescents and adults. *Molecular Autism, 7*. DOI: 10.1186/s13229-016-0083-y

Fujisawa, T.X., Tanaka, S., Saito, D.N., et al. (2014). Visual attention for social information and salivary oxytocin levels in preschool children with autism spectrum disorders: An eye-tracking study. *Frontiers in Neuroscience.* DOI: [10.3389/fnins.2014.00295](https://doi.org/10.3389/fnins.2014.00295).

Gillespie-Smith, K., Boardman, J.P., Murray, I.C., et al. (2016). Multiple measures of fixation on social content in infancy: Evidence for a single social cognitive construct. *Infancy, 21,* 241-257.

Jeste, S., & Geschwind, D. (2014). Disentangling the heterogeneity of autism spectrum disorder through genetic findings. *Nature Review of Neurology, 10,* 74–81. doi:10.1038/nrneurol.2013.278

Jones, W. & Klin, A. (2013). Attention to eyes is present but in decline in 2-6 month-olds later diagnosed with autism. *Nature, 504,* 427-431.

Kasari, C., Gulsrud, A., Paparella, T., et al. (2015). Randomized comparative efficacy study of parent-mediated interventions for toddlers with autism. *Journal of Consulting and Clinical Psychology, 83,* 554–563. doi:10.1037/a0039080

Lord, C., Risi, S., Lambrecht, L., et al. (2000). The Autism Diagnostic Observation Schedule-generic: A standard measure of social and communication deficits associated with the spectrum of autism. Journal of Autism and Developmental Disorders, 30, 205–223. doi:10.1023/ A:1005592401947

Nishizato, M., Fujisawa, T.X., Kosaka, H. & Tomoda, A. (2017). Developmental changes in social attention and oxytocin levels in infants and children. *Scientific Reports, 7*, 2540.

Oono, I., Honey, E., & McConachie, H. (2013). Parentmediated early intervention for young children with autism spectrum disorders (ASD). *Cochrane Database of Systematic Reviews.* doi:10.1002/14651858.CD009774. pub2

Rohlfing, K.J., Longo, M.R., & Bertenthal, B.I. (2012). Dynamic pointing triggers shifts of visual attention in young infants. *Developmental Science, 15*, 426-435.

Rutter, M., Bailey, A., & Lord, C. (2003). *Social Communication Questionnaire*. Los Angeles: Western Psychological Services.

Smith, T., & Iadarola, S. (2015). Evidence base update for Autism Spectrum Disorder. *Journal of Clinical Child and Adolescent Psychology, 44,* 897-922. doi:10.1080/15374416.2015.1077448

Sparrow, S.S., Cicchetti, D.V., & Balla, D.A. (2005). *Vineland Adaptive Behavior Scales (2nd ed.).* Circle Pines, MN: American Guidance Service.

Tenenbaum E.J., Shah, R.J., Sobel, D.M., et al., (2013). Increased focus on the mouth among infants in the first year of life: A longitudinal eye-tracking study. *Infancy, 18,* 534-553.

Wagner, J., Luyster, R.J., Moustapha, H., et al. (2016). Differential attention to faces in infant siblings of children with Autism Spectrum Disorder and associations with later social and language ability. *International Journal of Behavioral Development, 42,* 83-92.

# **FINAL TRIAL PROTOCOL**


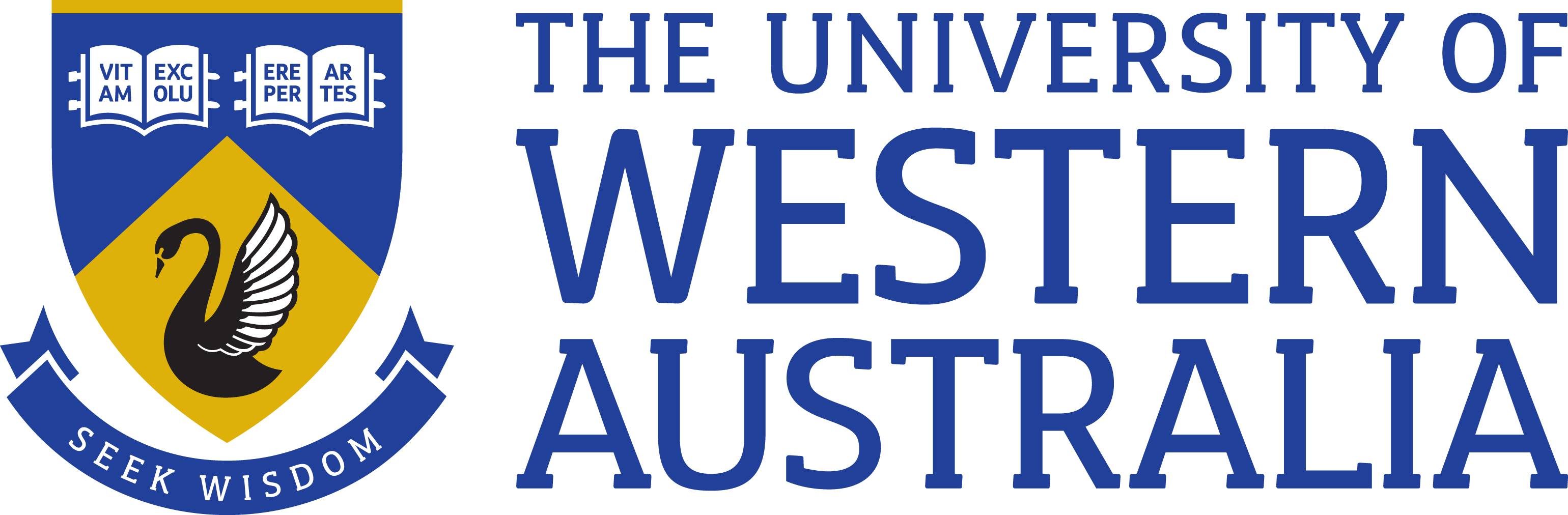

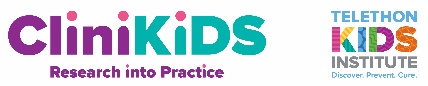


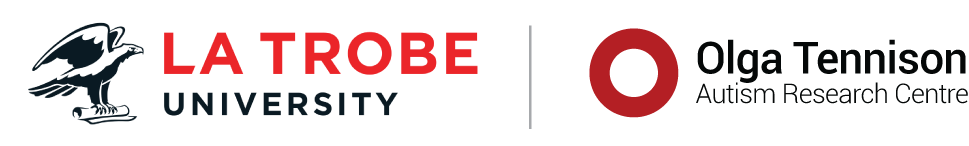


| protocol |
| --- |
| Clinical Trial of Gazefinder with Young Children with Autism and Typical Development |
| Protocol Number: U1111-1228-1050  Version 4  Date: 09/03/2021 |
|  |
|  |
| **CONFIDENTIAL**  This document is confidential and the property of La Trobe University. No part of it may be transmitted, reproduced, published, or used without prior written authorisation from the institution.  **Statement of Compliance**  This document is a protocol for a research project. This study will be conducted in compliance with all stipulation of this protocol, the conditions of the ethics committee approval, the NHMRC National Statement on ethical Conduct in Human Research (2007) and the Note for Guidance on Good Clinical Practice (CPMP/ICH-135/95). |

# Table of Contents

[Table of Contents 2](#_Toc39496973)

[Administrative Information 4](#_Toc39496974)

[1. Title 4](#_Toc39496975)

[2. Trial Registration 4](#_Toc39496976)

[2a Registry 4](#_Toc39496977)

[2b data set 4](#_Toc39496978)

[3. Protocol Version 6](#_Toc39496979)

[4. Funding 6](#_Toc39496980)

[5. Roles and Responsibilities 6](#_Toc39496981)

[5a Contributorship 6](#_Toc39496982)

[5b Sponsor Contact Information 7](#_Toc39496983)

[5c Sponsor and Funder 7](#_Toc39496984)

[5d Committees 7](#_Toc39496985)

[Introduction 8](#_Toc39496986)

[6. Background and Rationale 8](#_Toc39496987)

[6a Choice of Comparators 9](#_Toc39496988)

[6b Explanation for Choice of Comparators 9](#_Toc39496989)

[7. Objectives 9](#_Toc39496990)

[8. Study Design 9](#_Toc39496991)

[Methods: Participants, Interventions, Outcomes 9](#_Toc39496992)

[9. Study Setting 9](#_Toc39496993)

[10. Eligibility Criteria 9](#_Toc39496994)

[11. Participant Timeline 11](#_Toc39496995)

[12. Sample Size 12](#_Toc39496996)

[13. Recruitment 12](#_Toc39496997)

[Methods: Data Collection, Management, Analysis 12](#_Toc39496998)

[14. Data Collection Methods 12](#_Toc39496999)

[15. Data Management 13](#_Toc39497000)

[16. Statistical Methods 14](#_Toc39497001)

[16a Outcome 14](#_Toc39497002)

[16b Additional Analyses 15](#_Toc39497003)

[16c Missing Data 15](#_Toc39497004)

[Methods: Monitoring 16](#_Toc39497005)

[17. Data Monitoring 16](#_Toc39497006)

[17a Steering Committee and Data Monitoring and Safety Committee 16](#_Toc39497007)

[17b Interim Analysis 16](#_Toc39497008)

[18. Harms 17](#_Toc39497009)

[18a Risk of Psychological, Emotional or Social Harms 17](#_Toc39497010)

[18b Physical Risks and Safety 17](#_Toc39497011)

[19. Auditing 18](#_Toc39497012)

[ethics and Dissemination 18](#_Toc39497013)

[20. Research Ethics Approval 18](#_Toc39497014)

[21. Protocol Amendments 18](#_Toc39497015)

[22. Consent 19](#_Toc39497016)

[23. Confidentiality 19](#_Toc39497017)

[24. Declaration of Interests 20](#_Toc39497018)

[25. Access to Data 20](#_Toc39497019)

[26. Dissemination Policy 20](#_Toc39497020)

[26a Trial Results 20](#_Toc39497021)

[26b Authorship 21](#_Toc39497022)

[26c Reproducible Research 21](#_Toc39497023)

[Appendices 22](#_Toc39497024)

[27. Informed consent Materials 22](#_Toc39497025)

[28. References 22](#_Toc39497026)

# Administrative Information

## Title

Clinical Trial of Gazefinder with Young Children with Autism and Typical Development

## Trial Registration

### 2a Registry

This trial is registered with the Australian and New Zealand Clinical Trials Registry (ANZCTR) with approval from the La Trobe University Human Research Ethics Committee (LTU HREC).

Trial Identifier: ACTRN12619000317190

LTU HREC Approval: 19-027

### 2b data set

| **Data Category** | **Information** |
| --- | --- |
| Primary registry and study identifying number | Australian and New Zealand Clinical Trials Registry Identifier: ACTRN12619000317190 |
| Date of registration in primary registry | 01/03/2019 |
| Secondary identifying numbers | Unique Trial Identifier: U111-1228-1050 |
| Source(s) of monetary/material support | JVCKENWOOD Corporation (JKC, Japan) |
| Primary sponsor | La Trobe University |
| Secondary sponsor(s) | N/A |
| Contact for public queries | Dr Kristelle Hudry |
| Contact for scientific queries | Dr Kristelle Hudry |
| Public title | Could "Gazefinder" Eye-Tracking Technology Support the Earlier Identification and Diagnosis of Autism in Young Children? |
| Scientific title | Clinical Trial examining the sensitivity and specificity of Gazefinder for identifying young children with autism |
| Countries of recruitment | Australia |
| Health condition(s) or problem(s) studied | The accurate identification of autism in young children |
| Intervention(s) | N/A |
| Key inclusion and exclusion criteria | **Children with autism:**  Inclusion: Child age between 2 years 0 months and 4 years 11 months; diagnosis of autism from a community professional.  Exclusion: Uncorrected visual/hearing impairment  **Typically developing (TD) children:**  Inclusion: Child age between 2 years 0 months and 4 years 11 months  Exclusion: Uncorrected visual/hearing; parent-reported family history of autism and/or presence of another developmental condition and/or moderate-level autism behaviours. |
| Study type | Observational  Primary purpose: Natural History / Screening Duration: Cross-sectional Selection: Defined Population Timing: Both prospective and retrospective Patient Registry: No |
| Recruitment Status | Recruiting |
| Date of first enrolment | 08/03/2019 |
| Date of last data collection | Anticipated March 2021 |
| Target sample size | 200 |
| Primary outcome(s) | Accuracy of Gazefinder to differentiate autism from TD |
| Exploratory secondary outcomes | Influence of characteristics of individual children with autism – such as age, core and associated symptom presentation, cognitive ability, etc. – on the accuracy of Gazefinder to differentiate autism from TD |

## Protocol Version

| **Issue Date:** | 09 03 2021 |
| --- | --- |
| **Protocol amendment number:** | Version 4 |
| **Author(s):** | Hudry, K., Bent, C., Chetcuti, L., Whitehouse, A. |

Revision Chronology:

| **Date of change** | **Summary of changes** |
| --- | --- |
| 22 Jan 2019 | Original (pre-registration draft) |
| 29 Mar 2019 | - Updated basic information (trial registration numbers, versions, reciprocal approval obtained for work at TKI site etc). - Further details provided on composition, role and meeting frequency of Steering Committee & Data Monitoring and Safety Committee - Adjustment of period within which standardised autism assessments will not be repeated (from four to six months) - Further details provided on proposed audit process - Clarification around interim data sharing with funder (vs. routine review of descriptive sample characterisation by research team) - Correction regarding number of Gazefinder units provided by funder to each site |
| 01 May 2020 | - Addition of a second, out-of-schedule, interim sharing of de-identified data to allow JKC to progress algorithm development work in light of COVID-19-related pause to trial recruitment/data collection (see Section 17b) |
| 11 Feb 2021 | - Updated basic information (HEC reference; anticipated data collection completion date, etc.) - Updated information reflecting COVID-19 related adjustments to data collection, including necessary shift to remote desktop audit by PWC, and recent HREC approval to engage a new data collection site (December 2020; with interstate travel to North-West Tasmania by LTU researchers in early 2021) |
| 09 Mar 2021 | - Specification of project work beyond data collection (e.g., completion of contract research report including sample characterisation analysis; development of best-fit diagnostic algorithm/s; interpretation of algorithm against sample characterisation data [with joint expertise]). Delineation of analysis plan and process. Key updates are within:   - Section 6 (Background and Rationale; recent publication by Tsuchiya et al., 2021);   - Section 7 (Objectives; clarification and further justification for aims, including drawing on recent published research);   - Section 10 (Primary and Secondary outcomes; clarification and further justification for analysis plan drawing on recent published research);   - Section 12 (Sample Size; elaborating on power calculation and rationale for recruitment of sample larger than required for adequate power to meet the primary trial objective, including to allow for potential post-hoc exclusion of cases prior to algorithm development work);   - Table 1 (Summary of Assessments; updates to correct/clarify measurement derived from selected tools);   - Section 16 (Statistical Methods; clarification of timeline and process);   - Section 24 (Declaration of Interests; clarification of planned collaborative work to complete the trial, beyond the delivery of data collection and the contract research report by the trial sites to the funder);   - Sections 25 & 26 (Access to Data, Dissemination Policy, & Reproducible Research; update to clarify ownership of and access to various aspects of the data generated within the trial, that dissemination may include joint publications as appropriate, and delineating responsibility for various aspects of the data between the funder and sponsor/researcher). |

## Funding

| JVCKENWOOD Corporation (JKC)  3-12 Moriya-cho,  Kanagawa-ku  Kokohama-shi  Kanagawa  221-0022 Japan | Gazefinder is manufactured by JKC, Japan, and supplied to two trial sites – La Trobe University (LTU) and Telethon Kids Institute (TKI) – for the purpose of conducting this trial. JKC is funding the running costs of the trial, including providing equipment and research staff salary costs. The management, analysis and reporting of the study will be conducted independently of JKC. |
| --- | --- |

##

## Roles and Responsibilities

### 5a Contributorship

| **Author Name** | **Summary of contribution** |
| --- | --- |
| **Dr Kristelle Hudry**  La Trobe University | KH conceived of the study and co-led a feasibility pilot with AW which has informed the current trial, and will coordinate the project |
| **Dr Catherine Bent**  La Trobe University | CB contributed to study design and will be involved in data collection and analysis |
| **Ms Lacey Chetcuti,** La Trobe University | LC contributed data collection as part of the feasibility pilot, contributed to study design and will be involved in data collection and analysis |
| **Prof. Andrew Whitehouse** Telethon Kids Institute | AW conceived of the study, co-led a feasibility pilot with KH which has informed the current trial and will lead activities at the partner site (Telethon Kids Institute, TKI) |

### 5b Sponsor Contact Information

| **Study Sponsor** | La Trobe University |
| --- | --- |
| **Sponsor’s Reference number (if applicable)** | N/A |
| **Contact name** | Kristelle Hudry |
| **Address** | C/O La Trobe University, School of Psychology and Public Health, Bundoora, VIC 3083 |
| **Telephone** | 9479 5649 |
| **Email** | **xxxxxxxxxxxxxxxxxxxx** |

###

### 5c Sponsor and Funder

LTU is the study sponsor and will oversee the conduct of the trial, including study design, data collection, management, analysis and interpretation, writing of the report and publication of results in peer-reviewed publications. Telethon Kids Institute (TKI) is a partner site for data collection under a clinical research agreement with LTU (executed 20.03.2019). JKC as the funder of the trial will receive a report and de-identified copy of the raw data following the completion of the trial data collection, at the conclusion of the contract research agreement period (01.04.2021), as well as interim reports on study progress, and an interim de-identified copy of raw data at approximate trial mid-point (with a second interim data share also conducted in mid 2020 when a necessary pause in data collection was encountered due to COVID-19). Both LTU/TKI and JKC may publish and/or present the results of the trial, providing that the publication does not disclose confidential or commercially sensitive information and is not misleading to the public. Joint LTU/TKI and JKC publication and/or presentation may also be made, as appropriate, where meaningful contributions to output reflect the work of personnel at each of these organisations (e.g., source data collection by LTU/TKI researchers and algorithm development work by JKC statisticians/software engineers).

### 5d Committees

**Principal Investigator and Research Staff**

The joint research team (LTU and TKI sites) meets at least quarterly to review project activities (e.g., recruitment and data collection), to ensure the conduct of the project is on track, protocols are consistent across sites, and that any arising issues are promptly addressed. The research teams are responsible for participant recruitment and data collection at each site, and site leads are jointly responsible for the preparation of study reports.

**Steering Committee**

A Steering Committee reviews the progress and conduct of the study. The composition of this committee includes site research leads (LTU and TKI), a representative of the Sponsor (LTU) and representatives from an external auditing body, Pricewaterhouse Coopers (PwC), with representatives of the funder (JKC) requesting the opportunity to attend and observe these meetings. Given this composition, Steering Committee meetings for the trial are subsumed within planned, monthly 4-party (LTU, TKI, PwC, JKC) project progress meetings, hosted by PwC, during which the progress and conduct of the trial is reviewed and any necessary protocol changes are agreed. This committee is also be responsible for coordinating data audits at each site, including regular a) within-trial, cross-site remote audits (LTU-TKI), and b) external in person audits (PwC visiting each of LTU and TKI; conducted remotely from 2020 given COVID-19 related travel restrictions) (see below).

**Data Monitoring and Safety Committee**

A Data Monitoring and Safety Committee (DMSC) is convened to conduct independent review the conduct of the study, with particular focus on data quality and safety/adverse event reporting. The composition of this committee was determined within the first 6 months of the trial. The committee includes individuals independent of the study research team with expertise in a) statistics, b) subject matter (i.e., childhood autism), and c) clinical trials. Members meet twice per year with LTU/TKI research site leads also in attendance.

# Introduction

## Background and Rationale

Autism Spectrum Disorder (ASD; hereafter, *autism*) is a lifelong developmental condition affecting at least 1% of people (e.g., Baio et al., 2018), characterized by impairments in social interaction, verbal and nonverbal communication skills, alongside the presence of restricted/repetitive patterns of behaviour (APA, 2013). Core symptom presentation and comorbid conditions vary substantially among diagnosed individuals as do life outcomes, including response to intervention and the development of secondary or associated clinical features (e.g., Jeste & Geshwinde, 2014). Implementing interventions during early childhood may promote favourable outcomes (e.g., Kasari et al., 2015; Oono et al., 2013; Smith & Iadarola, 2015) and there is a strong push, worldwide, to reduce the average age of diagnosis so children with autism may be more quickly streamed toward targeted support, to mitigate the potentially disabling aspects of this condition.

Diagnosis is currently made on the basis of behavioural presentation, with the local average age of diagnosis in young children estimated as shortly after the 4^th^ birthday (i.e., in Australia; Bent et al., 2015). Nevertheless, early behavioural signs of autism may manifest in the first year of life, and become increasingly evident across the second year, such that there is high diagnostic stability from a child’s second birthday (e.g., Barbaro & Dissanayake, 2016).

Studies tracking the development of infants known to be at increased risk for ASD – most often, the infant siblings of children with an autism diagnosis – have identified potential markers of very early atypical development through the use of specialist technologies; for example, electroencephalography (EEG; e.g., Elsabbagh et al., 2012) and eye tracking (e.g., Jones & Klin, 2013).

Gazefinder is a relatively newly-developed eye-tracker thus far used in just a small number of published studies, all conducted in Japan (i.e., Fujioka et al., 2016; Fujisawa et al., 2014; Nishizato et al., 2017). These studies have included samples of typically developing (TD) children, children with autism, and adolescents/adults with and without autism, and have found significantly reduced fixation time toward socially-salient regions of interest within video scenes by participants with autism compared to non-autistic controls (e.g., eyes versus mouth region of a human face; people versus geometric shapes; attention toward point-at vs. distractor objects); preliminary data from Gazefinder which replicate work from other international groups using other eye-tracking technologies (e.g., Bertenthal et al., 2014; Gillespie-Smith et al., 2016; Rohlfing et al., 2012; Tenenbaum et al., 2013; Wagner et al., 2016).

Fujioka et al. (2016) estimated the sensitivity and specificity for Gazefinder for differentiating adolescents/adults with and without autism to be good – at 81% and 80%, respectively – and comparable to the properties of other non-eye-tracking measures seeking to differentiate adults with and without autism. These preliminary findings support proof-of-concept and the feasible use of Gazefinder, and highlight the potential utility of this technology to support diagnostic decision-making by differentiating participants with and without autism on the basis of their gaze patterns when viewing a brief (2-2½ minute) stimulus sequence. While the feasible use of Gazefinder has also been shown with child participants (Fujisawa et al., 2014; Nishizato et al., 2017), the efficacy of this technology to differentiate children with and without autism has yet to be demonstrated during the early childhood years – the developmental period during which parental concerns typically prompt a clinical diagnostic evaluation.

**Update 09.03.2021:** Approaching the conclusion of data collection for this trial, a newly- published paper from Tsuchiya et al. (2021) provides performance estimates of 74% sensitivity, 80% specificity, and 78% accuracy for Gazefinder to differentiate children with and without autism (ages spanning 5- to 17-years; n = 39 and 102, respectively). This research was conducted in Japan, by researchers at seven collaborating Universities and affiliated Hospital/Clinical services. Recruitment and data collection occurred over a 6-month period during 2018, with submission of the manuscript to *Frontiers in Neurology* for peer review in September 2020, accepted for publication in December 2020, and subsequently published in January 2021. Two employees of JKC (SH and MN) are co-authors on the publication, noted as having contributed in particular to study concept and design, and data analysis and interpretation (i.e., through expertise in statistical analysis for software algorithm development). The conduct of other aspects of the research – including securing funding, undertaking clinical evaluation and measurement, and leading manuscript drafting – are noted as having been conducted exclusively by contributors other than the JKC employees. On the basis of the detailed statistical analysis plan undertaken in this similar published study (albeit with a sample of older children and smaller sized autism subgroup, and different stimulus sequence to that included in the current trial), we propose to a parallel plan for the conduct and reporting of analysis on data collected in the current trial (detailed below).

### 6a Choice of Comparators

The comparator will be a group of TD children – of the same mean and range of ages as the group of children with autism – for whom we can be confident there are no developmental concerns or behavioural signs of autism (i.e., ascertained via exclusion criteria and information provided on parent-report questionnaires).

### 6b Explanation for Choice of Comparators

As the primary objective of this trial is to evaluate the sensitivity and specificity of Gazefinder to differentiate young children with autism from those who are typically developing (TD), the comparator group is an age-matched sample of children with: (i) no known developmental conditions, (ii) moderate sub-threshold behavioural signs of autism, (iii) history of autism among first degree relatives (i.e., parent/s and sibling/s).

## Objectives

The primary objective of this trial is to evaluate the accuracy – including sensitivity and specificity – of Gazefinder to differentiate young children with and without autism to evaluate its potential utility as an objective tool to supplement more subjective clinical measures currently in use (i.e., Autism Diagnostic Observation Schedule [ADOS]; Lord et al., 2012). Fujioka et al. (2016) estimated 81% sensitivity and 80% specificity for gaze data captured by Gazefinder to differentiate adolescents/adults with and without autism. These data reflected participants’ attention to prespecified more (vs. less) socially salient ‘regions of interest’ (ROIs), based on a stimulus sequence comprising a combination of social attention, preferential [social vs. non-social] attention, referential/joint attention, and attention to biological motion point light displays. Very recently, Tsuchiya et al. (2021) reported performance parameters for differentiating *children* with and without autism (aged 5- to 17-years) of 74% sensitivity and 80% specificity (and 78% accuracy). These were based on a slightly different stimulus sequence – tapping social attention and preferential (social vs. non-social) attention only – and used a more complex statistical approach to derive a ‘best-fit’ diagnostic algorithm (also on the basis of attention to pre-specified more vs. less socially salient ROIs). This included several steps including extracting candidate attributes from among the various ROI data that differentiated children with and without autism, creating and iteratively testing the performance of a number of potential diagnostic ‘best-fit’ algorithms, computing performance parameters of the final algorithm and its performance under iterative ‘leave-one-out’ approach, and evaluating performance with a small independent participant sample.

Clinical assessments used to inform autism diagnosis, and commonly considered to represent the ‘gold-standard’ among currently available tools show good to excellent predictive validity against diagnostic clinical best estimate. For example, the ADOS-2 manual reports sensitivity metrics ranging between .77 and .97 and specificity metrics ranging between .77 and .94 for the differentiation of autism and ASD vs. non-spectrum outcomes, across four diagnostic algorithms developed for young children aged between 2 years 6 months and 4 years 11 months (total n=982). This was with the single exception of lower specificity metrics (.19 to .50) for the algorithm developed for children with few/no words and non-verbal developmental abilities ≤15-months’ equivalence.

This trial is designed to test the hypothesis that a Gazefinder best-fit algorithm developed on the basis of ‘Scene 10A’ stimulus presentation will have good accuracy (i.e., indicators of sensitivity and specificity both ≥80%) for differentiating 2- to 4-year-old children with autism from similarly-aged TD children. An AUC result ≥.80 may be considered to represent ‘good’ accuracy, and ≥.90, ‘excellent’ accuracy (Carter, Pan, Rai & Galandiuk, 2016).

A secondary, exploratory objective is to determine whether there may be characteristics of individuals/subgroups among the sample of children with autism – related to such factors as age, number/type of autism or co-occurring symptoms, amount of intervention received at the time of testing, capacity for sustained attention – for whom the accuracy of Gazefinder to differentiate autism vs. TD may be better/poorer. This is an exploratory objective; no a-priori hypotheses are specified. However, the target sample size for recruitment/data collection, and the few exclusion criteria have been set to support the engagement of large and varied sample of children with autism – such as many present to clinical services for diagnostic assessment/opinion at this developmental period – that will enable the examination of such a secondary objective.

## Study Setting

This is an observational trial conducted at a single time-point with planned comparison of participants – children aged 2 years 0 months to 4 years 11 months – recruited into two pre-defined groups: (i) those with a community diagnosis of autism, and (ii) those who are typically developing (TD). All children complete a face-to-face assessment session – including Gazefinder eye-tracking assessment – and parents complete standardised questionnaire measures. Further, children with autism complete standard clinical assessments to characterise their symptoms/skills, in order to confirm appropriateness of the community diagnosis and to appraise heterogeneity in the sample.

# Methods: Participants, Interventions, Outcomes

## Study Setting

Trial data collection is conducted at researchers based at LTU (Melbourne, Victoria) and TKI (Perth, Western Australia). While most data collection has been conducted at LTU and TKI sites, following COVID-19 related delays to study progress, researchers secured HREC and other approvals in late 2020 to conduct trial recruitment and data collection interstate in 2021 (NW Tasmania).

## Eligibility Criteria

- 1. Inclusion Criteria

Children are eligible to participant in the trial if they:

1. Are aged between 2 years 0 months and 4 years 11 months at Gazefinder assessment, and
2. (Autism group only) have a diagnosis of Autism Spectrum Disorder with supporting documentation (e.g., letter or report) from a community professional (e.g., paediatrician).
   1. Exclusion Criteria

Children will be excluded from participation in the trial if:

1. They have significant, uncorrected visual/hearing impairment;

Further, children will be excluded from participation *in the TD group* if:

1. Parents report concerns regarding their child’s development, related to autism or another developmental or genetic/neurological condition,
2. The child has an immediate family member (i.e., parent or sibling) with autism, and/or
3. There is evidence of moderate-level autism behaviours (i.e., operationalised as a score >12 on the parent-report Social Communication Questionnaire [SCQ; Rutter et al., 2003]).

Ideally, prior to participating in this study, the young children with autism will not have had significant intervention (such as would be the case for children presenting for a diagnostic assessment/opinion), and will not have clear signs of co-occurring neuro-developmental difficulties (e.g., attention deficit hyperactivity disorder [ADHD]) or be taking regular prescribed medication (such as might indicate more complex differential diagnosis or particular phenotypic difference plausibly altering gaze behaviour and attention control). However, it will not be possible to ensure children are enrolled in the trial *before* having had access to any intervention, nor to confidently screen out co-occurring conditions that may yet to have been identified/diagnosed at this young age. Hence, no exclusion criteria are stipulated regarding co-occurring conditions, prescribed medication, or the receipt of intervention prior to enrolment in the trial for children in the Autism group. Information on each of these factors will be collected via parent-report and will be included in characterisation of the sample and potentially in analysis of secondary objectives of the trial.

11.1 Primary outcome

The primary outcome concerns preferential attention to more (vs. less) socially salient ‘regions of interest’ (ROIs) that are pre-determined (i.e., pre-programmed) for each scene within the short 2½ minute Gazefinder stimulus sequence (referred to by JKC as ‘Scene 10’). We will evaluate sensitivity and specificity of Gazefinder to differentiate the group of children with autism from the group of TD children on the basis of their gaze patterns signalling (reduced) preferential attention toward more vs. less socially-salient ROIs.

**Update 09.03.2021:** Following publication of the report by Tsuchiya et al. (2021) who trialled Gazefinder to differentiate children aged 5-17 years, with and without autism, we have proposed to update our planned analysis for data collected in the current trial to parallel the clear and detailed procedure reported by this group, and drawing on machine learning statistical methods beyond the expertise of the primary data collection teams, but for which JKC employees have demonstrated expertise. Aspects of Tsuchiya et al.’s study mirror the current trial, including that: a) study design was undertaken jointly across researchers at University sites and affiliated clinical services; b) recruitment and data collection was conducted by researchers with clinical autism expertise (i.e., independently of JKC as product developer/manufacturer [and also funder of the current trial]), and c) with JKC employees’ contribution including to provide input at early project planning meetings and subsequent Steering Group meetings over the course of the research, and to provide technical assistance at trial start-up (including a Gazefinder apparatus for each of the current study sites). While our original plan was that the clinical research teams would conduct preliminary analysis of child gaze data, captured by Gazefinder, and provide this as part of the contract research report to JKC – with JKC thereafter conducting independent analysis for algorithm development – we propose that any analysis of gaze data by the clinical research team (beyond simple feasibility assessment; i.e., of overall tracking rates) would be redundant given subsequent sophisticated analysis work planned by JKC. That is, the development and testing of a single ‘best-fit diagnostic algorithm’, and potential alternative algorithms that perform more accurately for particular subgroups of children with autism – both achievable through machine learning statistical methods that are the expertise of JKC personnel (as evidenced by Tsuchiya et al., 2021) – would be more robust and informative than a set of stimulus/trial-dependent tests as feasible for our clinical research team. Hence, we rephrase and specify our statement of primary outcome as follows:

The primary outcome construct concerns child preferential attention to more (vs. less) socially salient ‘regions of interest’ (ROIs) that are pre-determined (i.e., pre-programmed) for each scene within the short 2½ minute Gazefinder stimulus sequence (referred to by JKC as ‘Scene 10A’). Toward the primary objective of this trial, a single ‘best-fit diagnostic algorithm’ will be derived on the basis of raw Gazefinder data collected in this trial (i.e., the .csv file exported directly from Gazefinder apparatus after each child’s assessment), with the aim of achieving robust differentiation of children with autism vs. TD children. The accuracy (incl. sensitivity and specificity parameters) of this best-fit algorithm will be evaluated as follows: no predictive value = .50; poor accuracy = .51-.69; fair accuracy = .70-.79; good accuracy = .80-.89; excellent accuracy = .90-.99; and perfect accuracy = 1.0 (Carter et al., 2016).

11.2 Secondary outcomes

The secondary, exploratory outcome also concerns preferential attention (as described above). However, here, the context is to evaluate whether there might be characteristics of individuals/subgroups of children with autism – concerning factors such as age, number/type of autism or co-occurring symptoms, or amount of intervention received at the time of testing – for whom the accuracy of Gazefinder to differentiate autism vs. TD may be better/poorer. This is an exploratory objective; no a-priori hypotheses are specified.

**Update 09.03.2021:** While the primary objective is to derive a single ‘best-fit diagnostic algorithm’ that differentiates children with autism from TD children with good accuracy, the heterogeneity evident in autism is such that a single algorithm may have different performance properties for differentiating from individuals/subgroups of children with autism who share particular characteristics vs. others from TD children. That is, a single ‘best-fit’ algorithm may perform better under certain conditions than others. We will explore the characteristics of children/subgroups for whom the single ‘best-fit diagnostic algorithm’ has returned inaccurate classification. A series of algorithms each applied to children with different clinical/behavioural algorithms may together perform more accurately than one single algorithm (i.e., as is the case for the existing behavioural assessment representing among the ‘gold-standard’ autism diagnostic assessments; the ADOS-2, which includes algorithms selected on the basis of child age and/or language ability; Lord et al., 2012). The secondary outcome construct remains unchanged, but will again be evaluated through the joint efforts of JKC personnel who conduct algorithm development analytic work and members of the clinical research teams who will undertake initial sample characterisation analytic work and subsequently support the interpretation of algorithm performance against participant/sample clinical/behavioural characteristics.

## Participant Timeline

Figure 1 shows the schedule of enrolment and assessments for participants in the trial and Figure 2 represents each participants’ journey through the trial. Parents/guardians who express interest in having their child participate in the study are provided with a Participant Information and Consent Form (PICF), outlining study objectives, eligibility and participation requirements. A member of the research team is available to answer any questions prior to parents providing signed informed consent for their child’s participation.

A face-to-face assessment is scheduled for children with autism and their parents/guardians, at which children will complete standard play-based developmental assessments (i.e., Autism Diagnostic Observation Schedule [ADOS; Lord et al., 2012]; Mullen Scales of Early Learning [MSEL; Mullen, 1995]) and Gazefinder eye-tracking assessment. Parents complete questionnaires (SCQ [Rutter et al., 2003]; Vineland Adaptive Behaviour Scales [VABS; Sparrow et al., 2005]; Child Behaviour Checklist [CBCL; Achenbach & Edelbrock, 1983]; MacArthur Bates Communicative Development Inventories [MCDI; Fenson et al., 2007]; and form concerning family background information). The assessments may be completed over multiple sessions if needed, and parents may elect to complete questionnaires at home and return these at or after the assessment session.

Parents of TD children are provided with questionnaires (as above with exception of the MCDI) and asked to return these by post (along with the signed consent form) or at a scheduled brief assessment session. These are checked to ensure the TD child has not met an exclusion criterion (e.g., SCQ score >12 suggesting moderate, sub-threshold autism-like behaviours). For those who remain eligible to participate, a brief face-to-face assessment occurs, during which the Gazefinder eye-tracking assessment is completed. Where a child is determined to be ineligible, this is explained to parents who are thanked for their time, with no face-to-face assessment necessary.

| Figure 1. Schedule of enrolment and assessments   \|  \| **Participant Group** \| \| \| --- \| --- \| --- \| \|  \| **Autism** \| **TD** \| \| ENROLMENT: \|  \|  \| \| Eligibility check \| X \| X \| \| Informed consent \| X \| X \| \| QUESTIONNAIRES: \|  \|  \| \| Family information \| X \| X \| \| SCQ \| X \| X \| \| Exclusion check \|  \| X \| \| *VABS* \| X \| X \| \| *CBCL* \| X \| X \| \| *MCDI* \| X \|  \| \| CHILD ASSESSMENTS: \|  \|  \| \| *ADOS* \| X \|  \| \| *MSEL* \| X \|  \| \| *Gazefinder* \| X \| X \| | Figure 2. Participant flow through trial  Parent of potential participant receives information about trial  Parent contacts research team to have questions answered and for check on group membership and trial eligibility  Autism Group  Face-to-face session: signed consent, child assessments and parent-report questionnaires  Excluded: Child does not meet eligibility criteria  Excluded: Evidence of parent concerns about child and/or SCQ score >12. Parent contacted; thanked for their time and provided explanation of non-eligibility  Face-to-face session with child for Gazefinder  TD Group  Questionnaires and consent form sent to parent to complete and return |
| --- | --- | --- | --- | --- | --- | --- | --- | --- | --- | --- | --- | --- | --- | --- | --- | --- | --- | --- | --- | --- | --- | --- | --- | --- | --- | --- | --- | --- | --- | --- | --- | --- | --- | --- | --- | --- | --- | --- | --- | --- | --- | --- | --- | --- | --- | --- | --- | --- | --- |

## Sample Size

**The target sample for this trial is 200 participants: 100 children with autism and 100 TD children.**

**‘Accuracy’ is operationalised as the ‘Area Under the Curve’ (AUC) in the analysis of Receiver Operating Characteristics (ROC), which also provides sensitivity and specificity thresholds for correct classification. An AUC result ≥.80 may be considered to represent ‘good’ accuracy, and ≥.90, ‘excellent’ accuracy (Carter et al., 2016).**

**A sample of 40 children gives 95% power for a test of AUC ≥.80, assuming the adoption of *α* = .05, equal number of participants in each group, and a null hypothesis that accuracy is at chance (i.e., AUC = .50). Hence, with a target sample of 200 children – 100 per group – this trial is well-powered to meet its primary objective.**

We propose a target sample of 200 children – 100 each with autism and TD. A sample of 200 participants gives >95% power for a test that accuracy is ‘excellent’ (i.e., AUC = .90) – again assuming the adoption of *α* = .05 and equal number of participants in each group – against the null hypothesis that accuracy is ‘good’ (i.e., AUC = .80). Enrolling a large sample of 200 children (100 in each group) will ensure variability in key characteristics (particularly for children with autism; e.g., range of ages, symptom severity and cognitive/developmental ability, etc.) is captured in the sample, and will allow for the possible need to exclude data collected for some children prior to analysis for a ‘best-fit diagnostic algorithm’. Examples of circumstances that might indicate data exclusion include (but are not limited to):

- 1. Individual children returning low rates of overall tracking data (due to poor general attention, overactivity, non-compliance, and other behavioural difficulties, etc.) that preclude the inclusion of their gaze data to the pre-specified ROIs in statistical analysis toward algorithm development) whilst retaining adequate sample size for the primary hypothesis test;
  2. The determination that there are particular individual children who are outliers, or subgroups of children whose presence otherwise skews the distribution/s of the participant sample/s, and where this is determined to be better managed by omitting participants prior to analysis rather than by retaining their data and controlling for the effects of the given characteristic in other ways (e.g., statistically controlling for effect; testing the performance of the derived algorithm to accurately classify child/ren with a particular characteristic or profile, etc). That is, the large sample size will particularly support the secondary exploratory evaluation of whether there may be individuals/subgroups of participants for whom the accuracy of Gazefinder to differentiate Autism vs. TD may be better/poorer than the observe average estimate of accuracy.

## Recruitment

**At each site (LTU, TKI, and with extension of trial data collection in 2021 to NW Tasmania), parents of potentially eligible children with autism and typically developing children will be recruited via established channels, including participant registries, community engagement events, broad and targeted advertising through social media. Direct approach with be made to families with children who are likely to be eligible, such as those who are participants in other studies being conducted at the two sites, and those accessing on-site services (e.g., TKI CliniKids service; the LTU Community Children’s Centre including Autism Intervention Centre; autism intervention services supported through St Giles in NW Tasmania).**

# Methods: Data Collection, Management, Analysis

## Data Collection Methods

Table 1 summarises the assessment battery for this trial, including parent-report questionnaires and direct behavioural assessments. Participants will typically complete these at one visit. However, where children with autism have recently completed a standardised cognitive/developmental assessment (i.e., through participation in another research study; e.g., within one month), we will seek parent permission to access these prior data rather than unnecessarily repeating this assessment with the child. Similarly, where children with autism have completed a standardised autism assessment (e.g., within six months), this will not be repeated if video footage exists to permit verification of research reliable coding. Further, if necessary it will be acceptable for a child with autism to complete the assessments across more than one session, with the aim that all assessments be completed within one month.

**Table 1. Summary of assessments**

| **Parent-Report Questionnaires** | | |  |  |
| --- | --- | --- | --- | --- |
| **Measure** | **Description** | **Variable/s** | **Group** | **Role** |
| **Social Communication Questionnaire (SCQ; Rutter et al., 2003)** | Parent-report measure of autism behaviours | Total score | Autism TD | Eligibility (TD);  Sample characterisation;  Potential subgrouping (Autism) |
| **Vineland Adaptive Behaviour Scales (VABS; Sparrow et al., 2005)** | Parent-report measure of child adaptive behaviour | Domain Age-Equivalence and Standard Scores (SS); Total SS | Autism TD | Sample characterisation;  Potential subgrouping (Autism) |
| **Child Behaviour Checklist (CBCL; Achenbach & Edelbrock, 1983)** | Parent-report measure of child challenging behaviours | Total Problems and Domain Standard Scores (SS) | Autism TD | Sample characterisation;  Potential subgrouping (Autism) |
| **MacArthur-Bates Communicative Development Inventories (MCDI; Fenson et al., 1993)** | Parent-report measure of child communication and language skills | Receptive and Expressive vocabulary counts; Total Gestures score | Autism | Sample characterisation;  Potential subgrouping (Autism) |
| **Family Background Questionnaire** | Parent-report on child and family background information | Various demographic characteristics for child and family | Autism TD | Sample characterisation;  Potential subgrouping (Autism) |
| **Child Assessment Measures** | | |  |  |
| **Assessment Tool** | **Description** | **Variable/s** | **Group** | **Role** |
| **Mullen Scales of Early Learning (MSEL; Mullen, 1995)** | Standardised direct assessment of child developmental level | Early Learning Composite Standard Score; domain Age-Equivalence (AE) scores; Non-Verbal and Verbal AEs; Overall, Non-Verbal and Verbal Developmental Quotientts (DQ: AE / chron. Age * 100) | Autism | Sample characterisation;  Potential subgrouping |
| **Autism Diagnostic Observation Schedule – 2nd Edition (ADOS-2; Lord et al., 2012)** | Semi-structured direct assessment of child manifestation of autism symptoms | Domain and Total Algorithm scores and Calibrated Severity Score | Autism | Corroboration of community diagnosis;  Sample characterisation;  Potential subgrouping |
| **Gazefinder Eye Tracking Assessment: “Scene 10” 2½ minute sequence (JKC)** | Stimulus sequence presented via eye-tracker with gaze to pre-specified ROIs recorded automatically | Calibration; number of attempts; tracking rates; observation notes (including related to adverse events)  ‘Best-fit’ diagnostic algorithm/s derived from Gazefinder output .csv file (per participant) returning child gaze toward pre-specified ‘Scene 10A’ ROIs | Autism TD | Feasibility  Adverse events  Outcome/s |

Gazefinder is an easy to use, portable eye-tracking apparatus around the size of a standard desktop computer and screen. The assessment protocol comprises presentation of a very brief (2½ minute) video sequence that is engaging and of high production quality. Like other commercially available eye-trackers, Gazefinder uses infrared light to determine corneal reflection and identify fixation and eye movement (tracking) patterns of the participant when observing the video sequence. No specific commands/instructions are issued, making this ideal for use with young children (including those with autism) who often enjoy watching on-screen animations and may have limited capacity and/or motivation to follow verbal instructions. Essentially, the participant experience of Gazefinder assessment is equivalent to watching a short animation on a computer/television screen. Children can be seated comfortably; independently at an appropriately-sized table, or on a carer’s lap.

The assessments with children will be undertaken by and under the supervision of researchers who have experience working with children with autism and TD children, and are trained in the reliable administration and scoring of these assessments. Cross-site checks will be conducted to ensure consistent application of data collection protocols (e.g., administration and scoring of the ADOS).

## Data Management

Upon recruitment, participants will be allocated a code and source data – including paper protocols, digital video files and records created within Gazefinder – as well as electronic copies of source data, will be labelled by code only. The sole electronic file linking personal details with these code numbers will be stored on a password-protected database hosted at LTU, and accessible only to the research team.

During the trial, source data will be stored at the respective data collection site; LTU or TKI. Hard copies of assessment protocols will be stored in locked filing cabinets. Electronic source data (i.e., video footage, data files downloaded from Gazefinder) will be stored on a secure network drive accessible only to members of the trial team. Electronic entry of de-identified data will be onto REDCap, a secure, password-protected cloud-based database which is an approved data management platform of the sponsor organisation, accessible also to the partner data collection site and only by individuals who are part of the trial team.

A data audit process will be conducted regularly (approximately monthly; but variable depending on rate of data collection across the trial period) to verify the accuracy and completeness of all data. This will include:

- Remote desktop audit by members of the alternate-site research team (i.e., LTU for TKI site data; TKI for LTU site data), for both identifying information (i.e., consent forms, ASD diagnostic reports, assessment video footage) and de-identified data (i.e., scanned copies of source paperwork and electronic data entry into REDCap).
- In-person audit by independent external party (PwC) for each of LTU and TKI sites, for de-identified data only (i.e., source paperwork including confirmation of participant eligibility, scanned copies of source paperwork, and electronic data entry into REDCap).

## Statistical Methods

A preliminary analysis plan has been prepared prior to the commencement of participant recruitment/data collection. The more detailed analysis plan outlined here has been developed prior to the completion of participant recruitment and data collection, and the sharing of data with JKC for analysis. This updated, more detailed plan, has been approved by the Steering Committee and Data Safety and Management Committee, before submission of this modified/updated Clinical Trial Protocol (version 4) for approval by LTU HREC and updated of ANZCTR trial registration (or Open Science Framework in the event of potential delays to timely ANZCTR registration approval associated with workflow impacts of COVID-19).

### 17a Sample characterisation

Preliminary analysis will include the calculation of descriptive statistics on demographic characteristics, key questionnaire data, and behavioural assessment scores, to characterise the samples of children with autism and TD children. Independent-samples t-tests (or non-parametric equivalent, if the data are skewed) will be computed to ascertain group matching/differentiation on key factors of interest (e.g., age, adaptive behaviour composite, etc.). Such characterisation of the children with Autism will inform the potential presence of meaningful subgroups (e.g., with greater vs. fewer core symptoms or co-occurring difficulties; older vs. younger children; those who have/have not yet received any autism-specific intervention, etc.) for whom Gazefinder may perform better/more poorly in differentiating Autism from TD.

**Update 09.03.2020:** Additional preliminary analysis will involve the examination of data informing feasibility of child assessment with Gazefinder; principally the overall Tracking Rate score (i.e., proportion of stimulus sequence time for each child, during which gaze data were captured) but also other available parameters such as calibration rate, number of attempts to calibrate, record of adverse events, observation notes of child behaviour during Gazefinder assessment etc. Where possible, descriptive statistics for these factors will be computed, and independent-samples t-tests (or non-parametric equivalent) will be computed to ascertain between-group similarity/difference. For each group, correlations will be computed (or cross-tabulation/contingency analysis conducted, for categorical data), to evaluate child characteristics associated with Gazefinder assessment feasibility.

These data and analysis will be conducted by the clinical research team, independently of JKC as product developer/manufacturer and funder of this trial. These sample characterisation results will be presented within the Contract Research Report (due for delivery on 1st April, 2021, along with raw data exported from Gazefinder following each child’s assessment).

### 17b Primary outcome

**Update 09.03.2020:** The key analysis for these trial data will follow the process outlined by Tsuchiya et al. (2021) who conducted a similar recent study of the potential for Gazefinder to differentiate children with and without autism aged 5 years and older. Independent of the clinical research team, JKC personnel with statistical expertise will analyse raw data from each child’s Gazefinder assessment, and the categorical grouping variable to develop a ‘best-fit’ diagnostic algorithm to differentiate children in the ASD group from those in the TD group. The algorithm will be based on gaze data recorded to the various pre-specified 'Regions of Interest' (ROIs; e.g., eyes vs. mouth of a human face) automatically computed by Gazefinder software within trials of the Scene 10A stimulus sequence. Receiver operating characteristic (ROC) area under the curve (AUC) analyses will be used to establish thresholds that maximise sensitivity and specificity for differentiating the groups . Interpretation of AUC will follow Carter et al. (2016): no predictive value = .50; poor accuracy = .51-.69; fair accuracy = .70-.79; good accuracy = .80-.89; excellent accuracy = .90-.99; and perfect accuracy = 1.0.

These data and analysis will be conducted by JKC as developer/manufacturer of Gazefinder, independent of the clinical research team. Algorithm development work will be undertaken following delivery of the Contract Research Report and raw Gazefinder data (i.e., April 2021 and beyond) and performance results will be shared by JKC at ongoing Steering Committee meetings.

### 17b Secondary analyses

Again, given the heterogeneity of behavioural/clinical presentation of autism even in early childhood, it is possible that a single ‘best-fit’ diagnostic algorithm will perform better/more poorly at differentiating certain individuals/subgroups of children with autism from TD same-age peers.

**Update 09.03.2020:** Using the raw data from each child’s Gazefinder assessment, and the detailed sample characterisation data provided by the clinical research team (in the Contract Research Report; anticipated 1st April 2021), JKC personnel and the clinical research teams will work together: (a) to interpret the performance of the single ‘best-fit’ diagnostic algorithm, including to understand the reasons for which individual/subgroups of children may have been mis-classified, and (b) to determine whether the overall performance of this technology to accurately differentiate children with and without autism might be improved through the delineation of one or more additional algorithms.

It seems plausible that different algorithms might perform better for accurately classifying children with autism as such, where clinical presentation suggests a particular cognitive profile (e.g., in the context of intellectual disability, or above-average intellectual ability), for children with different socio-demographic characteristics (e.g., girls vs. boys; younger vs. older children), or for those with attention profiles resulting in different engagement with Gazefinder assessment (e.g., children attending to >70% or <70% of stimulus presentation), or on the basis of attention to different parts of the stimulus sequence (i.e., first minute of presentation; or trials of a particular type). Again, ROC AUC analyses will be used and interpreted as outlined above.

This secondary, exploratory analysis work will be conducted jointly by JKC personnel and the clinical research team. As with analysis toward the primary outcome, JKC will take primary responsibility for any further algorithm development work, and the clinical research team will support interpretation of the performance of the primary, single ‘best-fit’ diagnostic algorithm and of any additional diagnostic algorithms developed to improve accuracy of the overall Gazefinder system. Report of the diagnostic algorithm/s and interpretation of the associated performance data will be presented in a jointly-authored report (anticipated completion July 2021).

### 17d Missing Data

Missing data will be minimised by having parents complete questionnaire measures concurrently whilst researchers are completing direct assessments with the child, or by offering parents the opportunity to receive these in advance for completion at home. At the assessment, before the parent and child leave the centre, researchers will scan questionnaires for missing items and draw parents’ attention to these in the event that items were inadvertently skipped.

Where there is likelihood of missing data on standardised measures of child skill (e.g., due to behavioural signs of fatigue), researchers will offer breaks/multiple assessments in attempt to obtain complete and valid data on all scales of all measures.

Gazefinder assessment will typically occur as the *final* assessment of the session, after all other data have been collected. Children can view this passively and are likely to find this enjoyable/relaxing, and this will minimise the chances of missing data on other more demanding tasks which can be completed earlier in the session while children are likely to be most attentive. Further, this will ensure Gazefinder data are more likely to be captured for children who have *already completed* all other tasks, thereby minimising systematic reasons for missing other data that will be important for rigorously characterising the sample and may be pertinent to secondary ‘subgroup’ analysis.

Nevertheless, we will not exclude participants from the final trial sample and analysis if they have completed Gazefinder but have missing data on other scales/measures. At the point of analysis, we will conduct missing values analysis to determine if data are missing at random and, if appropriate, use Full Information Maximum Likelihood (FIML) or Multiple Imputation (MI) methods to impute values so as to maintain maximum sample size for analysis.

# Methods: Monitoring

## Data Monitoring

### 18a Steering Committee and Data Monitoring and Safety Committee

The Steering Committee and DMSC will review the progress and conduct of the study. Steering Committee meetings will be subsumed within planned, monthly 4-party (LTU, TKI, PwC, JKC) project progress meetings, hosted by PwC, during which the progress and conduct of the trial will be reviewed and any necessary protocol changes will be agreed. This committee will also be responsible for coordinating data audits each site. The DMSC will be independent of the study research team. Members will meet twice per year to monitor the progress of the trial, with particular focus on data quality and safety/adverse event reporting.

### 18b Interim Analysis

Interim sharing of de-identified raw data with the funder, JKC, will occur at approximate trial mid-point. This will permit manufacturers of Gazefinder to begin work on algorithm development while the trial is still underway. Interim data sharing will be on the understanding that results arising from the manufacturer’s analysis (JKC) are not disclosed to the Sponsor (LTU) or to the researchers conducting the trial (LTU, TKI sites). The precise time at which interim data are shared will be agreed by the Steering Committee, and will depend on the rate of progress with recruitment into each of the Autism and TD groups (e.g., end of 1^st^ year of data collection, or upon reaching n=100 participants [min = 50 per group]).

**Update 01.05.2020:** In the event of extraordinary circumstances brought by COVID-19, delaying progress to new data collection for the Clinical Trial, an additional out-of schedule interim data share was requested by JKC to permit continued work on algorithm development with the larger participant sample (including more children with Autism tested on Gazefinder) available in early 2020. This was proposed on 27^th^ March, 2020 at a meeting of the Steering Committee who felt the request was justified, and was subsequently also endorsed by the DMSC at a meeting on 29^th^ April 2020. Both the Steering Committee and DMSC agreed this second, out-of-schedule interim data share, should only occur following PWC having carried out an audit as usual on *all* new data and following protocol modification and received acknowledgement of such from LTU HEC.

No interim analysis will be conducted by the research team. Descriptive characteristics of participants will be examined regularly, and reported at Steering Committee and DMSC meetings, to monitor characteristics of participants enrolled in the trial. This will permit (i) confirmation that basic inclusion/exclusion criteria are being met and (ii) appraisal of the extent to which participants in the Autism and TD groups are representative of the target populations and similar on key demographic characteristics (e.g., age, sex ratio, family socio-economic status, etc.) and (iii) appraisal of whether there may be subgroups within the Autism group, which might indicate a need to adjust planned analysis of the primary trial outcome, or further specify our planned analysis of secondary outcomes.

## Harms

### 19a Risk of Psychological, Emotional or Social Harms

Children with autism may find some of the behavioural assessments challenging due to the nature of their core social-communication difficulties (i.e., difficulty communicating, difficulties with emotion regulation). The risk and potential stress is no greater than would arise in regular clinical practice or day-to-day home-based activities for these children. Members of the research team conducting the assessments, and supervisors, have substantial experience working with young children with autism, to support their participation in play-based activities, including by engaging parents expertise in how best to support and manage their young children.

Some parents of children with autism may find it challenging if their children are experiencing difficulties during the assessment (e.g., agitation at sitting to watch an animation via Gazefinder, or during parts of the standard clinical assessment). Members of the research team have substantial experience supporting parents during research assessments with their young children with autism, and again, the risk and potential stress to parents is no greater than would arise in regular clinical practice with families or in day-to-day home-based activities. Further, most parents will be accustomed to their child's behavioural difficulties, and in our experience, many parents find they are well supported by, and gain new insights from, observing skilled researchers engage their young children with autism in play-based assessment activities such as those in the current study.

We do not anticipate any risk of psychological, emotional or social harm to participants from the novel aspect of this trial: the Gazefinder eye-tracking protocol, which is completely non-invasive, and includes a very brief (~2.5 minutes) video sequence that is engaging and of high production quality.

It is also possible that parents of TD children may reflect on their child’s development while completing the questionnaire measures and subsequently report concerns they had not yet raised at eligibility screen prior to enrolment in the trial and/or discussed with a community health professional. It is also possible that some TD children will exceed the threshold of 12 on the parent-report SCQ, due to showing some (sub-threshold level of) behaviours also commonly displayed by children with Autism. Researchers will review the questionnaire responses of parents with enrolled TD children and discuss any issues noted and/or the elevated SCQ score to determine whether the parent does indeed have concerns about their child's development, and provide information regarding how to seek support in the local community if this is the case (e.g., speaking to the child's GP or Maternal and Child Health Nurse in the first instance).

We do not anticipate any risk of psychological, emotional or social harm to TD children or the research team. Nevertheless, any adverse event occurring during a child's participation in this trial will be reported to LTU HEC in a detailed written report, identifying the participant by assigned code. A report would include an assessment of the severity and causality of the adverse event in relation to the treatment protocol, as well as detailed information regarding steps taken by the Research Team and Steering Committee with regard to both the trial protocol and the participant involved.

### 19b Physical Risks and Safety

The novel aspect of this trial is the use of a bespoke eye-tracker - Gazefinder - manufactured by JVCKENWOOD Corporation (JKC), Japan, and provided to each of LTU and TKI sites for use in this research. A product user manual is provided (attached to HREC application) and outlines safety specifications regarding positioning and handling of the product by research staff, etc. The Gazefinder already on site at LTU (OTARC labs) prior to the start of this trial was tagged and tested on 19th July 2018 (Barcode ref: LT30-00020) and will be tested annually, following standard LTU practice for testing electrical equipment.

JKC provided an additional Gazefinder machine to each of the LTU and TKI sites, for dedicated use for this trial.

## Auditing

The Steering Committee and DMSC will review the progress and conduct of the study. The Steering Committee – comprising representatives from the trial research teams (LTU, TKI), sponsor (LTU), and external auditor (PwC), with representatives from the funder (JKC) also observing – will be responsible for coordinating data audits each site, while the DMSC will comprise individuals independent of the study research team and will also oversee trial progress with a particular focus on data quality and safety/adverse event reporting.

Data auditing will occur approximately monthly, but is likely to vary over the course of the trial depending on the rate of data collection at each site. The purpose will be to verify the accuracy and completeness of all data.

- Remote desktop audit – conducted by members of the alternate-site research team – will concern both identifying records (i.e., consent forms, ASD diagnostic reports, assessment video footage) and de-identified data (i.e., scanned copies of source paperwork and electronic data entry into REDCap).
- In-person audit – conducted by the independent external party (PwC) for each of LTU and TKI sites – will concern de-identified data only (i.e., source paperwork including confirmation of participant eligibility, scanned copies of source paperwork, and REDCap electronic data entry). From 2020, independent external party audit will also necessarily be via remote desktop access, in light of COVID-19 related travel restrictions.

# Ethics and Dissemination

## Research Ethics Approval

A pre-registration draft of this protocol was submitted for review by the sponsor to the institutional human ethics committee (LTU HREC) and in seeking reciprocal approval for conduct of the research at the partner site (University of Western Australia for TKI site). The current revised version and any future amendments to the protocol will be reviewed by these ethics committees, and annual and adverse incident reports will be submitted as necessary.

## Protocol Amendments

Any potential amendments to the protocol will be presented to the Steering Committee for approval. The Principal investigator will be responsible for submitting modification requests to LTU HREC and ANZCTR and providing evidence of approval for submission for reciprocal approval to the University of Western Australia by the local TKI site lead. The Principal investigator will also be responsible keeping other stakeholders (e.g., JKC as the trial funder) informed of changes, as relevant.

## Consent

Researchers will take signed informed consent from a parent/guardian on behalf of their child. Once a parent has expressed interest in having their child with autism participate in the study, an assessment session will be scheduled. At the start of this session (and throughout), parents will have the opportunity to ask any questions of the researchers. Parents will provide signed informed consent on behalf of their child before the assessment activities commence.

Once a parent with a TD child has expressed interest in having their child participate in the study, and have had the opportunity to ask questions of a researcher, questionnaires will be made available (e.g., by post; or given to the parent in person) along with a consent form to be signed and returned. Upon receipt of questionnaires and a second check that the TD child has not met an exclusion criterion for the study (e.g., SCQ score >12), the brief face-to-face session will be conducted.

## Confidentiality

Children will be assigned a unique identification code on enrolment in the trial with all information collected labelled with this code to ensure privacy. Completed assessment protocols/questionnaires will be kept in a locked filing cabinet, electronic copies of protocols/questionnaires and video footage of children completing assessments will be stored on a secure University network drive (accessible only to members of the project team), and summary data from assessments/questionnaires will be entered onto a secure online database accessible only by the research team. No forms/files will include children’s names, nor will the online database.

Child gaze data is automatically captured by Gazefinder, and subsequently .csv files with gaze to pre-specified ROIs will be downloaded by researchers onto a University computer and uploaded to the online database. Again, children’s names will not be entered into Gazefinder.

Personal data – names and contact details for children and parents/families – will only be recorded on consent forms, and stored separately from the de-identified research data; in a locked filing cabinets, and on a secure database at each site.

During the course of data collection for the trial, de-identified raw data (source and electronic data entry) will be made available to Pricewaterhouse Coopers (PwC), at in-person visits to each of the LTU and TKI sites, for the purposes of enabling independent audit (or via remote desktop audit, from 2020, in the context of COVID-19 related travel restrictions). PwC will not be given access to identifying records at these visits (i.e., signed consent forms, ASD diagnostic reports, assessment videotapes) which will be audited by members of the research team (cross-site, remote desktop audit). Approximately mid-way through data collection, a de-identified copy of raw data collected to date will be shared with the funder (JKC) to facilitate their commencement of algorithm development work. This will be with the understanding that the results of any interim analysis conducted by JKC are not disclosed to the research teams (LTU/TKI) while data collection is ongoing.

After the study is finished, a fully de-identified dataset will be shared with the funder (JKC) and third party organisation (PwC), who will work together to finalise independent analyses of the data, including algorithm development, and prepare a report (Dossier) for submission to the Therapeutic Goods Administration (TGA). JKC wish to seek TGA approval for Gazefinder in order to be able to market this as a therapeutic device in Australia in the future. No identifying information will be shared with JKC/PwC.

The sponsor will keep all data for at least 15 years after completion of the study, and archive the anonymous data file with a University Research Repository. Members of the research team may seek to use this data again in the future related projects, after seeking permission from the LTU HREC. The research team plans to present results of this trial widely – including in peer-reviewed scientific journals, at local and international conferences, and shared with the parents/families of participating children and the general community (e.g., via traditional and social media) – including jointly with JKC as appropriate. Again, no participant child’s name would be shared in such dissemination.

## Declaration of Interests

The Sponsor, LTU, has received an industry research contract and funding from JKC, the manufacturer of Gazefinder, to independently conduct this clinical trial, and will engage TKI via a Clinical Research Agreement to operate as a partner site for the conduct of this project. TKI will receive a portion of the funding provided from JKC to LTU for the conduct of the trial. JKC intends to use the results of this clinical trial to register Gazefinder with the Therapeutic Goods Administration (TGA) as a therapeutic device to aid the diagnosis of young children with autism.

The research contract and funding from JKC is not contingent on the outcome of the trial.

Funding will be provided in three instalments:

- Initial payment in January 2019 to support the first year of data collection
- Second payment in January 2020 to support the second (final) year of data collection
- Third (final) payment in April 2021 upon completion of data collection and analysis of participant characteristics, Contract Research Report by researchers to JKC.
- By agreement, members of the research team will continue collaborative discussions with JKC beyond the contract research period to support appropriate interpretation of the identified performance of the diagnostic algorithm. This is likely to include co-authoring additional documents, beyond the contract research report, that would form part of the submission by JKC to the TGA and be submitted for publication in scientific, peer-reviewed journals. Any such documentation will include clear indication of the roles, responsibilities, and potential conflict of interest of various parties.

A DMSC will be convened – independent of the research team and funding body – to review the progress and conduct of the study.

## Access to Data

The sponsor, LTU, will retain ownership of the data collected. No limits will be imposed on the researchers’ access to and use of the data, subject to HREC approval. Source and electronic data will be made available by the researchers at each site (both identifying and de-identified via secure remote desktop access) and to PwC at in-person site visits (de-identified only) – for regular audit.

Approximately mid-way through data collection, a fully de-identified dataset will be shared by the research team with the funder (JKC) to facilitate commencement of algorithm development.

A final fully de-identified dataset will be shared by the research team with JKC and PwC at the conclusion of data collection and together with the final Contract Research Report. This will allow JKC to conduct further, independent analysis of the dataset and preparation a Dossier for submission to the TGA to seek approval for promotion of Gazefinder as medical device.

## Dissemination Policy

### 27a Trial Results

The research team will seek to disseminate the results of this study widely, in scientific peer-reviewed publications, but also to other stakeholders (e.g., the public, the autistic and autism communities, government bodies, etc.) through social and traditional media outlets.

By contractual agreement, both the research teams (at LTU and TKI) and the funder (JKC) may publish and/or present the results of the trial, providing that the publication does not disclose confidential or commercially sensitive information and is not misleading to the public. Joint LTU/TKI and JKC publication and/or presentation may also be made, as appropriate, where meaningful contributions to output reflect the work of personnel at each of these organisations (e.g., source data collection by LTU/TKI researchers and algorithm development work by JKC statisticians/software engineers).

The funder, JKC, with third-party organisation PwC will prepare the results of this study in a Dossier for submission to the Therapeutic Goods Administration (TGA). The Dossier may include the report written by LTU/TKI summarising the trial (in part or in full) and/or additional documentation prepared following completion of trial data collection that falls beyond the scope of the industry research contract and funding agreement from JKC to LTU (and with Clinical Research Agreement to engage TKI as a partner site).

### 27b Authorship

Authorship on publications and outputs arising from this trial will be determined based on significant contributions to the design, conduct, and interpretation of the trial. All authors will be acknowledged as appropriate.

There are three main categories of publication that may result from the study, and the authorship team will vary accordingly:

- Reports of the main outcomes of the study – publications that report on the sensitivity/specificity of Gazefinder to different children with autism from TD children. Members of the research teams at LTU and TKI and developers of the Gazefinder technology and algorithm/s arising from the novel data collection, will be acknowledged as appropriate.
- Reports addressing one aspect of the study in detail, using data from the entire study;
- Reports of data derived from a single site or independent of the trial, but using trial data.

Any authorship disputes will be addressed by the Steering and/or Data and Safety Management Committee. The research team has no intention to employ professional medical writers.

### 27c Reproducible Research

The trial protocol will be made publicly available via registration with the ANZCTR. Following completion, a de-identified copy of the data set will be provided with a written contract research report on the trial to JKC and PwC. JKC employees with statistical expertise will conduct independent analyses to develop ‘best-fit’ diagnostic algorithm/s on the basis of the data contained with .csv source data files exported directly from the Gazefinder apparatus after each child’s assessment. These will be provided by the researchers along with each child’s group (autism vs. TD) and other participant characterisation data that have been collected. Again, algorithm development work and associated reporting by JKC toward preparing a Dossier for submission to the TGA will be the responsibility of JKC and PWC Researchers at LTU and TKI will support JKC and PWC to interpret the algorithm results, as relates to the results of the other participant characterisation data (including clinical/behavioural measures for the children with autism). And may co-author documents together that will form part of the TGA submission.

# Appendices

## Informed consent Materials

Participant Information and Consent Form attached.

## Example Source Data Exported from Gazefinder Apparatus (as .csv File)

## References

Baio, J., Wiggins, L., Christensen, D.L., et al. (2018). Prevalence of Autism Spectrum Disorder among children aged 8 years – Autism and Developmental Disabilities Monitoring Network, 11 Sites, United States, 2014. *MMWR Surveillance Summaries, 67,* 1-23. DOI: http://dx.doi.org/10.15585/mmwr.ss6706a1.

**Barbaro, J., &** **Dissanayake, C.** (2016). Diagnostic stability of Autism Spectrum Disorder in toddlers prospectively identified in a community-based setting: Behavioural characteristics and predictors of change over time. **Autism, 21,**830-840**.**

Bertenthal, B.I., Boyer, T.W. & Harding, S. (2014). When do infants begin to follow a point? *Developmental Psychology, 50,* 2036-2048.

Carter, J. V., Pan, J., Rai, S.N., & Galandiuk, S. (2016). ROC-ing along: Evaluation and interpretation of receiver operating characteristic curves. *Surgery, 59,* 1638-1645. DOI: 10.1016/j.surg.2015.12.029

Elsabbagh, M., Mercure, E., Hudry, K., et al. (2012). Infant neural sensitivity to dynamic eye gaze is associated with later emerging autism. *Current Biology, 22,* 338-342.

Fenson, L., Marchman, V.A., Thal, D.J., et al. (2007). MacArthur-Bates Communicative Development Iventories: User’s guide and technical manual (2nd ed.). Baltimore: Paul H. Brookes.

Fujioka, T., Inohara, K., Okamoto, Y., et al. (2016). Gazefinder as a clinical supplementary tool for discriminating between autism spectrum disorder and typical development in male adolescents and adults. *Molecular Autism, 7*. DOI: 10.1186/s13229-016-0083-y

Fujisawa, T.X., Tanaka, S., Saito, D.N., et al. (2014). Visual attention for social information and salivary oxytocin levels in preschool children with autism spectrum disorders: An eye-tracking study. *Frontiers in Neuroscience.* DOI: [10.3389/fnins.2014.00295](https://doi.org/10.3389/fnins.2014.00295).

Gillespie-Smith, K., Boardman, J.P., Murray, I.C., et al. (2016). Multiple measures of fixation on social content in infancy: Evidence for a single social cognitive construct. *Infancy, 21,* 241-257.

Jeste, S., & Geschwind, D. (2014). Disentangling the heterogeneity of autism spectrum disorder through genetic findings. *Nature Review of Neurology, 10,* 74–81. doi:10.1038/nrneurol.2013.278

Jones, W. & Klin, A. (2013). Attention to eyes is present but in decline in 2-6 month-olds later diagnosed with autism. *Nature, 504,* 427-431.

Kasari, C., Gulsrud, A., Paparella, T., et al. (2015). Randomized comparative efficacy study of parent-mediated interventions for toddlers with autism. *Journal of Consulting and Clinical Psychology, 83,* 554–563. doi:10.1037/a0039080

Lord, C., Risi, S., Lambrecht, L., et al. (2000). The Autism Diagnostic Observation Schedule-generic: A standard measure of social and communication deficits associated with the spectrum of autism. Journal of Autism and Developmental Disorders, 30, 205–223. doi:10.1023/ A:1005592401947

Nishizato, M., Fujisawa, T.X., Kosaka, H. & Tomoda, A. (2017). Developmental changes in social attention and oxytocin levels in infants and children. *Scientific Reports, 7*, 2540.

Oono, I., Honey, E., & McConachie, H. (2013). Parentmediated early intervention for young children with autism spectrum disorders (ASD). *Cochrane Database of Systematic Reviews.* doi:10.1002/14651858.CD009774. pub2

Rohlfing, K.J., Longo, M.R., & Bertenthal, B.I. (2012). Dynamic pointing triggers shifts of visual attention in young infants. *Developmental Science, 15*, 426-435.

Rutter, M., Bailey, A., & Lord, C. (2003). *Social Communication Questionnaire*. Los Angeles: Western Psychological Services.

Smith, T., & Iadarola, S. (2015). Evidence base update for Autism Spectrum Disorder. *Journal of Clinical Child and Adolescent Psychology, 44,* 897-922. doi:10.1080/15374416.2015.1077448

Sparrow, S.S., Cicchetti, D.V., & Balla, D.A. (2005). *Vineland Adaptive Behavior Scales (2nd ed.).* Circle Pines, MN: American Guidance Service.

Tenenbaum E.J., Shah, R.J., Sobel, D.M., et al., (2013). Increased focus on the mouth among infants in the first year of life: A longitudinal eye-tracking study. *Infancy, 18,* 534-553.

Wagner, J., Luyster, R.J., Moustapha, H., et al. (2016). Differential attention to faces in infant siblings of children with Autism Spectrum Disorder and associations with later social and language ability. *International Journal of Behavioral Development, 42,* 83-92.

# **ADDITIONAL DETAIL ON METHODS**

# **Details of *Gazefinder* Apparatus and Assessment**

Figures AF1 through 5 show details of the *Gazefinder* apparatus and guidelines for device setup and participant/viewer positioning, calibration etc. These are taken from the *Gazefinder* *Instruction Manual: Operation^1^* and *Instruction Manual: Product Specifications/Safety Precautions^2^.*

**Gazefinder apparatus description and specifications**


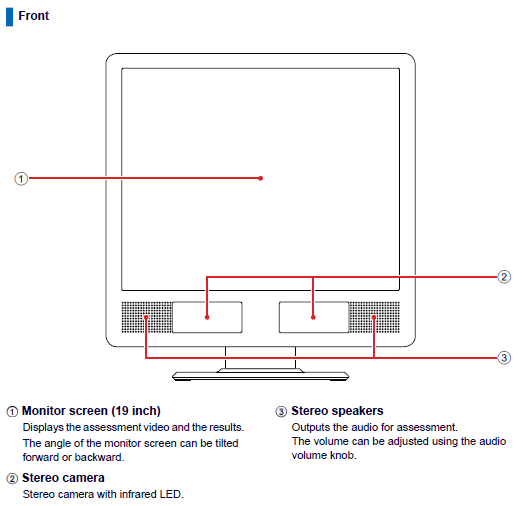

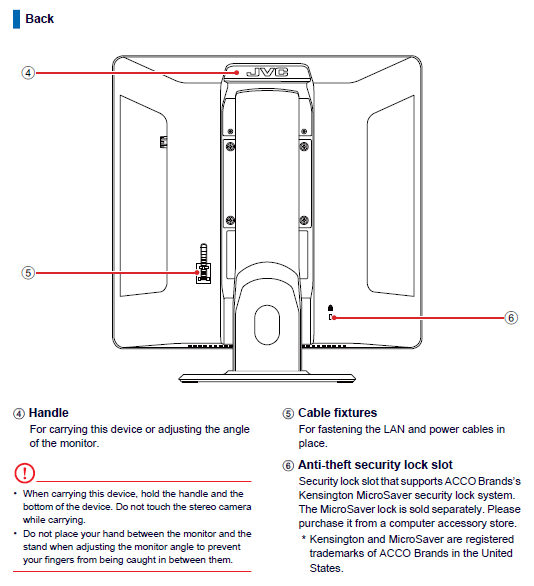

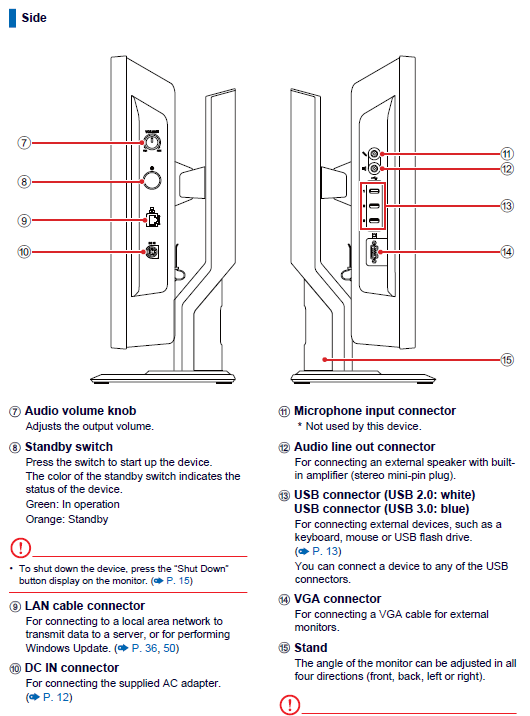


Figure AF1. Device features from product *Instruction Manual: Operation^1^* (p. 4-6).


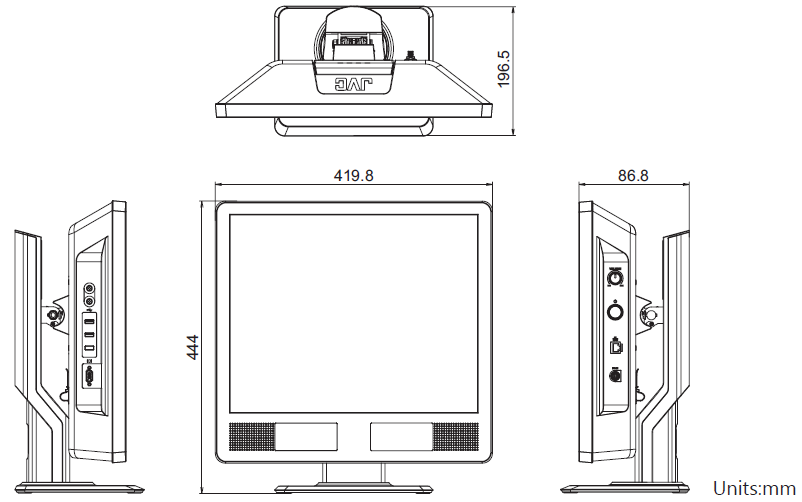

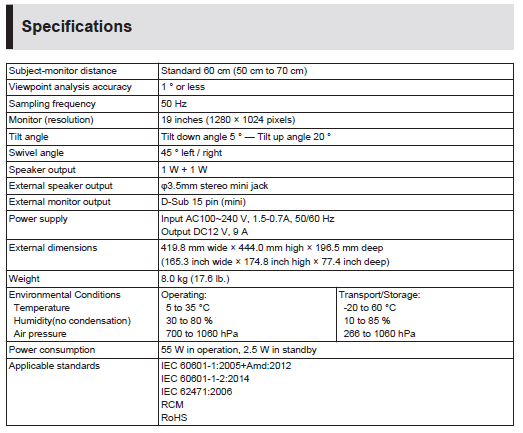
Figure AF2. Specifications from product *Instruction Manual: Product Specifications/Safety Precautions^2^* (p. 15).

**Determination of gaze fixations and movement saccades**


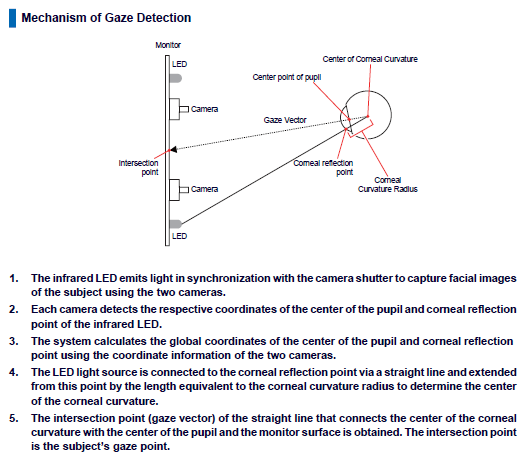

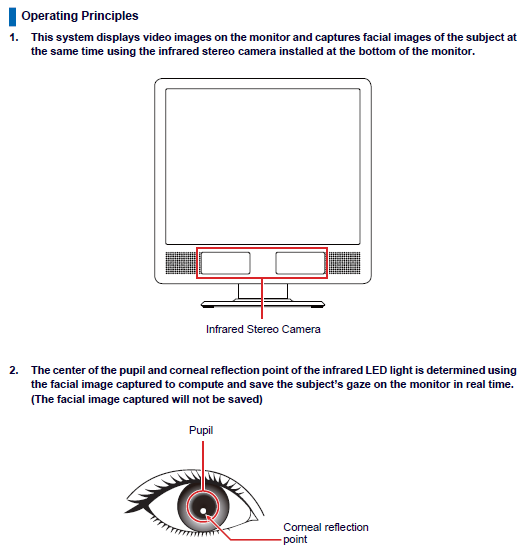


Figure AF3. Description of gaze detection technology from product *Instruction Manual: Operation^1^* (p. 51-52).

**Set-up, positioning check, and five-point calibration procedure**


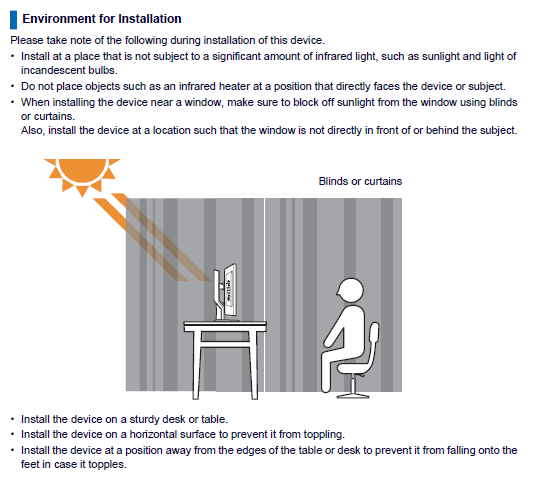

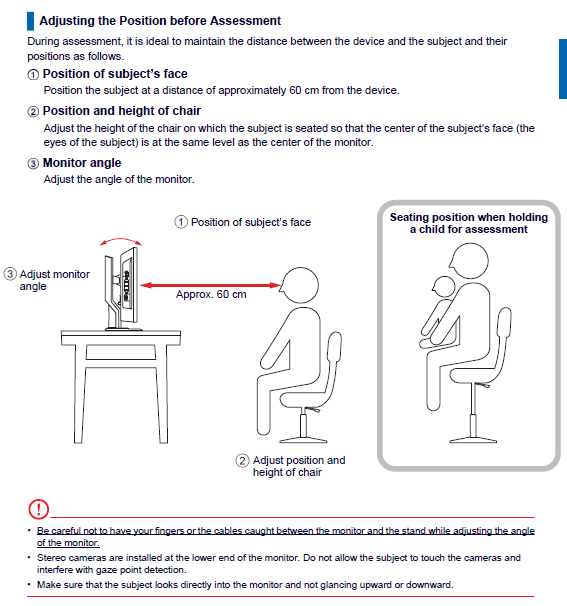


Figure AF4. Device setup and viewer positioning guidance from product *Instruction Manual: Operation^1^* (p. 10-11).

Figure AF5. Guidance for checking proper positioning and calibration from product *Instruction Manual: Operation^1^* (p. 19-20).


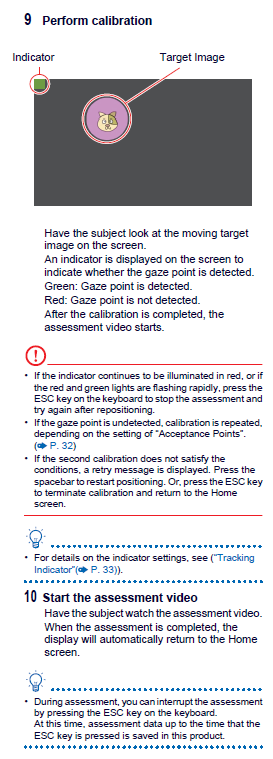

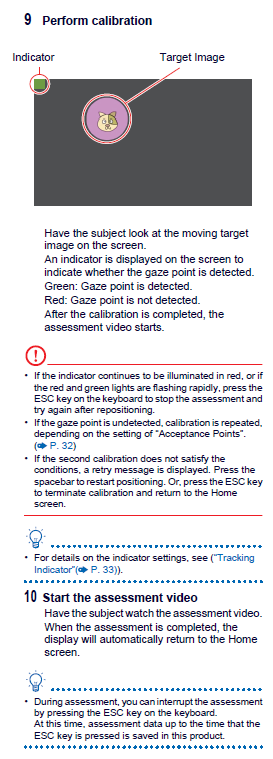

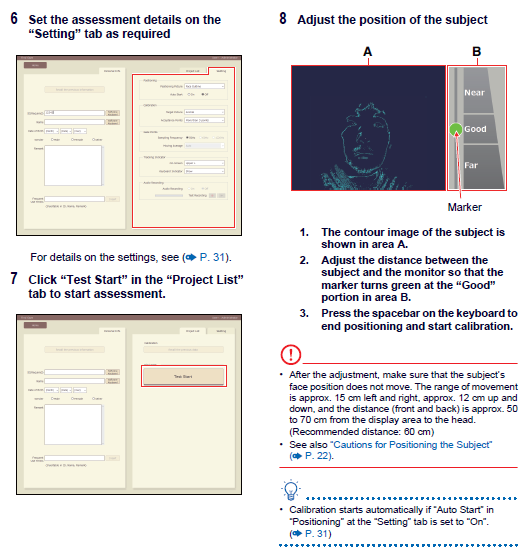


**Summary of ‘Scene 10A’ trials and pre-specified ROIs**

The main animation sequence followed immediately and automatically from successful calibration. Figure AF6 shows still images representing each of the key stimulus trials within ‘Scene 10A’, including the pre-specified ROIs as possible candidates for inclusion in the final classification algorithm. Gaze fixation data were easily exported from the Gazefinder device as an encrypted .csv file per participant. Along with the overall tracking rate (i.e., proportion of animation with any gaze data recorded, key trials reflected four stimulus types as follows:

- *People* vs. *Geometry*: Total of 10 trials targeting preferential attention to social vs. non-social stimuli/scenes, including six trials with paired equally-sized stimuli, two trials whereby geometric patterns were embedded within larger social scenes; and two trials with four equally-sized stimuli (two each social and non-social), all counterbalanced for relative position of social vs. non-social elements on screen;
- *Eyes* vs. *mouth* of a human face: Total of five trials of the same face, varying in terms of static/dynamic features, and with or without sound (i.e., blinking, mouth moving [silently]; still face [two trials, separated by other stimulus trials]; and talking);
- *Response to joint attention* type probes: Two trials each including a *target* and two *distractor* objects, and a *referential agent* (in one case showing pointing hand only; and in the other, showing the agent’s full torso); and
- Biological motion point-light display (PLD) type animations: Two trials reflecting paired *upright* and *inverted* PLD-type stimuli (counterbalanced for side of screen). Note that while these two trials were maintained in the animation for consistency with past published work using Scene 10,^3^ no ROIs for these stimuli were considered for potential inclusion in the classification algorithm as past work has shown no inconsistent potential for these to differentiate people with vs. without autism.^3-6^


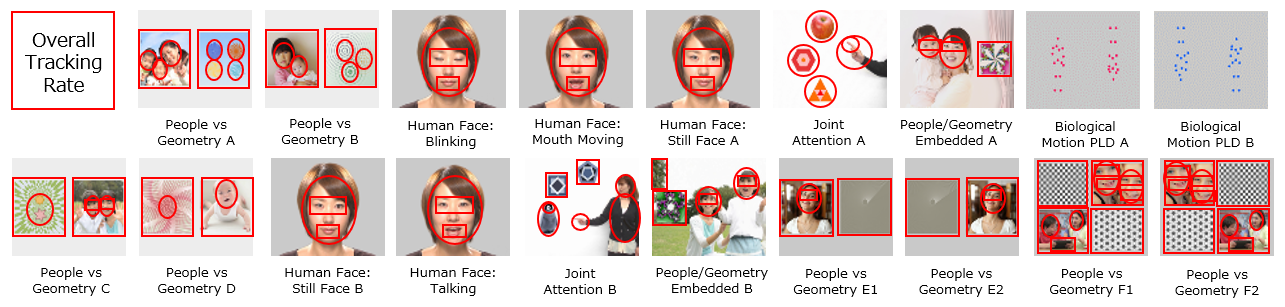


Figure AF6. Key stimuli within ‘Scene 10A’ showing all pre-specified Regions of Interest (ROIs) among which candidates were evaluated for inclusion within final Classification Algorithm.

Table AF1. Detailed specifications of key stimuli within ‘Scene 10A’

| **Order** | **Label** | | | **Onset Time (ms)** | **Duration (ms)** | **Thumbnail Image** | **Description** |
| --- | --- | --- | --- | --- | --- | --- | --- |
|  | **Trial** | **Segment** | |  |  |  |  |
|  |  | |  | 0 | 5,000 | Attention-grabbing animation | |
| 1 | Pref-A | | A1 | 5,000 | 1,000 | 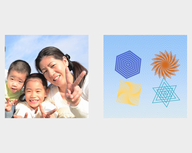 | Matched-size social/non-social scenes: People (left) vs. Geometry (right) with fade-in effect for People (Seg. A1) and fade-out for Geometry (Seg. A2). |
| 2 |  |  | A2 | 6,000 | 4,000 |  |  |
| 3 | Pref-B | | B1 | 10,000 | 1,000 | 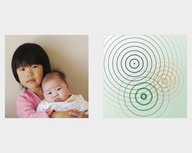 | Matched-size social/non-social scenes: People (left) vs. Geometry (right) with fade-in effect for Geometry (Seg. A1) and fade-out for People (Seg. A2). |
| 4 |  |  | B2 | 11,000 | 4,000 |  |  |
|  |  | |  | 15,000 | 2,000 | 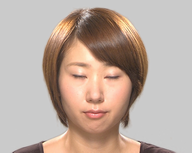Attention-grabbing animation | |
| 5 | Blink | |  | 17,000 | 5,000 |  | Animation showing woman:  Blinking  Moving mouth (silently)  Still face (static/silent) |
| 6 | Mouth | |  | 22,000 | 2,000 | 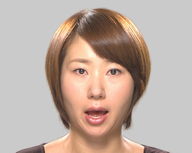 |  |
| 7 | Still-A | |  | 27,000 | 5,000 | 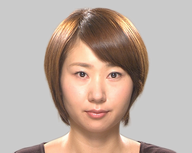 |  |
|  |  | |  | 32,000 | 2,000 | Attention-grabbing animation | |
| 8 | Point-A | | A1 | 34,000 | 1,000 |  | Finger pointing to one of three items, and *"What is this?"* (in English) with fade-in effect for objects (Seg. A1; clearly visible Seg. A2). |
| 9 |  |  | A2 | 35,000 | 3,000 | 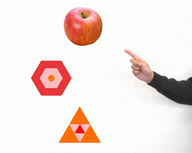 |  |
|  |  | |  | 38,000 | 2,000 | Attention-grabbing animation | |
| 10 | Win-A | | A1 | 40,000 | 3,000 | 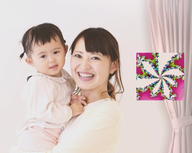 | Social/non-social scenes: whereby People (larger) and Geometry (smaller, embedded). Geometry animation stronger (Seg. A1) then reduced (Seg. A2). |
| 11 |  |  | A2 | 43,000 | 5,000 |  |  |
|  |  | |  | 48,000 | 4,000 | Attention-grabbing animation | |
| 12 | BiolMot-A | | | 52,000 | 5,000 | 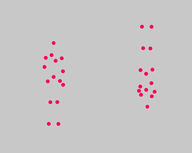 | Biological motion Point-Light Displays (PLD):  BiolMot-A figures Upright (left) and Inverted (right)  BiolMot-B figures Upright (right) and Inverted (left) |
| 13 | BiolMot-B | | | 57,000 | 6,000 | 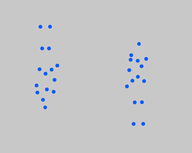 |  |
|  |  | |  | 63,000 | 2,000 | Attention-grabbing animation | |
| 14 | Pref-C | | C1 | 65,000 | 1,000 |  | Matched-size social/non-social scenes: People (right) vs. Geometry (left) with fade-in effect for People (Seg. A1) and fade-out for Geometry (Seg. A2). |
| 15 |  |  | C2 | 66,000 | 4,000 | 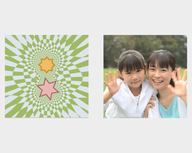 |  |
| 16 | Pref-D | | D1 | 70,000 | 1,000 |  | Matched-size social/non-social scenes: People (right) vs. Geometry (left) with fade-in effect for Geometry (Seg. A1) and fade-out for People (Seg. A2). |
| 17 |  |  | D2 | 71,000 | 4,000 | 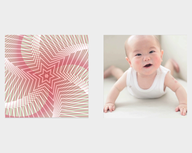 |  |
|  |  | |  | 75,000 | 5,000 | 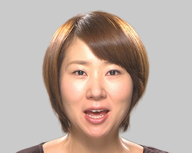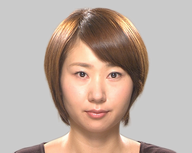Attention-grabbing animation | |
| 18 | Still-B | |  | 80,000 | 4,000 |  | Animation showing woman:  Still face (static/silent)  Talking: *“Hello. What is your name? Let’s play.”* (in Japanese) |
| 19 | Talk | |  | 84,000 | 7,000 |  |  |
|  |  | |  | 91,000 | 2,000 | Attention-grabbing animation | |
| 20 | Point-B | | B1 | 93,000 | 1,000 | 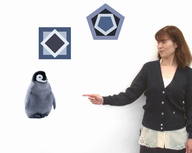 | Person pointing to one of three items, and *"What is this?"* (in English) with fade-in effect for objects (Seg. A1; clearly visible Seg. A2). |
| 21 |  |  | B2 | 94,000 | 3,000 |  |  |
|  |  | |  | 97,000 | 2,000 | Attention-grabbing animation | |
| 22 | Win-B | | B1 | 99,000 | 3,000 | 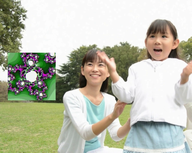 | Social/non-social scenes: whereby People (larger) vs. Geometry (smaller, embedded). Geometry animation stronger (Seg. A1) then reduced (Seg. A2). |
| 23 |  |  | B2 | 102,000 | 5,000 |  |  |
|  |  | |  | 107,000 | 2,000 | Attention-grabbing animation | |
| 24 | Pref-E | | E1 | 109,000 | 6,000 | 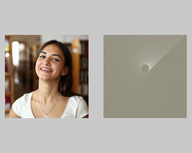 | Matched-size social/non-social scenes:  Segment E1 with People (left) vs. Geometry (right)  Segment E2 with People (right) vs. Geometry (left) |
| 25 |  |  | E2 | 115,000 | 6,000 | 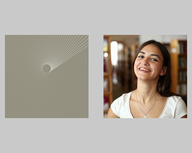 |  |
|  |  | |  | 121,000 | 1,000 | Attention-grabbing animation | |
| 26 | Pref-F | | F1 | 122,000 | 5,000 | 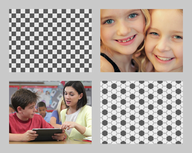 | Matched-size social/non-social scenes (quadrants):  Seg. F1 People (top right/ bottom left); Geometry (top left/bottom right)  Seg. F2 People (top left/ bottom right); Geometry (top right/bottom left) |
| 27 |  |  | F2 | 127,000 | 5,000 | 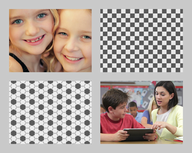 |  |

**Detailed ROI parameters**

Along with the overall gaze tracking rate, gaze fixation data to 18 ROIs—across 14 of the 19 available stimulus trials—were retained within the final classification algorithm (summarised in Figure AF7). As detailed in Table AF2 (below), these were rectangular/square or circular/oval-shaped, specified by x- and y-axis points on the device monitor. Where stimulus/trial onset followed immediately from an attention-grabbing animation, the ROI onset was equal to the stimulus/trial onset time. Where stimulus/trial onset followed on from another stimulus/trial (i.e., no immediately-preceding attention-grabbing animation), ROI onset timing was delayed by 0.3 seconds after stimulus onset, to counter potential carry-over of gaze fixation on the previously-presented stimulus. Details of attention-grabbing animation elements and stimulus/trial onset and duration times are included in Table AF1 (above).


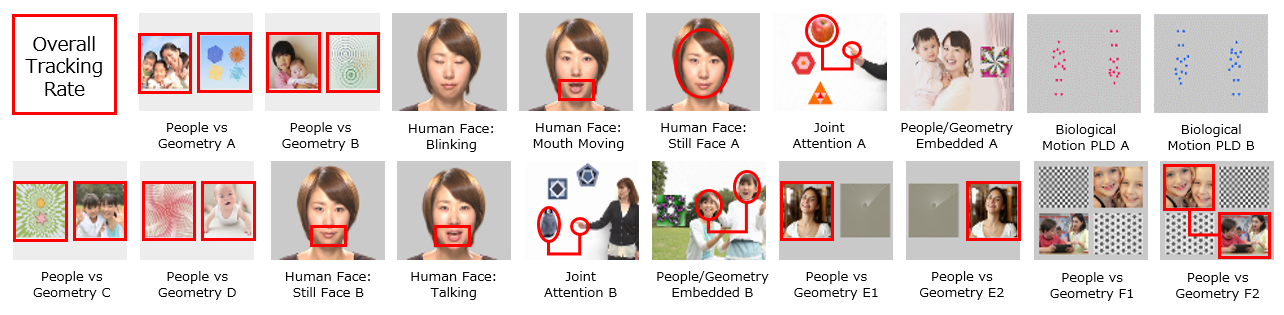


Figure AF7. Final set of ROIs retained for classification algorithm, within key ‘Scene 10A’ trials.

Table AF2. Detailed specifications of ‘Scene 10A’ ROIs retained for classification algorithm

| **ROI #** | **Trial/Segment Label** | **Onset Time (ms)** | **Stimulus/ROI Duration (ms)** | **ROI Shape** | **ROI Parameters (pixels)** | | | | | **Thumbnail Image** | **ROI Content Summary** |
| --- | --- | --- | --- | --- | --- | --- | --- | --- | --- | --- | --- |
|  |  |  |  |  | **x-axis** | **y-axis** | **width** | | **height** |  |  |
| 1 | Pref-A | 5,000 | 5,000 | Two Squares | 32 | 234 | 560 | 560 | | 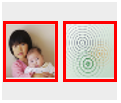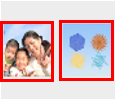 | People Panel |
| 2 |  |  |  |  | 690 | 234 | 560 | 560 | |  | Geometry Panel |
| 3 | Pref-B | 10,300 | 4,700 | Two Squares | 32 | 234 | 560 | 560 | |  | People Panel |
| 4 |  |  |  |  | 690 | 234 | 560 | 560 | |  | Geometry Panel |
| 5 | Mouth | 22,000 | 2,000 | Rectangle | 543 | 702 | 202 | 110 | | 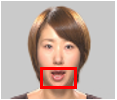 | Mouth Region |
| 6 | Still-A | 27,000 | 5,000 | Oval | 430 | 370 | 436 | 472 | | 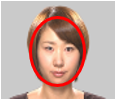 | Whole Face Region |
| 7 | Point-A | 34,000 | 4,000 | Combination of Two Ovals | 400 | 20 | 303 | 313 | | 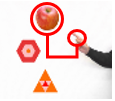 | Target Object + Pointing Finger |
|  |  |  |  |  | 789 | 266 | 126 | 131 | |  |  |
| 8 | Pref-C | 65,000 | 5,000 | Two Squares | 32 | 234 | 560 | 560 | | 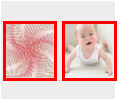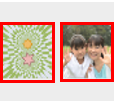 | Geometry Panel |
| 9 |  |  |  |  | 690 | 234 | 560 | 560 | |  | People Panel |
| 10 | Pref-D | 70,300 | 4,700 | Two Squares | 32 | 234 | 560 | 560 | |  | Geometry Panel |
| 11 |  |  |  |  | 690 | 234 | 560 | 560 | |  | People Panel |
| 12 | Still-B | 80,000 | 4,000 | Rectangle | 543 | 702 | 202 | 110 | | 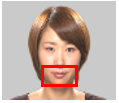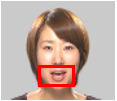 | Mouth Region |
| 13 | Talk | 84,000 | 7,000 | Rectangle | 543 | 702 | 202 | 110 | |  | Mouth Region |
| 14 | Point-B | 93,000 | 2,000 | Combination of Oval + Circle | 122 | 448 | 285 | 383 | | 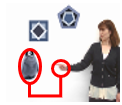 | Target Object + Pointing Finger |
|  |  |  |  |  | 497 | 550 | 232 | 232 | |  |  |
| 15 | Win-B | 99,000 | 2,000 | Combination of Two Ovals | 486 | 311 | 259 | 273 | | 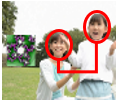 | Mother’s + Child’s Faces |
|  |  |  |  |  | 878 | 37 | 330 | 350 | |  |  |
| 16 | Pref-E1 | 109,000 | 6,000 | Rectangle | 32 | 234 | 560 | 560 | | 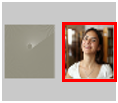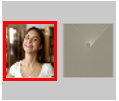 | People Panel |
| 17 | Pref-E2 | 115,300 | 5,700 | Rectangle | 690 | 234 | 560 | 560 | |  | People Panel |
| 18 | Pref-F2 | 127,300 | 4,700 | Combination of Two Rectangles | 56 | 56 | 560 | 430 | | 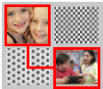 | People (top left, bottom right) |
|  |  |  |  |  | 668 | 540 | 560 | 430 | |  |  |

Note. For ROI Parameters, origin of x- and y- axes is top-left corner of screen, and width and height parameters for circles/ovals representing maximal points.

# **Details of Clinical Characterisation Measures**

The **Social Communication Questionnaire** (SCQ)^7^ is a caregiver-report measure of behaviours relevant to an ASD diagnosis. The ‘Current’ version was used here, with caregivers reporting on their child’s current/recent behavioural presentation, across 40 yes/no items. The summary SCQ Total Score was computed as usual, such that higher scores reflect more behavioural autism features. For this study, the SCQ was used as an eligibility screen for children in the Non-Autism Group (i.e., ineligible if score >12, suggesting possible subthreshold autism features) and as a continuous measure of caregiver-reported autism features for children in the Autism Group.

The **Vineland Adaptive Behaviour Scales***–2nd edition (VABS-2)*^8^ is a caregiver-report measure of everyday functional skills with questions covering items that converge across subdomains within four overarching domains—Communication, Socialization, Daily Living, and Motor Skills. From these, the overall Adaptive Behaviour Composite (ABC) was computed (a norm-referenced Standard Score; population *M*=100; *SD*=15) with higher scores reflecting greater skills relative to age-related expectations. Caregivers of children in both groups completed the *VABS-2* for a measure of child adaptive functioning which evidenced substantial heterogeneity within the Autism Group and broadly age-expected adaptive behaviour for children in the Non-Autism Group.

The **Child Behavior Checklist** (CBCL)^9^ is a caregiver-report measure of child behaviour difficulties with 100 items informing DSM-5 criteria for Depression, Anxiety, Autism Spectrum Disorder (ASD), Attention Deficit/Hyperactivity Disorder (ADHD), and Oppositional Defiant Disorder (ODD). The pre-school version for children aged 1½–5½ years was used, with norm-referenced *t*-scores computed (population *M*=50; *SD*=10) where higher scores reflect greater difficulties. Caregivers of children in both groups completed the CBCL for a measure of potential internalising and externalising difficulties which served to confirm unlikely clinical/subthreshold autism features or other substantive conditions among children in the Non-Autism Group, and high-level autism features with increased rates of internalising/externalising features for children in the Autism Group.

The **Autism Diagnostic Observation Schedule***—2^nd^ Edition* (ADOS-2)^10, 11^ was administered with each autistic child as a measure of behavioural autism features. The ADOS-2 is a play-based assessment developed to elicit behaviours relevant to an ASD diagnosis in children aged 12-30 months (Toddler Module) or 31+ months (Modules 1 to 3 here; Module 4 for adolescents/adults) and used internationally in research, and in clinical practice as part of ASD diagnostic assessment. The relevant module was administered given each child’s age and expressive language abilities, with Social Affect and Restricted Repetitive Behaviour domain and Total Algorithm Scores computed as usual, with higher scores reflecting more behavioural autism features. We also derived a Calibrated Severity Score (CSS)^12^ for each child, ranging plausibly from 1-10 (again, higher score reflecting more behavioural autism features), for greater cross-module comparability. ADOS-2 assessment provided a continuous measure of behavioural autism features and served to corroborate the community diagnoses of children included in the Autism Group.

ADOS-2 assessments were conducted and scored by researchers with research-level training and reliability in administration and coding, on both the core set of Modules 1-4 and the specialist Toddler Module. The clinical trial team also included individuals who are recognised and experienced trainers on the ADOS-2. Formal appraisal of within-trial inter-rater agreement was conducted by having a subset of 15 ADOS-2 assessments (broadly stratified by Module) re-coded from video by a second rater who had not administered the given assessment. Inter-rater reliability on summary algorithm scores computed from each rater’s item-level codes was very good for the Social Affect domain (Intra-Class Correlation [ICC] = .77), modest for the Restricted Repetitive Behaviour domain (ICC = .51), and very good for the Total Algorithm Score (ICC = .73) and the summary CSS (ICC = .79).

The **Mullen Scales of Early Learning** *(MSEL)*^13^ is a researcher-administered developmental assessment of early motor and cognitive development for children aged 0-68 months. Tasks span four primary scales—Visual Reception and Fine Motor skills (non-verbal domains), and Receptive and Expressive Language skills (verbal domains)—yielding an Early Learning Composite (ELC) Standard Score (population *M*=100; *SD*=15) where higher scores reflect greater skills relative to age-related expectations. We also computed Non-Verbal and Verbal Developmental Quotients: the average of relevant scale age-equivalent scores divided by child’s chronological age and multiplied by 100. The MSEL was administered with children in the Autism Group only, for formal assessment of developmental/cognitive abilities which are known to vary in this population, from very low to within- and even well above the average level for a given individual’s age.

The **MacArthur-Bates Communicative Development Inventories** *(MCDI)*^14^ is a caregiver-report measure of emerging early vocabulary and communicative gesture use. We provided either the MCDI Words and Gestures (WG) or Words and Sentences (WS) form for each participant in the Autism Group, as relevant to their current language level. Caregivers endorse the number of words the child ‘understands’ or both ‘understands and says’ among an inventory spanning different semantic categories (WG maximum = 396 words; WS maximum = 680). To achieve a common, comparable set of outcome measures for all children—irrespective of broad language level—we slightly modified the WS form so that caregivers were asked to endorse both words the child ‘understands’ or ‘understands and says’ (vs. the latter only, in the originally developed version) and appended the section pertaining to communicative gesture use (originally only in the WG form). Hence, for each child with autism, we derived a Receptive Vocabulary Count (combined total of all items endorsed ‘understands’ or ‘understands and says’), an Expressive Vocabulary Count (total of all items endorsed ‘understands and says’), and a Total Gestures score.

# **Additional Detail on Statistical Method**

# **Algorithm Development Analysis**

Each child’s Total Score on the *Gazefinder* classification algorithm was calculated as the sum of 18 retained ROIs, plus overall gaze tracking rate, standardised (for sample mean and standard deviation) for a possible range from 0-100, as shown in the equation below. Performance properties of this algorithm for case-control classification were calculated with a threshold score of 28.6 (score above threshold indicating likely autism-group membership; below threshold indicating likely Non-Autism Group membership).


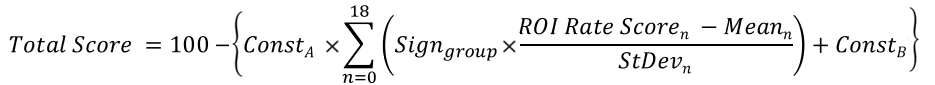


Table AF3. Description of parameters within equation for Gazefinder classification algorithm

| **Parameter** | **Description** |
| --- | --- |
| ROI Rate Score_n_ | ROI Rate Score_0_ 🡪 Overall Tracking Rate  ROI Rate Score_1-18_ n 🡪 Score for each of 18 retained ROIs from Scene 10A trials |
| Mean_n_ | Mean of sample ROI Rate Scores |
| StDev_n_ | Standard deviation of sample ROI Rate Scores |
| Sign_group_ | Sign_ASD_ 🡪 -1  Sign_TD._ 🡪 +1 |
| Const_A_ | Constant parameter to set all Gazefinder Total Scores within the range 0-100 |
| Const_B_ | Constant parameter to set all Gazefinder Total Scores within the range 0-100 |

# **STROBE REPORTING CHECKLIST FOR CASE-CONTROL STUDIES**

|  | Item No | Recommendation | Page |
| --- | --- | --- | --- |
| **Title and abstract** | 1 | (*a*) Indicate the study design with a commonly used term in title or abstract | 3 |
|  |  | (*b*) Provide in the abstract an informative and balanced summary of what was done and what was found | 3-4 |
| Introduction | | | |
| Background/rationale | 2 | Explain the scientific background and rationale for the investigation being reported | 5-6 |
| Objectives | 3 | State specific objectives, including any prespecified hypotheses | 6 |
| Methods | | | |
| Study design | 4 | Present key elements of study design early in the paper | 7 |
| Setting | 5 | Describe the setting, locations, and relevant dates, including periods of recruitment, exposure, follow-up, and data collection | 7-12 |
| Participants | 6 | (*a*) Give the eligibility criteria, and the sources and methods of case ascertainment and control selection. Give the rationale for the choice of cases and controls | 7-8 |
|  |  | (*b*) For matched studies, give matching criteria and the number of controls per case | N/A |
| Variables | 7 | Clearly define all outcomes, exposures, predictors, potential confounders, and effect modifiers. Give diagnostic criteria, if applicable | 8-11 |
| Data sources/ measurement | 8* | For each variable of interest, give sources of data and details of methods of assessment (measurement). Describe comparability of assessment methods if there is more than one group | 8-11 |
| Bias | 9 | Describe any efforts to address potential sources of bias | 8, 10-12 |
| Study size | 10 | Explain how the study size was arrived at | 8 |
| Quantitative variables | 11 | Explain how quantitative variables were handled in the analyses. If applicable, describe which groupings were chosen and why | 10-12 |
| Statistical methods | 12 | (*a*) Describe all statistical methods, including those used to control for confounding | 12 |
|  |  | (*b*) Describe methods used to examine subgroups and interactions | 12 |
|  |  | (*c*) Explain how missing data were addressed | 12-13 |
|  |  | (*d*) If applicable, explain how matching of cases and controls was addressed | N/A |
|  |  | (*e*) Describe any sensitivity analyses | 16, 18 |
| Results | | | |
| Participants | 13* | (a) Report numbers of individuals at each stage of study—eg numbers potentially eligible, examined for eligibility, confirmed eligible, included in the study, completing follow-up, and analysed | 12, 13, 15-16 |
|  |  | (b) Give reasons for non-participation at each stage |  |
|  |  | (c) Consider use of a flow diagram | Figure 2 |
| Descriptive data | 14* | (a) Give characteristics of study participants (eg demographic, clinical, social) and information on exposures and potential confounders | 14-15,  Table 1-2 |
|  |  | (b) Indicate number of participants with missing data for each variable of interest | Table 1-2 |
| Outcome data | 15* | Report numbers in each exposure category, or summary measures of exposure | 13,  Figure 2 |
| Main results | 16 | (*a*) Give unadjusted estimates and, if applicable, confounder-adjusted estimates and their precision (e.g., 95% confidence interval). Make clear which confounders were adjusted for and why they were included | 18, Figure 4 |
|  |  | (*b*) Report category boundaries when continuous variables were categorized |  |
|  |  | (*c*) If relevant, consider translating estimates of relative risk into absolute risk for a meaningful time period | N/A |
| Other analyses | 17 | Report other analyses done—eg analyses of subgroups and interactions, and sensitivity analyses | 18-19, Table 3, Add’l File |
| Discussion | | | |
| Key results | 18 | Summarise key results with reference to study objectives | 20 |
| Limitations | 19 | Discuss limitations of the study, taking into account sources of potential bias or imprecision. Discuss both direction and magnitude of any potential bias | 22-25 |
| Interpretation | 20 | Give a cautious overall interpretation of results considering objectives, limitations, multiplicity of analyses, results from similar studies, and other relevant evidence | 20-22 |
| Generalisability | 21 | Discuss the generalisability (external validity) of the study results | 21-25 |
| Other information | | | |
| Funding | 22 | Give the source of funding and the role of the funders for the present study and, if applicable, for the original study on which the present article is based | 1, 27 |

*Give information separately for cases and controls.

# **ADDITIONAL RESULTS**

# **Detailed Sample Characterisation**

Table AF4. Personal and familial socio-demographic characteristics of eligible children

|  | | | | | **Autism** | **Non-Autism** | **Between-Group Difference** |
| --- | --- | --- | --- | --- | --- | --- | --- |
|  | | | | | **(n = 102)** | **(n = 101)** |  |
| Age in months: Mean (SD) range | | | | | 44 (8.8) 24-60 | 40 (10.5) 24-60 | *U*=6308.5, *p=*.006, r_rb_=.23 |
| Sex at Birth: n male/female | | | | | 83/19 | 58/43 | *χ^2^*(1)=13.72, *p*<.001 |
| Gestational Age at Birth | | | Full Term | | 88 | 93 | *ꭓ*^2^(1)=0.13, *p*=.716^a^ |
|  | | | 32-37 weeks | | 6 | 6 |  |
|  | | | <32 weeks | | 1 | 0 |  |
| Missing/Not Disclosed | | | | | 7 | 2 |  |
| Birth Order among Siblings | | | | Only Child | 26 | 33 | *ꭓ*^2^(2)=1.05, *p*=.592 |
|  | | | First Born | | 30 | 26 |  |
|  | | | Later Born | | 40 | 41 |  |
| Missing/Not Disclosed | | | | | 6 | 1 |  |
| Ethnicity Caucasian Australian | | | | | 59 | 84 | *ꭓ*^2^(1)=12.58, *p*<.001^a^ |
| Indigenous Australian | | | | | 4 | 0 |  |
| North-East Asian | | | | | 4 | 2 |  |
| South-East Asian | | | | | 3 | 0 |  |
| Mixed Ethnicity | | | | | 14 | 11 |  |
| Other Ethnicity | | | | | 9 | 1 |  |
| Missing/Not Disclosed | | | | | 9 | 3 |  |
| Family Composition | | | Dual Parent | | 81 | 98 | *ꭓ*^2^(1)=11.48, *p<*.001^a^ |
|  | | | Single Parent | | 12 | 2 |  |
|  | | | Other Adults | | 3 | 0 |  |
| Missing/Not Disclosed | | | | | 6 | 1 |  |
| Nominated Primary Carer | | | Mother | | 90 | 93 | *ꭓ*^2^(1)=0.27, *p*=.601^a^ |
|  | | | Father | | 3 | 7 |  |
|  | | | Grandparent | | 3 | 0 |  |
| Missing/Not Disclosed | | | | | 6 | 1 |  |
| Primary Carer Education | | | Primary School | | 1 | 0 | *ꭓ*^2^(2)=19.97, *p*<.001^b^ |
|  | Secondary (15-16y) | | | | 12 | 2 |  |
|  | Secondary (17-18y) | | | | 10 | 3 |  |
|  | | | Degree | | 34 | 29 |  |
|  | | | Post-Graduate | | 37 | 66 |  |
| Missing/Not Disclosed | | | | | 8 | 1 |  |
| Primary Home Language | | | English | | 89 | 97 | *ꭓ*^2^(1)=1.86, *p*=.172 |
|  | | | Other | | 7 | 3 |  |
| Missing/Not Disclosed | | | | | 6 | 1 |  |
| Household Income | | <AUD$25,000 | | | 6 | 0 | *ꭓ*^2^(4)=25.08, *p<.*001 |
|  | | AUD$25-50,000 | | | 12 | 3 |  |
|  | | AUD$50-85,000 | | | 12 | 5 |  |
|  | | AUD$85-115,000 | | | 19 | 24 |  |
|  | | >AUD$115,000 | | | 31 | 64 |  |
| Missing/Not Disclosed | | | | | 22 | 5 |  |

*Note.* ^a^ Two-category comparisons (full term vs. any pre-term birth; Caucasian Australian vs. any other ethnicity group; dual parent vs. single parent/other adults; mother vs. father). ^b^ Three-category comparison (any primary/secondary vs. degree vs. post-graduate education).

Table AF5. Available data on clinical phenotyping measures for all eligible children

|  | **Autism Group** | | **Non-Autism Group** | | **Between-Group Difference** |
| --- | --- | --- | --- | --- | --- |
|  | **n** | **M (SD) range** | **n** | **M (SD) range** |  |
| SCQ Total Score | 94 | 19.32 (6.58) 5-38 | 101 | 3.56 (2.50) 0-10 | *U*=9390.5, *p*<.001, *r_rb_*=.98 |
| VABS ABC SS | 93 | 75.0 (12.4) 50-108 | 93 | 111.2 (13.5) 80-139 | *t*(178)=-19.1, *p*<.001, *d*=-2.80 |
| CBCL T-Score | 85 |  | 98 |  |  |
| Depression |  | 64.89 (10.58) 50-95 |  | 53.07 (4.78) 50-70 | *U*=7293.5, *p*<.001, *r_rb_*=.75 |
| Anxiety |  | 61.21 (11.23) 50-90 |  | 52.22 (4.97) 50-85 | *U*=6462.0, *p*<.001, *r_rb_*=.55 |
| Autism Spectrum |  | 74.28 (9.04) 50-94 |  | 51.27 (2.69) 50-64 | *U*=8205.5, *p*<.001, *r_rb_*=.97 |
| ADHD |  | 61.91 (8.44) 50-76 |  | 51.98 (4.27) 50-76 | *U*=7236.5, *p*<.001, *r_rb_*=.74 |
| Oppositionality |  | 59.75 (9.27) 50-80 |  | 52.47 (4.40) 50-73 | *U*=6363.0, *p*<.001, *r_rb_*=.53 |
| ADOS-2 | 100 |  |  |  |  |
| Overall CSS |  | 7.56 (1.62) 3-10 | - | - | - |
| Social Affect |  | 12.27 (4.03) 3-20 | - | - | - |
| Restricted/Repet. |  | 5.07 (1.86) 1-8 | - | - | - |
| MSEL | 101 |  |  |  |  |
| ELC SS | (98) | 65.53 (21.05) 49-134 | - | - | - |
| Non-Verbal DQ |  | 68.56 (22.01) 20-121 | - | - | - |
| Verbal DQ | (99) | 59.73 (30.14) 13-137 | - | - | - |
| MCDI | 82 |  |  |  |  |
| Receptive Vocab. | (81) | 354.5 (210.0) 6-678 | - | - | - |
| Expressive Vocab. | (81) | 271.9 (228.4) 0-678 | - | - | - |
| Total Gestures |  | 39.18 (14.69) 0-61 | - | - | - |

*Note.* SCQ = Social Communication Questionnaire^7^; VABS ABC SS = Vineland Adaptive Behavior Scales-2^nd^ edition^8^ Adaptive Behaviour Composite Standard Score; CBCL = Child Behaviour Checklist^9^; ADOS-2 CSS = Autism Diagnostic Observation Schedule – 2nd Edition^10, 11^ Calibrated Severity Score^15^; MSEL = Mullen Scales of Early Learning^13^; ELC SS = Early Learning Composite Standard Score; DQ = Developmental Quotient; MCDI = McArthur-Bates Communicative Development Inventories^14^.

**Within-Group Heterogeneity and Between-Group Comparability**

***Personal and Family Socio-Demographic Characteristics.*** As summarised in Table 1 of the main manuscript, autistic children were slightly but significantly older as a group than non-autistic children (M_diff_ 4 months; *t*[201]=2.95, *p=*.004, *d*=.41). Autistic children were also significantly more likely to be male than female, at 4.37:1 ratio (consistent with recent estimate from meta-analysis of 4.56:1 [95%*CI* 4.1:1, 5.07:1] autism sex imbalance^16^). Autistic and non-autistic children did not differ in terms of parity (i.e., similar numbers only children, and first- vs. later-born among siblings) and numbers born pre-term within each group were broadly consistent with national data (8.7%^17^ born <37 weeks’ gestational age with one single case very pre-term at ≤32 weeks’). From self-reported parental ethnicities, we determined most children to be Australian of European or non-specified ethnic descent, but with relatively greater diversity among the Autism vs. Non-Autism Groups. In the Autism Group *only* were a small number of children whose parent identified as Indigenous Australian, broadly consistent with the Australian Bureau of Statistics (ABS)^18^ 3% rate.

Compared to with the Non-Autism Group, children in the Autism Group were more often from single-parent households (but below the estimated 22% national rate^19^). Three autistic children were from households including adults other than biological parent/s (including one child living with both grandparents, and two living with mother and grandmother; locally, often reflecting contexts of family cultural/linguistic diversity^19^). English was nominated primary home language for most families, with other languages including Chinese, Russian and several South Asian languages for cases, and Chinese, Persian and Portuguese for controls. Mothers were the most frequently nominated primary caregiver for children in both groups, but the highest level of caregiver completed education differed such that relatively more primary caregivers of autistic children were educated only to secondary level (age 17/18 years) with a sizeable subgroup here not educated beyond age 15/16 years. Among both groups, however, caregiver education exceeded national average rates.^20^ Where parents disclosed household income, this varied across the cohort but was significantly lower for autistic than non-autistic children, with only half as many autistic vs. non-autistic children from families with very high income (and est. 11 autistic and two non-autistic children living below the poverty line; AUD$40,000 for a family of four^21^).

***Child Clinical/Behavioural Characterisation.*** As summarised in Table 2 of the main manuscript, group mean-level SCQ scores were significantly higher for autistic than non-autistic children, with scores for the latter mostly all >12 (i.e., eligibility threshold set for Non-Autism Group; *n*=80) but varying across almost the full possible range of scores, from relatively subtle social-communication features for some through more pronounced features for others. ADOS-2 direct assessment scores corroborated the suggestion of a heterogeneous Autism Group, with a range of modules administered given varied child ages and expressive language abilities (Toddler n=9; Module 1 n=50; Module 2 n=36; Module 3 n=5), and range of scores across *Social Affect* and *Restricted Repetitive Behaviour* domains Algorithm Totals and the summary Calibrated Severity Score. Children in the Autism Group had Algorithm Total Scores meeting ADOS-2 threshold for either more pronounced *Autism* (*n*=87) or broader *Autism Spectrum* (*n*=12) presentation with one exception (a 3-year-old old girl assessed with Module 1, who also returned a SCQ score = 15 [suggesting relatively subtle parent-reported social-communication features], with paediatrician-assigned ASD diagnosis based on multidisciplinary team assessment, reported to use a primary home language other than English and to display selective mutism).

Many autistic children had floor-level MSEL Early Learning Composite Standard Scores (SS) suggesting pronounced developmental/cognitive delay for a substantial subgroup, but with a distribution of scores otherwise spanning the full possible range (i.e., signalling within and above average abilities for some children in the group). Mean Non-Verbal DQ was low, relative to child age, but significantly greater than Verbal DQ, with medium effect size, *t*(98)=5.34, *p*<.001, *d*=.54. Parent-report MCDI scores (main manuscript Table 2) also spanned the full possible range, indicating some children to be non-/minimally verbal and others able to understand and produce several hundred words and a range of communicative gestures. Finally, group mean level VABS-II Adaptive Behaviour Composite SS differed significantly for children with vs. without autism, with large effect size, *t*(184)=19.07, *p*<.001, *d*=2.80, serving to confirm that the former were broadly ‘typically-developing’ (i.e., adaptive scores broadly within average range) while the latter experienced substantial disability at group mean level (although again with clear within-group variation, including some children scoring within or even above the normative average).

A sizeable subgroup of autistic children (*n*=30) had scores <2SD below the population mean for *both* MSEL DQ and VABS-II Adaptive Behaviour Composite SS suggesting likely co-occurring global developmental delay (GDD)/intellectual disability (ID) alongside autism. Parents reported co-occurring conditions for 36 autistic children—most often GDD (*n*=14), speech/language delay/disorder (*n*=5), and inflammatory/immune (*n*=13) and respiratory conditions (*n*=6). Parents also reported early intervention service access for most autistic children (*n*=89; 11 instances missing)—most often Speech Pathology, Occupational Therapy, and behavioural interventions, as well as attendance at autism-specific day programs—with estimated hours varying widely from 3 to ~2,500 hours to the point of study participation. Medication use—most commonly Melatonin, and asthma/allergy medication—was noted for 28 autistic children, and multivitamin/probiotic use for 24 (7 instances missing data).

Parent-report CBCL data further evidenced children in the Non-Autism Group to be broadly typically-developing, with mean scores for each DSM-oriented scale well within the low normative range. A small subgroup of 10 did score in the Borderline/Clinical Range of concern on one/two CBCL scales (Depression *n*=4; Anxiety *n*=2; ADHD *n*=2, Oppositional Defiance *n*=4), but none scored above the low ‘normal’ range for that related to Autism Spectrum behaviours. There was a significant two-way—child Group (Autism/Non-Autism) by domain (five DSM-oriented scales)—interaction with large effect size, *F*(4,724)=64.93, *p*<.001, *η^2^*=.075, reflecting particularly high mean score for autistic vs. non-autistic children on CBCL Autism Spectrum domain. Autistic children otherwise had group mean-level scores within the low ‘normal’ range across all other CBCL DSM-oriented scales, but significantly elevated compared to non-autistic children. Again there was substantial heterogeneity among autistic children (including only 11 scoring withing the low ‘normal’ range across all five DSM-oriented scales; and subgroups with ‘clinical’ range scores for Depression [*n*=33], Anxiety [*n*=21], ADHD [*n*=18] and Oppositional Defiance [*n*=16]).

Table AF6. Clinical phenotyping data for final participant sample of children, by site

|  | **Melbourne** | | | | **Perth** | | | | **North West Tasmania** | | | |
| --- | --- | --- | --- | --- | --- | --- | --- | --- | --- | --- | --- | --- |
|  | **Autism Group** | | **Non-Autism Group** | | **Autism Group** | | **Non-Autism Group** | | **Autism Group** | | **Non-Autism Group** | |
|  | **n** | **M (SD) range** | **n** | **M (SD) range** | **n** | **M (SD) range** | **n** | **M (SD) range** | **n** | **M (SD) range** | **n** | **M (SD) range** |
| SCQ Total Score | 37 | 19.22 (7.01) 5-31 | 37 | 4.19 (2.59) 0-9 | 29 | 19.83 (5.83) 5-28 | 60 | 3.23 (2.41) 0-10 | 22 | 18.59 (4.93) 10-25 | 3 | 2.33 (2.52) 0-5 |
| VABS ABC SS | 37 | 76.2 (12.1) 54-108 | 34 | 109.2 (13.0) 82-138 | 28 | 77.4 (11.4) 61-107 | 57 | 111.9 (13.8) 80-139 | 22 | 74.0 (13.1) 50-103 | 2 | 126 (7.1) 121-131 |
| CBCL T-Score | 31 |  | 35 |  | 28 |  | 59 |  | 21 |  | 3 |  |
| Depression |  | 62.03 (10.13) 50-84 |  | 53.00 (5.12) 50-70 |  | 65.32 (9.17) 52-86 |  | 53.07 (4.56) 50-67 |  | 67.95 (11.94) 51-95 |  | 50.67 (0.58) 50-51 |
| Anxiety |  | 58.42 (10.20) 50-85 |  | 51.89 (6.30) 50-85 |  | 64.39 (12.22) 50-90 |  | 52.29 (3.74)) 50-63 |  | 60.05 (10.11) 50-82 |  | 50.00 (0.00) None |
| Autism Spectrum |  | 71.36 (9.79) 50-94 |  | 51.23 (3.12) 50-64 |  | 74.36 (7.53) 54-85 |  | 51.36 (2.51) 50-61 |  | 77.52 (7.67) 58-93 |  | 50.00 (0.00) None |
| ADHD |  | 59.23 (8.76) 50-76 |  | 51.57 (3.38) 50-64 |  | 61.12 (6.45) 50-76 |  | 52.31 (4.84) 50-76 |  | 65.43 (8.70) 51-76 |  | 51.00 (1.00) 50-52 |
| Oppositionality |  | 56.77 (7.72) 50-77 |  | 51.51 (2.74) 50-59 |  | 60.39 (9.08) 50-80 |  | 53.19 (5.15) 50-73 |  | 62.95 (10.40) 50-80 |  | 50.33 (0.58) 50-51 |
| ADOS-2 | 39 |  |  |  | 33 |  |  |  | 23 |  |  |  |
| Overall CSS |  | 7.49 (1.52) 4-10 | - | - |  | 7.30 (1.57) 3-10 | - | - |  | 7.91 (1.78) 4-10 | - | - |
| Social Affect |  | 12.31 (3.76) 3-20 | - | - |  | 11.61 (4.19) 5-20 | - | - |  | 12.65 (3.96) 5-18 | - | - |
| Restricted/Repet. |  | 5.05 (1.84) 2-8 | - | - |  | 4.91 (1.83) 2-8 | - | - |  | 4.96 (1.89) 1-8 | - | - |
| MSEL | 39 |  |  |  | 34 |  |  |  | 23 |  |  |  |
| ELC SS |  | 70.69 (22.45) 49-134 | - | - | (32) | 65.78 (21.79) 49-117 | - | - | (22) | 59.77 (16.87) 49-117 | - | - |
| Non-Verbal DQ |  | 74.35 (20.67) 27-117 | - | - |  | 70.59 (20.94) 38-116 | - | - |  | 62.15 (21.79) 26-121 | - | - |
| Verbal DQ |  | 64.54 (34.24) 16-137 | - | - | (33) | 63.71 (26.99) 14-119 | - | - | (22) | 51.98 (24.81) 13-98 | - | - |
| MCDI | 29 |  |  |  | 28 |  |  |  | 19 |  |  |  |
| Receptive Voc. |  | 333.6 (202.7) 18-674 | - | - |  | 419.4 (197.4) 6-678 | - | - |  | 292.4 (219.4) 12-678 | - | - |
| Expressive Voc. |  | 254.1 (225.9) 0-673 | - | - |  | 352.5 (225.7) 0-678 | - | - |  | 229.0 (230.1) 0-678 | - | - |
| Total Gestures |  | 36.13 (16.73) 0-61 | - | - |  | 42.04 (13.80) 3-60 | - | - |  | 41.84 (12.42) 9-61 | - | - |

*Note.* SCQ = Social Communication Questionnaire^7^; VABS ABC SS = Vineland Adaptive Behavior Scales-2^nd^ edition^8^ Adaptive Behaviour Composite Standard Score; CBCL = Child Behaviour Checklist^9^; ADOS-2 CSS = Autism Diagnostic Observation Schedule – 2nd Edition^10, 11^ Calibrated Severity Score^15^; MSEL = Mullen Scales of Early Learning^13^; ELC SS = Early Learning Composite Standard Score; DQ = Developmental Quotient; MCDI = McArthur-Bates Communicative Development Inventories^14^.

# **Details of *Gazefinder* Assessment Feasibility**

**Attempts at *Gazefinder* assessment and calibration**

One or more attempts were made to complete *Gazefinder* assessment with each child (except in the case where adverse responses to other assessment tasks were observed; see below), including repeated attempts when:

- Poor calibration on two consecutive (automated) attempts by *Gazefinder* required a manual restart; or
- Poor child attention or dysregulated behaviour—before, during or after calibration attempt—suggested the possible benefit of re-attempting *Gazefinder* later and/or in a different location (i.e., when/where the child might be more settled and attentive; but again see adverse event descriptions below).

As shown in Figure 1 of the main manuscript, almost all children in the Non-Autism Group (*n*=98) were successfully calibrated on the first attempt with a second assessment/calibration attempt was required for few (n=3) before proceeding to stimulus presentation. Of the 100 autistic children with whom *Gazefinder* assessment was attempted, most were successfully calibrated on the first attempt (*n*=79) with some requiring two (*n*=10), or three attempts (*n*=7). Further to the two children in this group with whom we did not attempt *Gazefinder* (due to adverse event experiences earlier in session), we could not complete calibration for another four (even with multiple attempts), so *Gazefinder* assessment was discontinued with no data recorded.

Post-hoc exploratory analysis indicated that autistic children requiring three attempts for successful calibration had lower NVDQ (*M*=50.92, *SD*=7.13) than those requiring only one or two attempts (*M*=70.64, *SD*=21.94; *F*[2,97]=3.87, *p*=.024, *η^2^*=.074). Autistic children who successfully calibrated on the first attempt had significantly lower CBCL ADHD scores (*M*=60.48, *SD*=7.62) than those requiring a second attempt (*M*=68.30, *SD*=8.95; *F*[2.80]=4.19, *p*=.019, *η^2^*=.095; with intermediate scores for those requiring a third attempt, *M*=60.48, *SD*=7.62). Number of calibration attempts was also associated with overall tracking rate such that autistic children who calibrated successfully on the first or second attempt returned higher overall tracking data (*M*=.78, *SD*=.19) than those requiring an additional attempt (*M*=.51, *SD*=.12; *F*[2,93]=6.85, *p*=.002, *η^2^*=.128).

**Adverse events and other behaviours of note during *Gazefinder* assessment**

We did not expect any risk of psychological, emotional, or other harm to children from completing the non-invasive *Gazefinder* eye-tracking. We considered some children, particularly in the Autism Group, might find some of the clinical/behavioural assessments challenging (as a consequence of their neurodevelopmental condition that includes communication, emotion regulation and associated difficulties) but that the level of risk and potential impact would be no greater than what may arise in regular clinical practice or over the course of daily living. Similarly, we anticipated that some parents of autistic children might experience distress when observing their children having difficulties during the assessment, or that some parents of non-autistic children might become concerned when reflecting on their child’s development while completing questionnaire measures; but again that the risk and potential impact of such occurrences should be no greater than that arising in daily living and regular engagement with community services.

As our primary focus concerned the novel *Gazefinder* eye-tracking assessment, we planned in particular to record any adverse event occurring during a child’s *Gazefinder* assessment and any adverse event occurring subsequently to or enduring beyond the *Gazefinder* assessment session (i.e., as reported by the parent at a short term follow-up contact). Where an adverse event occurred, we filed a written incident report to the La Trobe University Human Research Ethics Committee, including an appraisal of severity of the adverse event, likely relation to the *Gazefinder* eye-tracking assessment (or other aspects of the research assessment protocol), and action taken as a result.

As detailed in Table AF7, a total of seven adverse events during assessment sessions were recorded. These were determined on the basis of researcher observations of child behaviour and all were for children in the Autism Group (6.86% of that recruited sample; no adverse events for children in the Non-Autism Group). None was considered to have been a *Serious Adverse Event*. Researchers also attempted to make follow-up contact with the parents of each participant child, within the few days after *Gazefinder* assessment (or assessment attempt) to enquire about any possible post-assessment or sustained adverse events beyond the assessment session. Four such adverse events were reported after/sustained beyond *Gazefinder* assessment/attempt (also in Table AF7), again all for children in the Autism Group (3.92% of recruited sample), and none determined to have been a *Serious Adverse Event*. Two of these were for children who had experienced an adverse event during the session, while two were children with no within-session adverse event noted.

Table AF7. Detailed record of adverse events during and/or sustained post assessment.

| **Context** | **Severity** | **Appraisal** | **Description of Event and Action Taken** |
| --- | --- | --- | --- |
| ***During assessment session: Related to Assessment Situation Generally*** | | | |
|  | Mild | Probably related | Child showed distress during first structured clinical/behavioural assessment. Remaining clinical tasks and Gazefinder assessment discontinued. Parent reported diagnosis of Pathological Demand Avoidance. |
|  | Mild | Probably related | Child extremely distressed, refusing to complete more structured clinical/behavioural assessment or eye-tracking. It was possible to complete the less structured clinical/behavioural assessment although child became slightly distressed during this time when a preferred toy was packed away; settling when allowed to hold this toy. |
|  | Mild | Probably related | Child distressed and dysregulated on entry to assessment room (at childcare centre with parent present). Semi-structured play-based assessment attempted first but quickly discontinued as child was inconsolable. Calmed when allowed to leave assessment area to go to main playroom and assessment (including Gazefinder eye-tracking) reattempted later in that playroom, with parent, staff and another child present. Child reasonably attentive, regularly squirmy (but not distressed) for eye-tracking, calm and briefly attentive for other tasks. |
| ***During assessment session: Specific to Gazefinder eye-tracking assessment*** | | | |
|  | Moderate | Definitely related | Child very distressed ahead of and during Gazefinder assessment; reluctant to enter new room, increasingly agitated/upset when seated before screen (including when on parent’s lap). As child seemed unhappy with the particular room, *Gazefinder* device moved to another room for re-attempted assessment. Child was calm in this room, but became agitated again when parent brought him to sit before screen, so assessment attempt discontinued. Family offered to return the following week. Child then completed Gazefinder eye-tracking assessment with no difficulty. |
|  | Mild | Probably related | Assessment attempted on two separate occasions. Child showed distress (shallow/rapid breath, crying, pushing/kicking) if researchers or parent tried to seat him before Gazefinder. Child showed no interest even if researchers/parent used strategies to encourage him towards it, physically support him (i.e., hold on parent’s lap), or provide free exploration time in assessment room (which child was calm exploring the room but distressed when Gazefinder assessment attempted). No indication child would eventually settle so attempts discontinued. |
|  | Mild | Probably related | Child became distressed during assessment, saying she did not like “lady on the screen” (Still Face image); averting gaze. |
|  | Mild | Probably related | Child initially engaged during Gazefinder presentation but, at first Still Face image, looked briefly to parent for reassurance. At second ’Still Face’, looked to parent, yelled "Let me out of here!" and ran from room. Calmed immediately. Discontinued. |
| ***After or sustained beyond assessment session*** | | | |
|  | Moderate | Possibly related | Parent reported that child awake screaming at night, following Gazefinder assessment, requiring ~30 minutes to calm back to sleep, and re-awakening crying later. Child reported to wake similarly on other nights, and no other concerns/changes in child behaviour noted. |
|  | Mild | Possibly related | Parent reported child continued to be emotionally/behaviourally dysregulated after the visit. Parent did not think this was specifically related to Gazefinder eye-tracking; rather to combined demands of this and other clinical/behavioural aspects of the session. |
|  | Mild | Possibly related | Parent reported child was more emotionally/behaviourally dysregulated than usual after attending the research visit. Specifically, she noted greater-than-usual difficulty transitioning between day-to-day tasks (e.g., going to take a shower). Parent did not think this was specifically related to Gazefinder eye-tracking; rather to combined demands of this and other clinical/behavioural aspects of the session. |
|  | Mild | Unlikely related | Child became upset after Gazefinder assessment—completed on site at early-intervention centre—when he returned with his parent to the classroom. Parent did not think this was specifically related to Gazefinder eye-tracking; rather, child probably expected to go home. |

**Variation in overall tracking rates and association with other characteristics**

The overall tracking rate is the percentage of gaze fixation time to any part of the display during the ‘Scene 10A’ presentation (excluding during between-stimulus attention-grabbing animations). There was a significant between-group difference in mean overall tracking rate with large effect size, *t*(162.26)=5.44, *p*<.001, *d*=.77, and Figure AF8 shows the relative reduction for children with vs. without autism, but also wide within-group variation. One non-autistic child returned almost no tracking data (1.7%) so was excluded from further quantitative analysis; a 3-year-old female who was initially successfully calibrated (to 5/5 points) but then moved frequently during the main presentation. Several autistic children returned relatively low tracking rates, but no post-hoc exclusions were made. Algorithm development involved all available data for *n*=196.

Researchers noted observations of child behaviour that may have impacted upon Gazefinder assessment. These suggested that child behaviour may have impacted Gazefinder assessment for eight non-autistic children (4 male; 4 female) for whom overall tracking rates ranged from 1.7% to 80% (*M*=49.5%) reflecting variability but substantially lower overall tracking than rates for others in this same group (*M*=91.9%; range=74-99.6%). Those child behaviours most often noted were inattention and/or high activity levels. Seven non-autistic children had difficulty maintaining attention to screen (including two looking away often to examiner/parent), and five had high activity levels (i.e., noted frequently to move during assessment). Researchers noted potential impacts of child behaviour for 34 autistic children (25 male; 9 female) whose overall tracking rates ranged from 0 to 73% (*M*=45.5%), again reflecting variability but substantially lower than rates for others in the same group with no behavioural notes recorded (*M*=86.6%; range=58-99%). Here too, the most commonly-noted behaviours were inattention (n=13) and/or high activity level (n=19; including autistic children noted to be highly distracted or disengaged for parts of the assessment, and one autistic child who often looked toward their parent) but notes also reflected emotion regulation (n=7) and behavioural compliance issues (n=3).


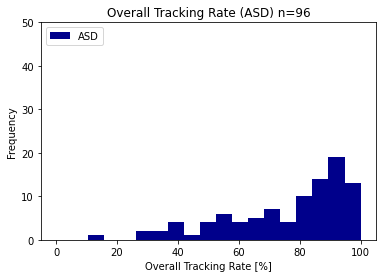

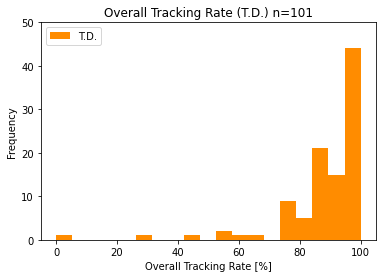


Figure AF8. Distribution of Overall Tracking Rates for groups of autistic and non-autistic children.

# **Details of *Gazefinder* Classification Algorithm**

**Delineation of Candidate Regions of Interest**

A range of possible pre-specified Regions of Interest (ROIs) were available to contribute to the final Gazefinder Classification Algorithm—across 17 stimulus trials, 99 individual candidate ROIs, and 8 sets of paired ROIs (e.g., pointing hand + target object within joint attention trials; face of both adult + child within a social scene, etc.). Furthermore, three candidate durations were defined: 1) full duration of each stimulus presentation (varying of different stimulus trials; range 2 to 8 seconds; see Table AF1); and shorter periods of 2) first 1-second from the start time of each stimulus presentation; and 2) first 2-seconds from the start time of each stimulus presentation. Hence, this offered a total of 321 ROI rate scores for potential inclusion in the final Gazefinder Classification Algorithm (i.e., 99 individual + 8 paired ROIS * 3 durations). Among these, the final set retained for the Gazefinder Classification Algorithm were ROIs that met the following criteria:

- Correlation ≥|.25| of gaze fixation with child group membership (Autism Group = 1; Non-Autism Group = 0); and
- Independence of other ROIs also included in the algorithm (i.e., where multiple candidate ROIs from the same stimulus/trial met criterion A, the ROI—or ROI paired set—contributing to a best-fit algorithm was chosen, with an iterative process to determine the combination yielding best fit).

Table AF8. Association of gaze fixations to all possible candidate ROIs with child group.

| **Trial Type and Number** | **ROI** | **Full Stimulus Duration** |  | **Partial Stimulus Duration** | | |
| --- | --- | --- | --- | --- | --- | --- |
|  |  |  |  | **First 1 sec.** |  | **First 2 sec.** |
| Overall Tracking Rate |  | **-0.340** |  | *N/A* |  | N/A |
| People vs. Geometry (Matched size) Trial A | *People* | **-0.281** |  | -0.219 |  | -0.209 |
|  | *Geometry* | **0.292** |  | 0.159 |  | 0.132 |
|  | *Boy’s Face* | -0.179 |  | 0.040 |  | -0.083 |
|  | *Girl’s Face* | -0.126 |  | -0.077 |  | -0.019 |
|  | *Mother’s Face* | -0.223 |  | -0.163 |  | -0.199 |
|  | *Shape Top Left* | 0.162 |  | 0.109 |  | -0.003 |
|  | *Shape Top Right* | 0.162 |  | 0.015 |  | 0.118 |
|  | *Shape Bottom Left* | 0.172 |  | 0.001 |  | 0.040 |
|  | *Shape Bottom Right* | 0.105 |  | 0.101 |  | 0.103 |
| People vs. Geometry (Matched size) Trial B | *People* | **-0.269** |  | -0.177 |  | -0.198 |
|  | *Geometry* | **0.266** |  | 0.087 |  | 0.160 |
|  | *Girl’s Face* | -0.228 |  | -0.023 |  | -0.126 |
|  | *Infant’s Face* | -0.092 |  | -0.216 |  | -0.135 |
|  | *Shape Top Left* | 0.131 |  | 0.025 |  | 0.065 |
|  | *Shape Mid Right* | 0.191 |  | 0.086 |  | 0.179 |
|  | *Shape at Bottom* | 0.171 |  | 0.004 |  | 0.110 |
| Human Face: Blinking | *Eyes* | -0.044 |  | 0.012 |  | 0.048 |
|  | *Mouth* | -0.132 |  | -0.069 |  | -0.216 |
|  | *Face* | -0.231 |  | -0.080 |  | -0.166 |
| Human Face: Mouth Moving | *Eyes* | 0.028 |  | -0.089 |  | 0.005 |
|  | *Mouth* | -0.335 |  | -0.327 |  | **-0.368** |
|  | *Face* | -0.242 |  | -0.274 |  | -0.306 |
| Human Face: Static/Silent (Still Face) Trial A | *Eyes* | -0.186 |  | -0.083 |  | -0.156 |
|  | *Mouth* | -0.178 |  | -0.176 |  | -0.116 |
|  | *Face* | **-0.269** |  | -0.177 |  | -0.235 |
| Joint Attention (Hand only) Trial A | *Target (Apple)* | -0.278 |  | -0.213 |  | -0.219 |
|  | *Finger* | -0.119 |  | -0.097 |  | -0.106 |
|  | *Distractor (Triangle)* | 0.126 |  | 0.043 |  | 0.074 |
|  | *Distractor (Hexagon)* | 0.069 |  | -0.011 |  | 0.038 |
|  | *Whole Hand* | -0.077 |  | 0.064 |  | -0.124 |
|  | *Target + Finger* | **-0.301** |  | -0.215 |  | -0.237 |
| People vs. Geometry (Embedded) Trial A | *Geometry* | 0.160 |  | 0.064 |  | 0.089 |
|  | *Mother’s Face* | -0.139 |  | -0.072 |  | -0.135 |
|  | *Child’s Face* | -0.056 |  | 0.073 |  | 0.057 |
|  | *Mother’s Eyes* | 0.068 |  | 0.031 |  | 0.036 |
|  | *Child’s Eyes* | -0.099 |  | 0.073 |  | 0.066 |
|  | *Mother’s + Child’s Face* | -0.160 |  | -0.069 |  | -0.132 |
| People vs. Geometry (Matched size) Trial C | *People* | **0.267** |  | 0.156 |  | 0.177 |
|  | *Geometry* | **-0.276** |  | -0.199 |  | -0.200 |
|  | *Child’s Face* | -0.284 |  | -0.239 |  | -0.189 |
|  | *Mother’s Face* | -0.156 |  | 0.054 |  | -0.094 |
|  | *Child’s Eyes* | -0.201 |  | -0.183 |  | -0.197 |
|  | *Mother’s Eyes* | -0.020 |  | 0.050 |  | 0.001 |
|  | *Center of Geometry* | 0.198 |  | 0.133 |  | 0.112 |
| People vs. Geometry (Matched size) Trial D | *People* | **0.294** |  | 0.256 |  | 0.274 |
|  | *Geometry* | **-0.297** |  | -0.290 |  | -0.313 |
|  | *Center of Geometry* | 0.211 |  | 0.110 |  | 0.181 |
|  | *Infant’s Face* | -0.252 |  | -0.212 |  | -0.260 |
| Human Face: Static/Silent (Still Face) Trial B | *Eyes* | 0.035 |  | -0.058 |  | 0.044 |
|  | *Mouth* | **-0.322** |  | -0.098 |  | -0.286 |
|  | *Face* | -0.240 |  | -0.103 |  | -0.198 |
| Human Face: Talking | *Eyes* | 0.106 |  | -0.019 |  | 0.051 |
|  | *Mouth* | **-0.324** |  | -0.248 |  | -0.343 |
|  | *Face* | -0.177 |  | -0.190 |  | -0.189 |
|  |  |  |  |  |  |  |
| Joint Attention (Full torso) Trial B | *Distractor (Square)* | 0.073 |  | 0.098 |  | 0.051 |
|  | *Target (Penguin)* | -0.164 |  | -0.046 |  | -0.249 |
|  | *Finger (Before Pointing)* | 0.004 |  | -0.096 |  | -0.019 |
|  | *Finger (Pointing)* | -0.134 |  | -0.074 |  | -0.105 |
|  | *Agent (Human) Face* | -0.071 |  | -0.040 |  | 0.056 |
|  | *Human Torso* | 0.052 |  | 0.005 |  | 0.108 |
|  | *Target (Penguin) Face* | -0.186 |  | -0.004 |  | -0.219 |
|  | *Target + Finger (Pointing)* | -0.205 |  | -0.070 |  | **-0.290** |
| People vs. Geometry (Embedded) Trial B | *Geometry* | 0.174 |  | 0.179 |  | 0.208 |
|  | *Mother’s Face* | -0.115 |  | -0.232 |  | -0.213 |
|  | *Child’s Face* | -0.268 |  | -0.201 |  | -0.303 |
|  | *Child’s Eyes* | 0.063 |  | -0.023 |  | -0.002 |
|  | *Mother’s Eyes* | -0.084 |  | -0.006 |  | -0.122 |
|  | *Trees (Top Left)* | -0.066 |  | -0.070 |  | -0.028 |
|  | *Mother’s + Child’s Face* | -0.261 |  | -0.275 |  | **-0.328** |
| People vs. Geometry (Matched size) Trial E1 | *People* | **-0.289** |  | -0.323 |  | -0.248 |
|  | *Geometry* | 0.037 |  | 0.134 |  | 0.045 |
|  | *Woman’s Eyes* | -0.007 |  | -0.037 |  | -0.035 |
|  | *Woman’s Face* | -0.241 |  | -0.279 |  | -0.223 |
| People vs. Geometry (Matched size) Trial E2 | *People* | 0.033 |  | -0.042 |  | -0.024 |
|  | *Geometry* | **-0.272** |  | -0.106 |  | -0.137 |
|  | *Woman’s Eyes* | -0.078 |  | 0.016 |  | -0.039 |
|  | *Woman’s Face* | -0.229 |  | -0.083 |  | -0.155 |
| People vs. Geometry (Quadrants) Trial F1 | *Geometry Top Left* | 0.113 |  | -0.018 |  | 0.029 |
|  | *People: Two Girls* | -0.121 |  | -0.115 |  | -0.113 |
|  | *People: Child & Adult* | -0.151 |  | -0.037 |  | -0.116 |
|  | *Geometry Bottom Right* | 0.184 |  | 0.216 |  | 0.219 |
|  | *Left-Most Girl’s Face* | -0.107 |  | -0.099 |  | -0.114 |
|  | *Right-Most Girl’s Face* | -0.158 |  | 0.046 |  | -0.074 |
|  | *Boy’s Face* | -0.133 |  | -0.088 |  | 0.001 |
|  | *Adult’s Face* | -0.039 |  | 0.064 |  | -0.034 |
|  | *Tablet/Device* | -0.142 |  | -0.118 |  | -0.160 |
|  | *Left-Most Girl’s Eyes* | -0.047 |  | -0.013 |  | -0.004 |
|  | *Right-Most Girl’s Eyes* | -0.192 |  | 0.018 |  | -0.126 |
|  | *Left-Most Girl’s Mouth* | -0.097 |  | -0.110 |  | -0.151 |
|  | *Right-Most Girl’s Mouth* | -0.135 |  | 0.044 |  | -0.111 |
|  | *Geometry (both panels)* | 0.190 |  | 0.183 |  | 0.193 |
|  | *People (both panels)* | -0.244 |  | -0.150 |  | -0.206 |
| People vs. Geometry (Quadrants) Trial F2 | *People: Two Girls* | -0.163 |  | -0.101 |  | -0.059 |
|  | *Geometry Top Right* | 0.213 |  | 0.139 |  | 0.175 |
|  | *Geometry Bottom Left* | 0.151 |  | 0.179 |  | 0.207 |
|  | *People: Child & Adult* | -0.249 |  | -0.202 |  | -0.311 |
|  | *Left-Most Girl’s Face* | -0.167 |  | -0.107 |  | -0.096 |
|  | *Right-Most Girl’s Face* | -0.075 |  | -0.069 |  | -0.018 |
|  | *Boy’s Face* | -0.168 |  | -0.167 |  | -0.206 |
|  | *Adult’s Face* | -0.150 |  | -0.023 |  | -0.105 |
|  | *Tablet/Device* | 0.020 |  | 0.008 |  | 0.013 |
|  | *Left-Most Girl’s Eyes* | -0.011 |  | -0.004 |  | -0.035 |
|  | *Right-Most Girl’s Eyes* | -0.074 |  | -0.036 |  | -0.015 |
|  | *Left-Most Girl’s Mouth* | -0.208 |  | -0.160 |  | -0.113 |
|  | *Right-Most Girl’s Mouth* | -0.064 |  | -0.066 |  | -0.039 |
|  | *People (both panels)* | **-0.342** |  | -0.273 |  | -0.314 |
|  | *Geometry (both panels)* | 0.229 |  | 0.216 |  | 0.243 |

*Note.* Child group coded Autism = 1, Non-Autism = 0; Biological motion PLD trials excluded from consideration due to past research suggesting no differentiating effect.^3^ Cell-level shading indicates candidate ROIs for classification algorithm, with association ≥|0.25|; Final retained ROIs also in bold typeface.

# **Summary of final set of ROIs retained for G*azefinder* classification algorithm**

The final best-fit algorithm for classifying 2- to 4-year-olds included gaze to ROIs reflecting patterns of data broadly consistent with previous findings concerning social/non-social preferential attention in autism;^22, 23^ *increased* attention to non-social (i.e., geometry) and *reduced* attention to social (i.e., people) scenes, and reduced attention to the human still face and mouth ROIs (e.g., when static and moving/talking; though we did not see particular association with gaze toward eye ROIs as might have been anticipated). The observed *reduced* attention to socially-meaningful stimuli in pointing trials^24^ (i.e., hand/figure and target object) is consistent with robust clinical evidence of joint-attention differences in autism^25^ implicated (alongside differential attention to facial features) in the language-learning difficulties experienced by many young autistic children.^26^ Future work could test *Gazefinder* algorithm generalisability across ages/developmental stages.

| **Overall Tracking Rate *r* = -.340** | **People *r* = -.281; Geometry *r* = .292** | **People *r* = -.269; Geometry *r* = .266** |  | **Mouth *r* = -.368** | **Still Face *r* = -.269** | **Target + Finger**  ***r* = -.301** |  |  |
| --- | --- | --- | --- | --- | --- | --- | --- | --- |
| Autism Group: less gaze across stimulus set 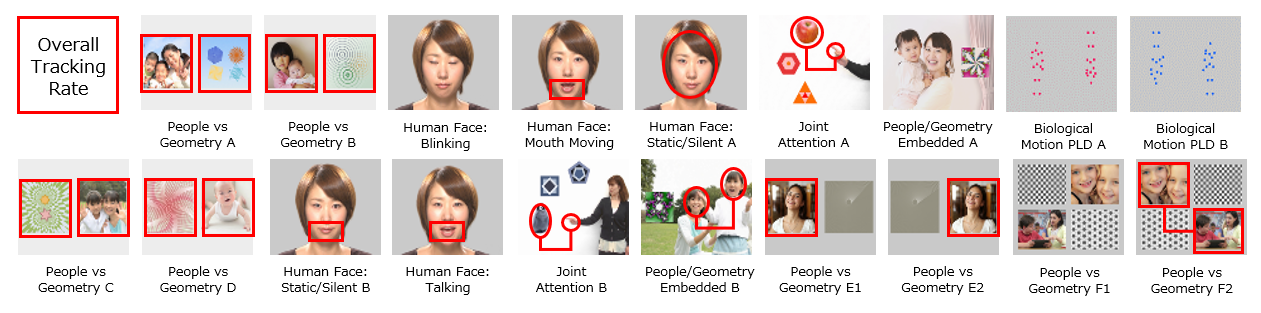 | Autism Group: less gaze to people; more to geometry ROIs | Autism Group: less gaze to people, more to geometry ROIs |  | Autism Group: less gaze to mouth when moving (first 2s only) | Autism Group: less gaze to whole still face (after trials with movement) | Autism Group: less gaze to pointing finger and target |  | (Not considered) |

| **Geometry**  ***r* = .267;**  **People *r* = -.276** | **Geometry**  ***r* = .294;**  **People *r* = -.297** | **Mouth**  ***r* = -.322** | **Mouth**  ***r* = -.324** | **Target + Finger**  ***r* = -.290** | **Mother’s and Child’s Faces**  ***r* = -.328** | **People**  ***r* = -.289** | **People**  ***r* = -.272** |  | **People**  **(2 panels)**  ***r* = -.342** |
| --- | --- | --- | --- | --- | --- | --- | --- | --- | --- |
| Autism Group: less gaze to people, more to geometry ROIs | Autism Group: less gaze to people, more to geometry ROIs | Autism Group: less gaze to mouth during second still face trial | Autism Group: less gaze to mouth when talking | Autism Group: less gaze to pointing finger and target (first 2s only) | Autism Group: less gaze to two face ROIs (first 2s only) | Autism Group: less gaze to person ROI | Autism Group: less gaze to person ROI |  | Autism Group: less gaze to two social ROIs |

Figure AF9. Summary of final ROI set retained for best-fit classification algorithm (with magnitude/direction of association with Group: Autism=1; Non-Autism=0).

Table AF9.

Associations of tracking rate and algorithm score with child characteristics

|  | **Overall Tracking Rate (0-100)** | | | |  | **Classification Algorithm Score (0-100)** | | | |
| --- | --- | --- | --- | --- | --- | --- | --- | --- | --- |
|  | **Cohort** |  | **Autism** | **Non-Autism** |  | **Cohort** |  | **Autism** | **Non-Autism** |
|  | **n = 196** |  | **n = 96** | **n = 100** |  | **n = 196** |  | **n = 96** | **n = 100** |
| Site: Melb./Perth/NWTas | *F*=6.92^***^ |  | *F*=1.35 | *F*=1.54 |  | *F*=3.17^*^ |  | *F*=0.06 | *F*=1.90 |
| Sex at Birth: n Male/Female | *r*= .12 |  | *r*= -.02 | *r*= .02 |  | *r*= -.21^**^ |  | *r*= -.09 | *r*= -.11 |
| Age | *r*= -.07 |  | *r*= -.11 | *r*= .12 |  | *r*= .24^***^ |  | *r*= .06 | *r*= .27^**^ |
| Prematurity: n Term/<37wk | *r*= -.15^*^ |  | *r*= -.20 | *r*= -.08 |  | *r*= .16^*^ |  | *r*= .14 | *r*= .11 |
| Birth Order among Siblings | *r*= -.01 |  | *r*= -.12 | *r*= .13 |  | *r*= .06 |  | *r*= .11 | *r*= -.02 |
| Ethnicity | *r*= -.05 |  | *r*= .16 | *r*= -.04 |  | *r*= .08 |  | *r*= -.02 | *r*= -.12 |
| Family Composition | *r*= -.16* |  | *r*= -.10 | *r*= -.07 |  | *r*= .12 |  | *r*= .05 | *r*= -.04 |
| Nominated Primary Carer | *r*= .00 |  | *r*= -.01 | *r*= -.04 |  | *r*= -.06 |  | *r*= -.10 | *r*= .06 |
| Primary Carer Education | *r*= -.17* |  | *r*= -.24* | *r*= -.14 |  | *r*= -.24** |  | *r*= -.13 | *r*= -.06 |
| Primary Home Language | *r*= -.04 |  | *r*= .13 | *r*= .14 |  | *r*= .02 |  | *r*= -.05 | *r*= -.11 |
| Household Income | *r*= .21** |  | *r*= .09 | *r*= .12 |  | *r*= -.15* |  | *r*= .09 | *r*= -.01 |
| Calibration Attempts: n 1/2+ | *r*= -.15^*^ |  | *r*= -.18 | *r*= .15 |  | *r*= .19^**^ |  | *r*= .13 | *r*= -.04 |
| SCQ Total Score | *r*= -.34^***^ |  | *r*= -.07 | *r*= .01 |  | *r*= .53^***^ |  | *r*= .25^*^ | *r*= .11 |
| VABS ABC SS | *r*= .37^***^ |  | *r*= .05 | *r*= .03 |  | *r*= -.48^***^ |  | *r*= -.21 | *r*= -.01 |
| CBCL Depression | *r*= -.26^***^ |  | *r*= -.10 | *r*= .01 |  | *r*= .35^***^ |  | *r*= .14 | *r*= -.03 |
| CBCL Anxiety | *r*= -.23^**^ |  | *r*= .00 | *r*= -.07 |  | *r*= .23^**^ |  | *r*= -.08 | *r*= .02 |
| CBCL Autism Spectrum | *r*= -.36^***^ |  | *r*= -.04 | *r*= -.04 |  | *r*= .49^***^ |  | *r*= .13 | *r*= .09 |
| CBCL ADHD | *r*= -.34^***^ |  | *r*= -.18 | *r*= -.08 |  | *r*= .30^***^ |  | *r*= .12 | *r*= -.20 |
| CBCL Oppositional Defiance | *r*= -.16^*^ |  | *r*= -.01 | *r*= .03 |  | *r*= .14 |  | *r*= -.18 | *r*= -.08 |
| ADOS Overall CSS | - |  | *r*= -.09 | - |  | - |  | *r*= .17 | - |
| ADOS Social Affect Algorithm | - |  | *r*= -.13 | - |  | - |  | *r*= .40^***^ | - |
| ADOS RRB Algorithm | - |  | *r*= -.16 | - |  | - |  | *r*= .15 | - |
| MSEL ELC SS | - |  | *r*= .29^**^ | - |  | - |  | *r*= -.44^***^ | - |
| MSEL Non-Verbal DQ | - |  | *r*= .29^**^ | - |  | - |  | *r*= -.41^***^ | - |
| MSEL Verbal DQ | - |  | *r*= .21^*^ | - |  | - |  | *r*= -.42^***^ | - |
| MCDI Receptive Vocab. | - |  | *r*= .10 | - |  | - |  | *r*= -.23^*^ | - |
| MCDI Expressive Vocab. | - |  | *r*= .09 | - |  | - |  | *r*= -.22 | - |
| MCDI Total Gestures Score | - |  | *r*= -.04 | - |  | - |  | *r*= -.12 | - |

*Note.* All data are Spearman’s rho unless otherwise indicated. Melb = Melbourne; NWTas = North West regional Tasmania; SCQ = Social Communication Questionnaire^7^; VABS ABC SS = Vineland Adaptive Behavior Scales-2^nd^ edition^8^ Adaptive Behaviour Composite Standard Score; CBCL = Child Behaviour Checklist^9^; ADOS-2 CSS = Autism Diagnostic Observation Schedule – 2nd Edition^10, 11^ Calibrated Severity Score^15^; MSEL = Mullen Scales of Early Learning^13^; ELC SS = Early Learning Composite Standard Score; DQ = Developmental Quotient; MCDI = McArthur-Bates Communicative Development Inventories^14^. ^*^ *p*<.05; ^**^ *p*<.01; ^***^ *p*<.001

# **PERFORMANCE PROPERTIES OF GAZEFINDER ALGORITHM VS. OTHER DIAGNOSTIC TOOLS**

Table AF10.

Summary of performance properties for *Gazefinder* classification algorithm and classification algorithms reported for other gold-standard diagnostic assessment tools, for children of comparable ages and ability levels.

| **Instrument & Version** | | | **Sample Characteristics for Specific Algorithm** | | **Sample Size**  **Autism:Non-Autism** | **Sensitivity** | **Specificity** | **AUC** | **Accuracy** |
| --- | --- | --- | --- | --- | --- | --- | --- | --- | --- |
| Gazefinder Algorithm (~2.5 minute for ‘off the shelf’ use) with “Scene 10A” sequence of social/non-social attention scenes, and threshold score of 28.6 (possible range 0-100) | | | Children 2:00 – 4:11 | | 96:100 | .82 | .70 | .82 | .76 |
| Autism Diagnostic Observation Schedule (ADOS)^10, 11^  45-60 min. semi-structured play-based assessment requiring substantial training for administration and reliable scoring | **Toddler Module** (for children aged 12 to 30 months with limited language) | All children ≤20m and any children >20m with few/no words | | 35:101 | | .91 | .94 | Not reported | |
|  |  | Any children >20m with some words | | 24:47 | | .88 | .94 |  |  |
|  | **Module 1** (for children aged 31+ months with limited language) | Children with few/no words & non-verbal mental age equiv. ≤15m | | 20:16 | | .95 | .19 |  |  |
|  |  | Children with few/no words & non-verbal mental age equiv. >15m | | 51:33 | | .82 | .79 |  |  |
|  |  | Children with some words | | 75:76 | | .77 | .82 |  |  |
|  | **Module 2** (for any young child with phrase speech; usually aged 24+ months) | Children aged <5 years | | 49:30 | | .84 | .77 |  |  |
|  |  | Children aged ≥ 5 years | | N/A (Older than children in the current cohort) | | | | | |
|  | **Module 3** (for any child with fluent speech; usually 4+ years) | One algorithm only | | N/A (Standardisation sample older than current cohort; *M =* 8 years) | | | | | |

Table AF11. Associations of range of socio-demographic characteristics with correct and mis-classification status for autistic ‘cases’ and non-autistic ‘controls’

|  | **Omnibus 4-way Comparison** | **Follow-up Pair-wise Contrasts** | | | **Misclassified Contrast: False Negative v.**  **False Positive** |
| --- | --- | --- | --- | --- | --- |
|  |  | **False Negative v. True Positive** | **True Positive v. True Negative** | **True Negative v. False Positive** |  |
| n | N = 196 | 17 : 79 | 79 : 70 | 70 : 30 | 17 : 30 |
| Sex at Birth (male v. female) | *χ*^2^(3)=14.14^**^ | *χ*^2^(1)=1.54 | *χ*^2^(1)=12.47^***^ | *χ*^2^(1)=0.50 | *χ*^2^(1)=0.25 |
| Age | *F*(3,192)=4.355^**^ | *t*(192)= -0.61 | *t*(192)=3.49^**^ | *t*(192)= -2.21 | *t*(192)= 0.42 |
| Prematurity (full term vs. any pre-term birth) | *χ*^2^(3)=3.94 | - | - | - | - |
| Birth Order among Siblings (only child vs. first born vs. later born) | *χ*^2^(6)=7.16 | - | - | - | - |
| Ethnicity (Caucasian Australian vs. any other) | *χ*^2^(3)=13.05^**^ | *χ*^2^(1)=0.18 | *χ*^2^(3)=12.48^***^ | *χ*^2^(1)=0.52 | *χ*^2^(1)=5.99^*^ |
| Family Composition (dual parent vs. single parent or other adults) | *χ*^2^(3)=16.60^***^ | *χ*^2^(1)=2.45 | *χ*^2^(1)=12.36^***^ | *χ*^2^(1)=0.38 | *χ*^2^(1)=6.63^*^ |
| Nominated Primary Carer (mother vs. father) | *χ*^2^(3)=4.79 | - | - | - | - |
| Primary Carer Education (any primary/secondary vs. degree vs. post-graduate education) | *χ*^2^(6)=21.92^**^ | *χ*^2^(2)=0.85 | *χ*^2^(2)=20.64^***^ | *χ*^2^(2)=0.34 | *χ*^2^(2)=1.83 |
| Primary Home Language (English vs any other) | *χ*^2^(3)=4.57 | - | - | - | - |
| Household Income (categories as per Table AF4) | *χ*^2^(12)=31.08^**^ | *χ*^2^(4)=4.52 | *χ*^2^(4)=24.58^***^ | *χ*^2^(3)=0.38 | *χ*^2^(4)=11.32^*^ |

*Note.* ^*^ *p*<.05; ^**^ *p*<.01; ^***^ *p*<.001

Table AF12.

Summary of Gazefinder autism Classification Algorithm performance, overall and for participant subgroups

| **Summary Classification Algorithm Performance** | | **n** | **Sensitivity** | **Specificity** | **Accuracy** |
| --- | --- | --- | --- | --- | --- |
| Best-Fit Algorithm (Threshold = 28.6) | AUC = .82 | 196 | .82 | .70 | .76 |
| LOOCV (Threshold range = 26.6-30.6) | AUC = .82 | 196 | .81 | .69 | .75 |
| **Subgroup Performance** |  |  |  |  |  |
| **Variables where Groups Differed** | **Level** | **n** | **Sensitivity** | **Specificity** | **Accuracy** |
| Child Sex at Birth | Male | 136 | .85 | .67 | .77 |
|  | Female | 60 | .72 | .74 | .73 |
| Ethnicity | Caucasian Australian | 139 | .82 | .69 | .74 |
|  | All Other | 46 | .78 | .79 | .78 |
| Family Composition | Dual Parent | 172 | .84 | .70 | .76 |
|  | All Other | 17 | .67 | .50 | .65 |
| Primary Carer Education | Primary/Secondary | 27 | .86 | .60 | .81 |
|  | Degree | 60 | .81 | .68 | .75 |
|  | Postgraduate | 100 | .76 | .71 | .73 |
| Household Income | <AUD$25-85,000 | 36 | .79 | .75 | .78 |
|  | AUD$85-115,000 | 43 | .79 | .67 | .72 |
|  | >AUD$115,000 | 93 | .90 | .70 | .76 |
| **Variables where Groups Were Matched** | **Level** | **n** | **Sensitivity** | **Specificity** | **Accuracy** |
| Gestational Age at Birth | Full Term | 173 | .79 | .71 | .75 |
|  | Any Pre-Term | 14 | 1.00 | .50 | .79 |
| Birth Order among Siblings | Only Child | 56 | .67 | .69 | .68 |
|  | First Born | 55 | .90 | .62 | .76 |
|  | Later Born | 78 | .84 | .76 | .79 |
| Nominated Primary Caregiver | Mother | 176 | .82 | .71 | .76 |
|  | Father | 10 | .33 | .57 | .50 |
| Primary Home Language | English | 180 | .82 | .69 | .75 |
|  | All Other | 9 | .71 | 1.00 | .78 |

# **REFERENCES**

1. JVCKENWOOD Corporation (JKC). *Gazefinder Instruction Manual: Operation*. Kanagawa: Author; 2022. p. 68.

2. JVCKENWOOD Corporation (JKC). *Gazefinder Instruction Manual: Product Specifications/Safety Precautions*. Kanagawa, Japan: Author; 2022. p. 32.

3. Fujioka T, Inohara K, Okamoto Y, et al. Gazefinder as a clinical supplementary tool for discriminating between autism spectrum disorder and typical development in male adolescents and adults. *Mol Autism*. 2016;7(19):19. doi:10.1186/s13229-016-0083-y

4. Annaz D, Campbell R, Coleman M, Milne E, Swettenham J. Young children with autism spectrum disorder do not preferentially attend to biological motion. *J Autism Dev Disord*. 2012;42(3):401-408. doi:10.1007/s10803-011-1256-3

5. Klin A, Lin D, Gorrindo P, Ramsay G, Jones W. Two-year-olds with autism orient to non-social contingencies rather than biological motion. *Nature*. 2009;459:257-261.

6. Wang L, Chien S, Hu S, Chen T, Chen H. Children with autism spectrum disorders are less proficient in action identification and lacking a preference for upright point-light biological motion displays. *Res Autism Spec Dis*. 2015;11:63-76.

7. Rutter M, Bailey A, Lord C, Berument S, Pickles A. *Social Communication Questionnaire*. Western Psychological Services; 2003.

8. Sparrow S, Cicchetti D, Balla D. *The Vineland Adaptive Behavior Scales, 2nd Edition (VABS-II)*. NCS Pearson Inc; 2005.

9. Achenbach T, Rescorla L. *Child Behavior Checklist*. 2000.

10. Lord C, Luyster R, Gotham K, Guthrie W. *Autism Diagnostic Observation Schedule, 2nd Edition (ADOS-2) Manual (Part II): Toddler Module*. Western Psychological Services; 2012.

11. Lord C, Rutter M, DiLavore P, Risi S, Gotham K, Bishop S. *Autism Diagnostic Observation Schedule, 2nd Edition (ADOS-2)*. Western Psychological Services; 2012.

12. Gotham K, Pickles A, Lord C. Standardizing ADOS Scores for a measure of severity in Autism Spectrum Disorders. *J Autism Dev Disord*. 2009;39:693-705.

13. Mullen E. *Mullen Scales of Early Learning*. American Guidance Service; 1995.

14. Fenson L, Dale P, Reznick J. *The MacArthur Communicative Development Inventories: User’s Guide and Technical Manual*. Singular; 1993.

15. Esler A, Bal V, Guthrie W, Wetherby A, Weismer S, Lord C. The autism diagnostic observation schedule, toddler module: standardized severity scores. *J Autism Dev Disord*. 2015;45(9):2407-2720.

16. Loomes R, Hull L, Mandy W. What is the male-to-female ratio in autism spectrum disorder? A systematic review and meta-analysis. *J Am Acad Child Psy*. 2017;56(6):466-474.

17. Welfare AIoHa. *Australia’s mothers and babies 2018: In brief*. 2020. *Perinatal Statistics*. [www.aihw.gov.au/reports/mothers-babies/australias-mothers-babies-data-visualisations/contents/baby-outcomes/gestational-age](https://latrobeuni-my.sharepoint.com/personal/khudry_ltu_edu_au/Documents/Desktop/www.aihw.gov.au/reports/mothers-babies/australias-mothers-babies-data-visualisations/contents/baby-outcomes/gestational-age)

18. Estimates of Aboriginal and Torres Strait Islander Australians, June 2016. (Author) (2018).

19. Qu L. *Households and Families*. 2020:14. *Australian Families Then and Now*. <https://aifs.gov.au/sites/default/files/publication-documents/2007_aftn_households_and_families.pdf>

20. Statistics ABo. Qualifications and Work: Detailed information about the educational qualifications people have studied and their relevance to current jobs (2018-2019). Author. Updated 29.09.2020. Accessed 30/03, 2021. <https://www.abs.gov.au/statistics/people/education/qualifications-and-work/2018-19>

21. Davidson P, Bradbury B, Wong M. *Poverty in Australia 2020: Part 2, Who is affected?* 2020. *ACOSS/UNSW Poverty and Inequality Partnership Report No 4*. <http://povertyandinequality.acoss.org.au/wp-content/uploads/2020/05/Poverty-in-Australia-2020-Part-2-%E2%80%93-Who-is-affected_Final.pdf>

22. Papagiannopoulou E, Chitty K, Hermens D, Hickie I, Lagopoulos J. A systematic review and meta-analysis of eye-tracking studies in children with autism spectrum disorders. *Soc Neurosci*. 2014;9:610-632.

23. Pierce K, Marinero S, Hazin R, McKenna B, Barnes C, Malige A. Eye tracking reveals abnormal visual preference for geometric images as an early biomarker of an autism spectrum disorder subtype associated with increased symptom severity. *Biol Psychiat*. 2016;15:657-666.

24. Bedford R, Elsabbagh M, Gliga T, et al. Precursors to social and communication difficulties in infants at-risk for autism: gaze following and attentional engagement. *J Autism Dev Disord.* 2012;42:2208-2218.

25. Association AP. *Diagnostic and statistical manual of mental disorders - Fifth Edition (DSM-5)*. 5 ed. American Psychiatric Publishing; 2013.

26. Hudry K, Chandler S, Bedford R, et al. Early language profiles in infants at high-risk for autism spectrum disorders. *J Autism Dev Disord*. 2014;44(154-167)
